# Quercetin improves epithelial regeneration from airway basal cells of COPD patients

**DOI:** 10.1186/s12931-024-02742-0

**Published:** 2024-03-11

**Authors:** Elizabeth S. McCluskey, Nathan Liu, Abhimaneu Pandey, Nathaniel Marchetti, Steven G. Kelsen, Umadevi S. Sajjan

**Affiliations:** 1 Center for Inflammation and Lung Research, Lewis-Katz Medical School, Temple University, Philadelphia, PA 19140 USA; 2 Department of Microbiology, Immunology and Inflammation, Lewis-Katz Medical School, Temple University, Philadelphia, PA 19140 USA; 3 Department of Thoracic Medicine and Surgery, Temple University Health System, Philadelphia, PA 19140 USA

**Keywords:** HOXB2, ELF3, Goblet cell metaplasia, Chronic obstructive pulmonary disease

## Abstract

**Background:**

Airway basal cells (BC) from patients with chronic obstructive pulmonary disease (COPD) regenerate abnormal airway epithelium and this was associated with reduced expression of several genes involved in epithelial repair. Quercetin reduces airway epithelial remodeling and inflammation in COPD models, therefore we examined whether quercetin promotes normal epithelial regeneration from COPD BC by altering gene expression.

**Methods:**

COPD BC treated with DMSO or 1 µM quercetin for three days were cultured at air/liquid interface (ALI) for up to 4 weeks. BC from healthy donors cultured at ALI were used as controls. Polarization of cells was determined at 8 days of ALI. The cell types and IL-8 expression in differentiated cell cultures were quantified by flow cytometry and ELISA respectively. Microarray analysis was conducted on DMSO or 1 µM quercetin-treated COPD BC for 3 days to identify differentially regulated genes (DEG). Bronchial brushings obtained from COPD patients with similar age and disease status treated with either placebo (4 subjects) or 2000 mg/day quercetin (7 subjects) for 6 months were used to confirm the effects of quercetin on gene expression.

**Results:**

Compared to placebo-, quercetin-treated COPD BC showed significantly increased transepithelial resistance, more ciliated cells, fewer goblet cells, and lower IL-8. Quercetin upregulated genes associated with tissue and epithelial development and differentiation in COPD BC. COPD patients treated with quercetin, but not placebo showed increased expression of two developmental genes HOXB2 and ELF3, which were also increased in quercetin-treated COPD BC with FDR < 0.001. Active smokers showed increased mRNA expression of TGF-β (0.067) and IL-8 (22.0), which was reduced by 3.6 and 4.14 fold respectively after quercetin treatment.

**Conclusions:**

These results indicate that quercetin may improve airway epithelial regeneration by increasing the expression of genes involved in epithelial development/differentiation in COPD.

**Trial registration:**

This study was registered at ClinicalTrials.gov on 6-18-2019. The study number is NCT03989271.

**Supplementary Information:**

The online version contains supplementary material available at 10.1186/s12931-024-02742-0.

## Introduction

Epithelium lining the conductive airways protects the lungs from environmental pollutants and pathogens through mucociliary escalator function and mounting appropriate innate immune responses [1, 2]. Chronic inflammation caused by persistent exposure to environmental insults such as cigarette smoke or other noxious agents may lead to airway epithelial remodeling [3–5], which can affect innate immune protective mechanisms [6]. In patients with chronic obstructive pulmonary disease (COPD), airway epithelium often shows basal cell hyperplasia, squamous metaplasia and goblet cell metaplasia and such structural changes affects innate immune responses [7]. Previously, we have shown that in a mouse model of COPD, airway epithelium shows goblet cell metaplasia, which is reduced by oral treatment with quercetin [8, 9].

Airway basal cells (BC) of the tracheobronchial tree are specialized tissue-specific stem cells and generate all cell types of the airway epithelium during turnover or repair [7, 10, 11]. Interestingly, epithelium regenerated from BC isolated from COPD patients shows goblet cell hyperplasia resembling airway epithelium in COPD patients [12–15]. These observations indicate that COPD BC may have defects in the repair and regeneration program. Consistent with this, recently we showed that compared to healthy non-smokers, BC from COPD patients show attenuated expression of some transcription factors involved in tissue development and differentiation, which include homeobox (HOX)A1, HOXB2, E74-like ETS transcription factor (ELF3), ELF5 and vestigial like family member (VGLL)1 [16]. The observed differential expression may be due to acquired epigenetic changes, which may occur as a result of chronic exposure of BC to inflammatory environment induced by cigarette smoke or other noxious gases. Since quercetin reduces airway epithelial remodeling including goblet cell metaplasia in a mouse model of COPD [8, 9], we examined whether quercetin improves airway epithelial regeneration from COPD BC in vitro by altering the expression of genes involved in tissue development and differentiation.

Quercetin (3,3’,4’,5,7-pentahydroxyflavone) is a dietary flavonoid found in many plants [17]. At the molecular level, quercetin scavenges reactive oxidant species, thus acting as antioxidant [18], inhibits various kinases exerting anti-inflammatory effects [19], protects cells from oxidative stress-induced DNA damage [20] and regulates epigenetic changes by modulating DNA methyltransferases, histone deacetylases and long non-coding RNAs [21, 22]. Recently, quercetin was also demonstrated to promote osteogenic differentiation of bone marrow mesenchymal stem cells and epidermal stem cell proliferation by modulating Wnt/β-catenin signaling [23, 24]. BC from COPD smokers which have impaired capacity to regenerate mucociliary-differentiated airway epithelium in vitro showed hypermethylation of 75 probe sets and hypomethylation of 202 probe sets. Ingenuity pathway analysis of 75 hypermethylated genes indicated enrichment in cellular growth and proliferation [25]. Given the capacity of quercetin to reduce airway remodeling in COPD mouse models [8, 9], promote cellular proliferation and differentiation [23, 24], and modulate epigenetic changes [21, 22], we postulated that quercetin may improve regeneration of airway epithelium from BC by modulating the genes involved in epithelial cell proliferation and differentiation.

In this study we examined whether quercetin promotes normal regeneration of airway epithelium from COPD BC. Secondly, we also examined whether quercetin-induced changes are associated with modulation of gene expression. Our study demonstrates that treatment with quercetin for 3 days enhances the expression of various developmental genes in COPD BC and promotes polarization and differentiation of COPD BC towards ciliated cells. Interestingly, we also demonstrate that bronchial brushings from COPD patients treated with quercetin for 6 months show increased expression of two developmental genes, HOXB2 and ELF3 which may play a role in the polarization and differentiation of airway epithelial cells.

## Methods

### Quercetin

For cell culture studies, quercetin was kindly provided by Quercegen Pharmaceuticals (Sudbury, MA). HPLC analysis indicated that quercetin was 99% pure. 100mM quercetin was prepared in sterile DMSO and stored in aliquots at -80 °C for up to three days. Previous studies have demonstrated that quercetin dissolved in DMSO is stable for up to 2 weeks [26]. On the day of use quercetin was dilute to 1 µM in the cell culture medium, filter sterilized and used immediately. Similar volume of DMSO diluted in cell culture medium was used as a vehicle control.

For the clinical trial, quercetin and placebo formulations were purchased from Nutravail Technologies (Chantily, VA) as soft chews. Each quercetin chew contained 500 mg of quercetin, 350 mg of ascorbic acid and 10 mg of niacin. Each placebo chew had 350 mg of ascorbic acid and 10 mg of niacin.

### Isolation and culturing of airway epithelial cells

BC were isolated from bronchial segments of normal donor lungs and explanted lungs from COPD patients at the time of lung transplantation as described previously [12, 27]. The collection of the tissue was approved by the Institutional Review Board of University Michigan, Ann Arbor, MI (HUM00052806) and Temple University, Philadelphia, PA (4407). Patient characteristics are provided in Supplemental Table 1. The BC at passage one, were cultured in 6.5 mm collagen-coated transwells as described previously [27, 28]. In some experiments, 80–90% confluent COPD BC cultured in 6.5 mm transwells were treated with quercetin or equal volume of DMSO (vehicle) for three consecutive days from both apical and basolateral sides as indicated in the results section. The cells were then harvested for isolation of total RNA or cultured at air/liquid interface (ALI) for up to 4 weeks to promote polarization and mucociliary differentiation of cells.

### Collection of bronchial brushings from COPD patients

Clinical trial with quercetin in COPD patients was registered at Clinicaltrial.gov # NCT03989271. The clinical trial was conducted “in accordance with Declaration of Helsinki”. The patients provided consent to participate in the clinical trial and also for publishing the results obtained from the samples collected. The clinical trial with quercetin and collection of Bronchial brush cells were approved by Temple University Institutional Regulatory Board #25,738. All the patient data is deidentified. Patient characteristics are presented in Supplemental Table 2. In this clinical trial, patients were treated with a placebo or 2000 mg/day quercetin for 6 months. The study drug was dispensed once in three months (90 days) and the patients took 4 placebo or quercetin chews per day; 2 in the morning and 2 in the evening. Compliance was calculated by the following equation. %Compliance = 100 × (360-Number of chews returned)/360 (dispensed number of chews). Post-bronchodilator FEV1 was measured and bronchial brushings collected from 4 placebo and 7 quercetin-treated COPD patients at baseline and at the end of the treatment. The cells from the bronchial brushings were harvested by centrifugation, lysed in TRIZOL and stored at -80° C. The total RNA was isolated from all the samples at the same time. Blood was collected and analyzed for quercetin levels by reverse phase HPLC as described previously [29].

### Transepithelial resistance

Transepithelial resistance (TER) was measured using the EVOM voltmeter equipped with ENDOHM-6 EVOM electrode (World Precision Instruments, Sarasota, FL) as previously described and the TER was expressed as Ohms/cm^2^ [30, 31].

### Cytotoxicity assay

Cytotoxicity of quercetin in cell cultures was measured by determining the Lactose dehydrogenase (LDH) activity in the basal medium using CytoTox96 Non-radioactive cytotoxicity assay kit from Promega (Madison, WI).

### ELISA

After culturing the cells for 4 weeks at ALI, transwells were transferred to a new receiver plate containing fresh medium and incubated for 24 h. The basolateral medium was collected and IL-6 and IL-8 protein levels were measured by ELISA (R & D systems, Minneapolis, MN).

### Total RNA isolation

Total RNA was isolated from TRIZOL lysates of normal BC, DMSO and quercetin-treated COPD BC, and bronchial brushings using a Direct-zol™ miniprep kit (Zymo research, Irvine, CA). The integrity of total RNA was determined by Agilent 2100 bioanalyzer and the RNA integrity number was consistently > 7.

### Microarray processing

Biotinylated cDNAs synthesized from total RNA isolated from DMSO- or quercetin-treated COPD BC were subjected to microarray analysis using Human Gene 2.1 ST arrays. To identify statistically significant differential gene regulation (p-value of < 0.05 and up/down regulated by more than 2-fold), we performed pairwise comparison (DMSO versus quercetin) by the LIMMA methodology (Linear Models for Microarray Data) [32]. Normalized data and raw data are available in Gene Expression Omnibus (GEO) with accession number GSE253052.

### Gene Ontology

Gene Ontology (GO) and KEGG pathways were analyzed with WebGestalt (WEB-based GEne SeT AnaLysis Toolkit) [33] using the Benjamini-Hochberg correction for multiple testing (FDR 5%). For Gene Ontology, only Biological Process terms are discussed because Cellular Component and Molecular Function terms were less relevant.

### Flow cytometry

Mucociliary-differentiated cells were dissociated with accutase (Innovative cell technologies, San Diego, CA), cells were then incubated with Zombie-UV™ fixable dye (Biolegend, San Diego, CA), fixed with 4% paraformaldehyde, and then blocked/permeabilized by incubating in PBS containing 1% BSA and 0.5% saponin for 30 min. Cells were then incubated with normal mouse IgG (control), or antibodies to acetylated tubulin (cat#T7451, Sigma Aldrich, St. Louis, MO), Muc5AC (cat#ab24071TP63, Abcam, Cambridge, MA) or TP63 (Cat#ab32353, Abcam), and bound antibodies were detected by using FITC-labeled second antibodies as previously described [16]. The cells were then analyzed in FACS Caliber Flow cytometer (BD Biosciences, San Jose, CA), and data was analyzed by FlowJO version 10 (Tree Star, Ashland, OR). The Zombie-UV negative live single cells were gated on the basis of FITC positivity to quantify the cell types.

### Immunofluorescence microscopy

The cells growing at ALI were fixed and incubated with antibodies to occludin (Cat#sc-271,842, Santa Cruz Biotechnologies, Dallas, TX) and E-cadherin (Cat#EP700Y, Thermo Fisher Scientific, Waltham, MA), and bound antibodies were detected by using AlexaFluor 488 or 594-labelled second antibodies as previously described previously [30, 31, 34]. Briefly, cell cultures growing at ALI were washed with PBS, fixed in cold methanol for 5 min at -20° C, washed with PBS, blocked with 1% BSA for 2 h. The cell cultures were incubated with a mixture of antibodies to occludin and E-cadherin at 4° C overnight, washed to remove the unbound antibodies. The bound antibodies were detected by AlexaFluor -labeled 488 antimouse IgG and AlexaFluor -labeled 488 antirabbit IgG. The cell cultures were counter stained with DAPI and subjected to confocal microscopy. The intensity of the occludin and E-cadherin staining was quantified using NIH ImageJ software.

### Histology

For histological evaluation, cell cultures were fixed in buffered formalin, embedded in paraffin, 5µ thick sections were deparaffinized and stained with hematoxylin and eosin (H and E) or periodic acid Schiff’s (PAS) reagent.

### Real-time PCR

cDNA was synthesized from total RNA (High-capacity first strand synthesis kit, Thermofisher Scientific) and subjected to real-time qPCR to determine the expression of *ELF5*, *ELF3, GRHL1, IVL*, *WNT5, ZO-3, HOXA1, HOXB2, VGLL1, IL-8, TGF-β and glyceraldehyde 3-phosphate dehydrogenase* (*GAPDH*) using gene-specific Taqman PCR assays (ThermoFisher Scientific). The expression level of each gene is presented as a fold change over the house-keeping gene, *GAPDH*.

### Statistical analysis

Data are presented as mean ± SD or median with range. Statistical significance was assessed by t test or Mann-Whitney test to compare two groups and ANOVA with Tukey post-hoc analysis or ANOVA on Ranks with Kruskal Wallace post-hoc test to compare three groups as appropriate. For clinical samples, due to high variability in the gene expression at baseline, we compared the gene expression at base line and after completion of treatment for each patient and the statistical significance was determined by Shaipiro-Wilk T test. A p-value of ≤ 0.05 was considered statistically significant.

## Results

### Quercetin improves airway epithelial regeneration from COPD basal cells

Polarization is an important early step during the regeneration of airway epithelium from airway basal cells. Previously, we have demonstrated that compared to normal, COPD BC cultured at ALI have lower TER [16]. Therefore, we examined whether quercetin increases TER in COPD BC. In the initial experiment we determined the optimal concentration of quercetin required for improving TER and the time required for polarization of cells by using BC from one COPD patient. 90% confluent monolayer of COPD BC was treated with 0.1, 1, or 5 µM quercetin or DMSO (vehicle) for 3 days, and cells were cultured at ALI without quercetin up to 10 days. The TER was measured at 4, 6, 8 and 10 days. Normal BC cultured similarly without any treatment were used as positive control. These cells showed maximum TER on day 8 which did not change at 10 days (Supplemental Fig. 1A). As observed previously [16], untreated COPD BC showed reduced TER compared to normal BC. Treatment with DMSO or 0.1 µM quercetin had no effect on TER of COPD BC. On the other hand, cells treated with 1 µM quercetin showed increase in TER that was almost equivalent to normal BC. COPD BC treated with 5 µM quercetin showed extensive cell death at 4 days and 6 days subsequently leading to loss of cells (Supplemental Fig. 1B) and therefore the TER was not measured. Based on these results, we choose to use quercetin at 1 µM and to measure TER after 8 days of culturing the cells at ALI in the subsequent experiments.

**Fig. 1:**
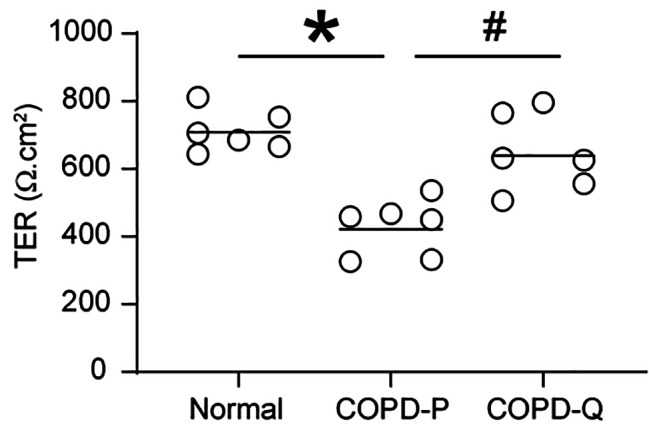
COPD BC treated with quercetin show improvement in developing intercellular junctions. A. 90% confluent monolayer of COPD BC treated with DMSO (COPD-P) or 1 µM quercetin (COPD-Q), and normal BC were cultured at air/liquid interface for 8 days. A. The TER was measured and the data was expressed as range with median (*n* = 6 (BC obtained from 6 COPD and 6 healthy non-smokers), ANOVA on ranks with Kruskal Wallace post-hoc analysis, *p ≤ 0.05, different from normal; # *p* ≤ 0.05, different from COPD-P).

**Fig. 2:**
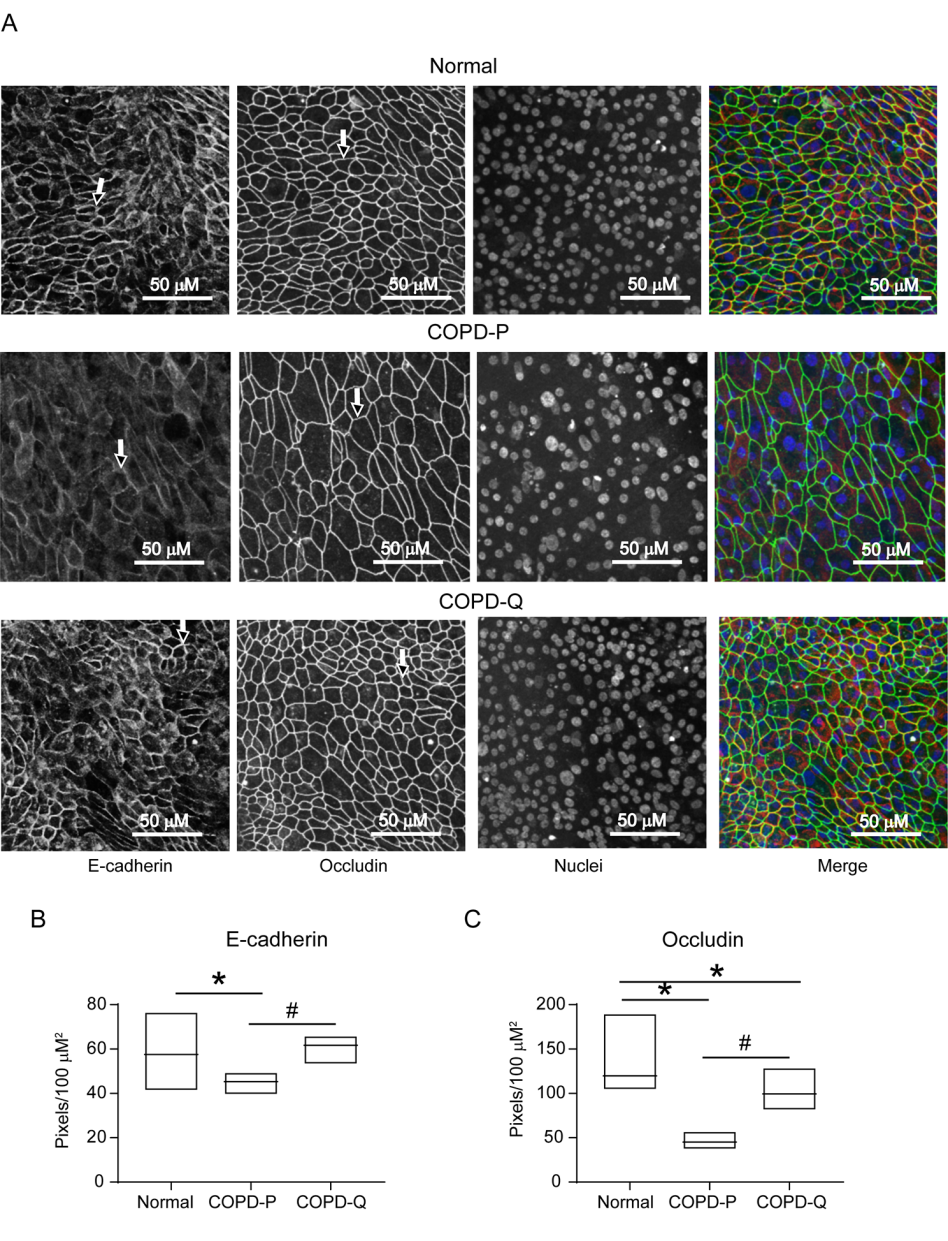
Quercetin improves expression and localization of occludin and E-cadherin to intercellular junctions in COPD cell cultures. COPD and normal cell cultures (6 subjects in each group) were cultured at air/liquid interface for 8 days as described in Fig. 1. Cultures were fixed in cold methanol, blocked with BSA and incubated with antibody to occludin and E-cadherin and the bound antibodies were detected by antimouse IgG conjugated with AlexaFluor 488 (occludin) and antirabbit IgG conjugated with AlexaFluor 594 (E-cadherin). The nuclei were counterstained with DAPI and the cells were imaged using confocal microscopy. A. Images are representative of cultures from 6 subjects. The arrows represent localization of occludin and E-cadherin. B and C. The fluorescence intensity of occludin and E-cadherin was measured as pixels from all 6 subjects and presented as median with range. (*n* = 6, ANOVA on ranks with Kruskal Wallace post-hoc analysis, *p ≤ 0.05, different from normal; # *p* ≤ 0.05, different from COPD-P).

BC from COPD patients were cultured in transwells until they reached 90% confluency and then treated with 1 µM quercetin or DMSO for 3 consecutive days. The BC were then cultured at an ALI for up to 4 weeks without quercetin to promote polarization and differentiation into mucociliary phenotype. Cell polarization was determined by measuring TER 8 days after shifting the cells to an ALI and compared with the TER of normal BC. Compared to placebo, COPD BC treated with quercetin showed significantly higher TER and was comparable to TER of healthy non-smokers’ BC (Fig. 1A). Quercetin-treated COPD cells also showed increased localization of E-cadherin and occludin to the intercellular junctions compared to placebo-treated cells (Fig. 2A). Quantitation of fluorescence intensity indicated significant improvement in the expression of occludin and E-cadherin in COPD cell cultures treated with quercetin (Fig. 2B and C).

**Fig. 3:**
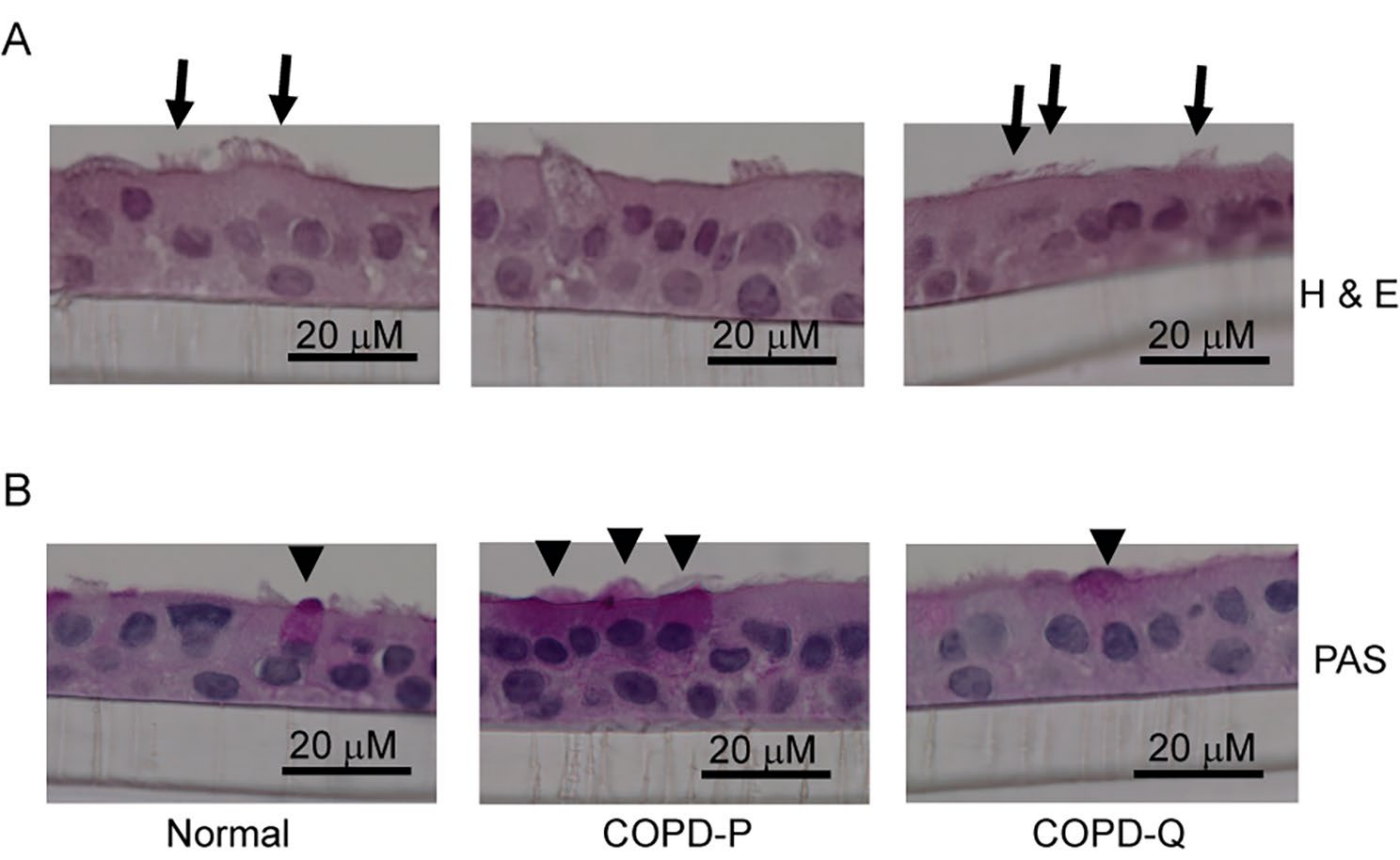
Quercetin treatment improves differentiation of COPD BC. 90% confluent monolayer of COPD BC treated with DMSO (COPD-P) or quercetin (COPD-Q), and normal BC were cultured at air/liquid interface for 4 weeks. The cultures were fixed in 10% buffered formalin, embedded in paraffin. Five-micron thick sections were deparaffinized and stained with H & E or PAS. The images are representative of cells obtained from 3 normal and 3 COPD subjects. The arrows in panel A represent ciliated cells and arrowheads in panel B represent PAS positive cells

Differentiated cell cultures from quercetin-treated BC showed more ciliated cells (arrows in Fig. 3A) and fewer PAS positive goblet cells (arrowheads in Fig. 3B) by histology. Flow cytometric analysis demonstrate that compared to normal, COPD cultures treated with placebo show less ciliated cells and more goblet and basal cells. Compared to placebo, quercetin treated COPD cell cultures show significant increase in the number of ciliated cells and decrease in goblet and basal cells (Supplemental Fig. 2 and Table 1). These results indicate that quercetin may improve airway epithelial cell polarization and differentiation.

**Table 1:** Quantitation of ciliated, goblet and basal cells in COPD cell cultures

Cell type	Normal	COPD-P	COPD-Q
Ciliated cells	44.98 ± 2.49^#^	26.49 ± 10.46*	34.53 ± 7.13^#^
Goblet cells	15.53 ± 2.84^#^	26.88 ± 6.93*	19.71 ± 4.36^#^
Basal cells	23.36 ± 2.86	33.00 ± 7.77*	28.46 ± 2.48

The data represent mean ± SD (*n* = 6 subjects in each group); *different from normal; #different from COPD-P

### Quercetin treatment reduces pro-inflammatory phenotype in COPD cell cultures

**Fig. 4:**
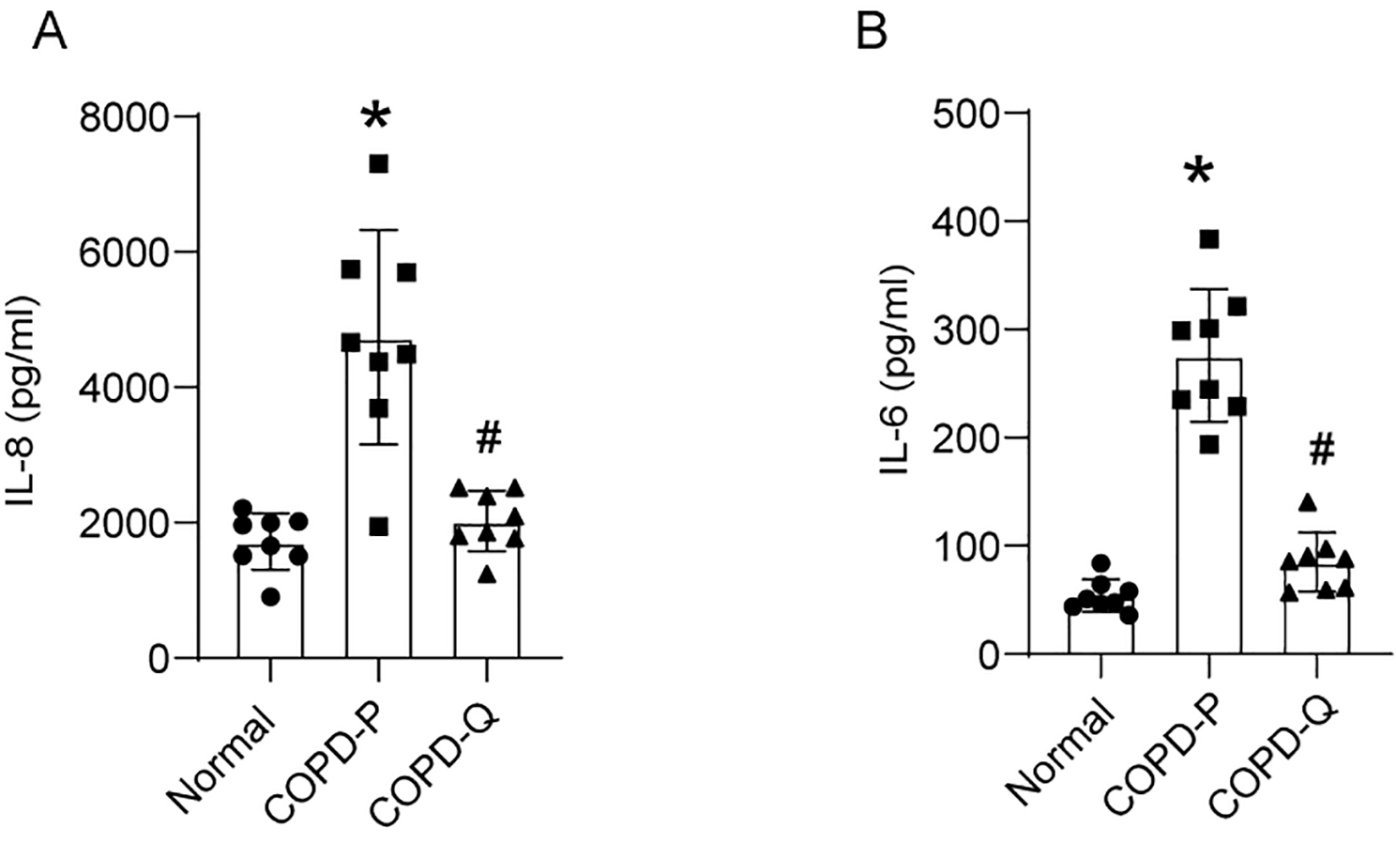
Mucociliary-differentiated cell cultures established from quercetin-treated COPD BC show attenuated levels IL-8 and IL-6. 90% confluent monolayer of COPD BC (obtained from 8 COPD subjects) treated with DMSO (COPD-P) or quercetin (COPD-Q), and normal basal cells (obtained from 8 healthy non-smokers) were cultured at air/liquid interface for 4 weeks. The transwells were transferred to new receiver plates containing fresh medium and incubated for 24 h. Basolateral medium was collected and levels of IL-8 and IL-6 were measured by ELISA. Data represent mean ± SD (*n* = 8, ANOVA with Tukey post-hoc analysis, *p ≤ 0.05, different from normal; # *p* ≤ 0.05, different from COPD-P).

Mucociliary-differentiated COPD cell cultures produced higher than normal levels of IL-8 and IL-6 proteins as observed previously [12]. The differentiated airway epithelial cell cultures generated from quercetin-, but not placebo-treated COPD BC showed reduction in the levels of both IL-6 and IL-8 (Fig. 4).

### Quercetin treatment upregulates epithelial development and differentiation genes in COPD basal cells

To identify the biological pathways that are altered by quercetin in COPD BC, we conducted microarray analysis on placebo- and quercetin-treated COPD BC from 8 donors. In a pairwise comparison (LIMMA) of placebo vs. quercetin, 1527 genes showed differential expression, 673 mapping to upregulated genes and 854 mapping to downregulated genes in quercetin-treated COPD cells (Supplemental Fig. 3 and Supplemental Table 3).

Gene ontology (GO) analysis of the upregulated genes in quercetin-treated COPD cells showed an overrepresentation of genes associated with epithelium and tissue development, and epithelial and epidermal differentiation (Table 2). Epithelial development and differentiation categories included genes encoding keratins, ELF3, ELF5, grainyhead like transcription factor (GRHL)1, GRHL3, Kruppel-like transcription factor (KLF) 4, desmoglein (DSG) 1, S100 calcium binding protein-A7 (S100-A7), bone morphogenetic protein 2, frizzled B, Wnt5A, uroplakin1A, filaggrin (FLA), involucrin and tight junction protein ZO3 (TJP3). GO analysis of down regulated genes by quercetin showed over representation of genes associated with cell cycle inhibition (Supplemental Table 4).

**Table 2:** Top 10 enriched Biological Process terms for upregulated genes in COPD-Q group

Gene Set	Description	Size	Expect	Ratio	*P* Value	FDR
GO:0008544	Epidermis development	437	11.456	4.2774	< 2.2e-16	< 2.2e-16
GO:0070268	Cornification	106	2.7787	9.3569	< 2.2e-16	< 2.2e-16
GO:0043588	Skin development	390	10.224	4.3038	2.2204e-16	6.6251e-13
GO:0030216	Keratinocyte differentiation	280	7.3400	5.0409	4.4409e-16	9.9376e-13
GO:0009913	Epidermal cell differentiation	331	8.6769	4.6100	5.5511e-16	9.9376e-13
GO:0031424	Keratinization	210	5.5050	5.6313	4.8850e-15	7.2876e-12
GO:0030855	Epithelial cell differentiation	712	18.664	3.0539	3.9524e-14	5.0540e-11
GO:0060429	Epithelium development	1166	30.566	2.2247	2.9774e-10	2.9612e-7
GO:0009888	Tissue development	1839	48.208	1.9084	4.7829e-10	4.2812e-7

### Validation of upregulated genes by quercetin in COPD cells

**Fig. 5:**
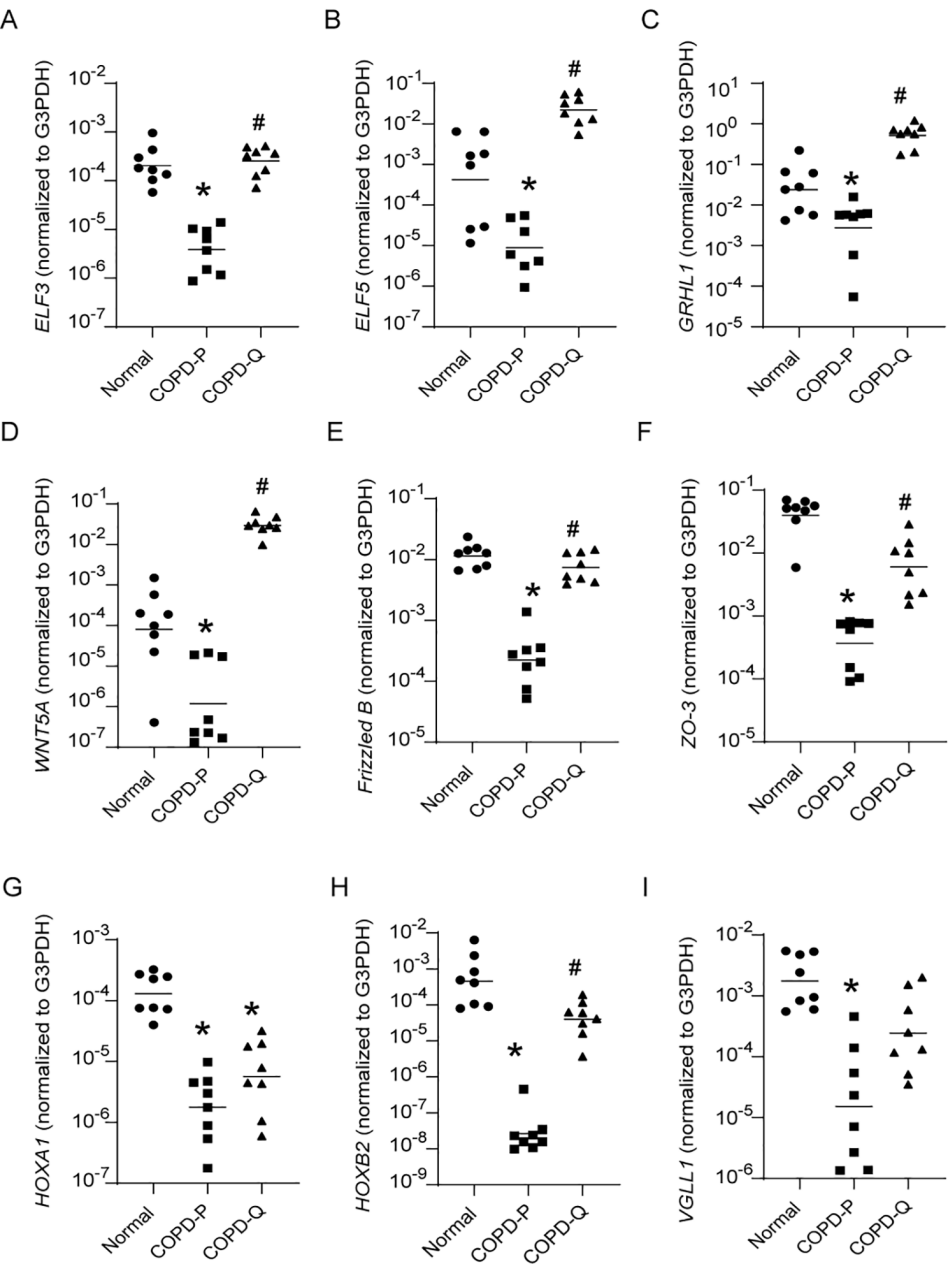
Quercetin treatment enhances expression of developmental genes. Total RNA was isolated from 90% confluent monolayer of COPD BC (obtained from 8 COPD patients) treated with DMSO (COPD-P) or quercetin (COPD-Q), and normal BC (obtained from 8 healthy non-smokers). cDNA was synthesized and mRNA expression of developmental genes, ZO-3 and G3PDH was determined by qPCR. The data was expressed as fold change over G3PDH and presented as range with median (*n* = 8, ANOVA on ranks with Kruskal Wallace post-hoc analysis, *p ≤ 0.05, different from normal; # *p* ≤ 0.05, different from COPD-P).

RT-qPCR was conducted to validate the results of microarray analysis. We measured the expression of a few of the upregulated genes involved in epithelial cell development and differentiation by Taqman probe-based PCR. Compared to normal, COPD airway basal cells showed significant reduction in the expression of ELF3, ELF5, GRHL1, WNT5A, Frizzled B and tight junction protein ZO3 (TJP3) (Fig. 5A and F). Treatment with quercetin, but not placebo increased the expression of these genes in COPD airway basal cells. We also measured the expression of HOXA1, HOXB2, VGLL1 which we had demonstrated previously to be reduced in COPD compared to normal BC (Fig. 5G and I). We compared the COPD-BC treated with placebo, COPD BC treated with quercetin and normal BC to examine whether COPD BC treated with quercetin show gene expression similar to that observed in normal BC. Compared to placebo, quercetin-treated cells showed increased expression of HOXB2, but not HOXA1 and VGLL1.

### Bronchial brushings from COPD patients treated with quercetin show increased expression of genes involved in epithelial cell development and differentiation

**Fig. 6:**
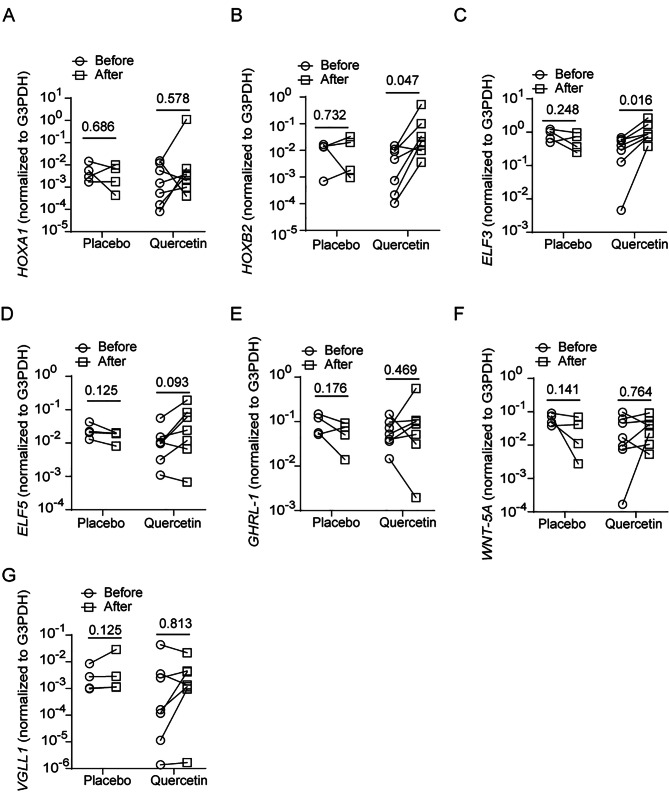
COPD patients treated with quercetin show increased expression of HOXB2 and ELF3. COPD patients were treated with placebo (4 patients) or quercetin (7 patients) for 6 months. Total RNA isolated from bronchial brushings obtained before and after completion of treatment was subjected RT-qPCR with gene-specific Taqman assays. The data was expressed as fold increase over G3PDH and represents intra-comparison of gene expression before and after treatment with placebo or quercetin (#*p* ≤ 0.05, different from sham, paired Shipiro-Wilk T test)

**Fig. 7:**
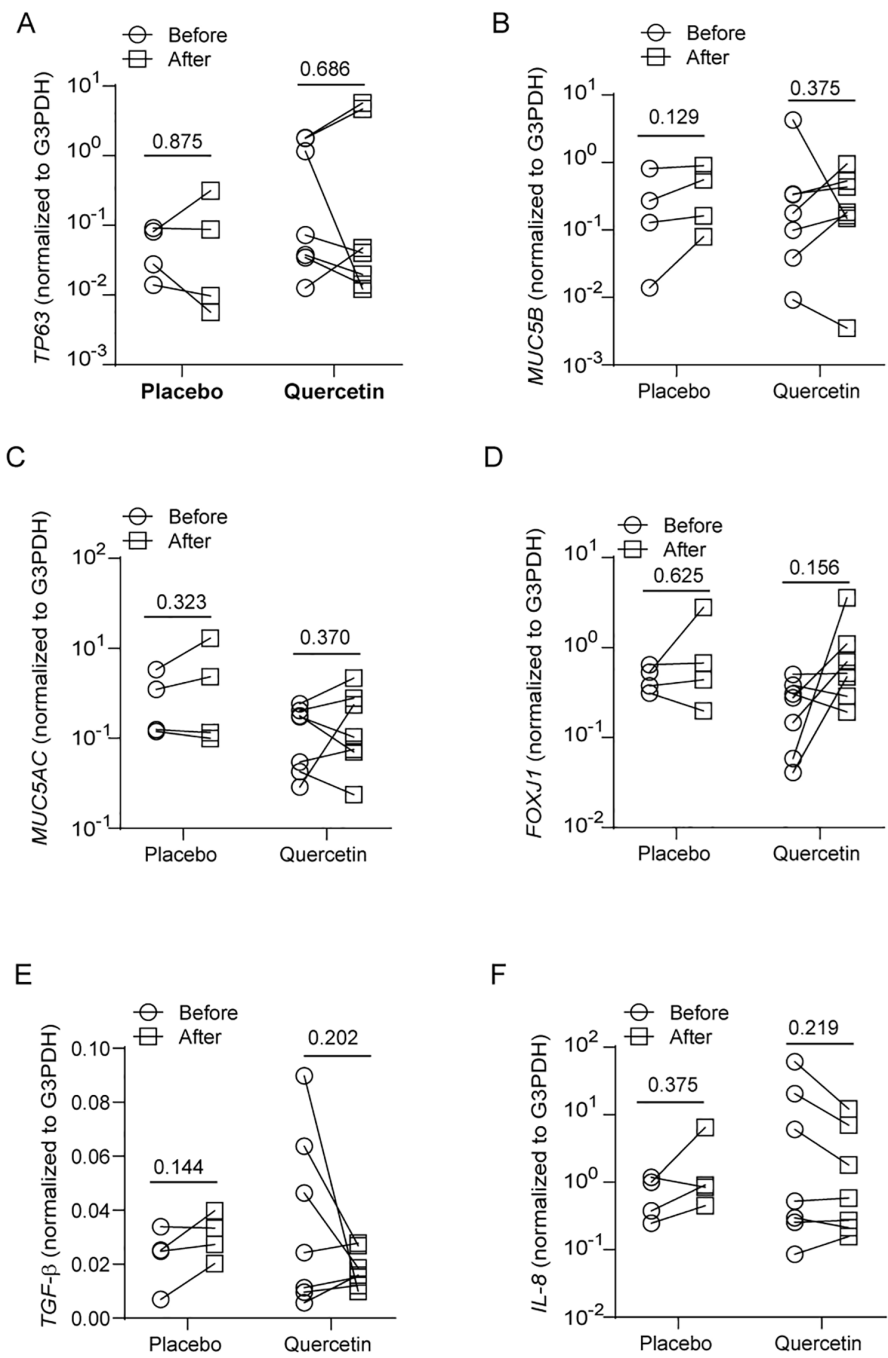
Effect of quercetin on the markers of different cell types and cytokines. COPD patients were treated with placebo (4 patients) or quercetin (7 patients) for 6 months. Total RNA isolated from bronchial brushings obtained before and after completion of treatment was subjected RT-qPCR using Taqman probe-based PCR assays. The data was expressed as fold increase over G3PDH and represents intra-comparison of gene expression before and after treatment with placebo or quercetin. Statistical significance was analyzed by paired Shipiro-Wilk T test

Recently, we completed a small placebo-controlled clinical trial with quercetin in COPD patients. In this clinical trial, we had 4 patients treated with placebo and 7 with 2000 mg/day quercetin for 6 months. There was no significant difference in the age, FEV1% predicted and FEV1/FVC at baseline between placebo and quercetin groups (Supplemental Table 2). Plasma quercetin levels at baseline ranged between 0.33 and 0.63 µM with an average of 0.467 ± 0.104 µM with no significant difference between placebo and quercetin-treated patients. Patients in both groups showed 70 to 100% compliance. Quercetin, but not placebo-treated patients showed 1.4 to 4.9 folds increase in plasma quercetin levels over their respective baseline at the end of treatment period. FEV1% predicted did not change significantly in either placebo or quercetin group. From this clinical trial, we had access to bronchial brushings collected at baseline and at the end of treatment. Total RNA was isolated from the bronchial brushings and analyzed for the expression of HOXA1, HOXB2, VGLL1, ELF3, ELF5, GRHL1, and WNT5A, and also markers of different cell types of airway epithelium, such as TP63 (basal cell marker), FOXJ1 (ciliated cell marker), and MUC5B and MUC5AC (goblet cell markers). Out of these genes, the expression of only HOXB2 and ELF3 showed a significant increase in quercetin-, but not in placebo-treated patients (Fig. 6). Three out of 7 quercetin-treated patients also showed an increase in HOXA1 and VGLL1 expression. There was no alteration in the expression of mucin genes or basal cell marker TP63 in both placebo- and quercetin-treated patients (Fig. 7A and C). The expression of FOXJ1 increased in 4 out of 7 quercetin-treated patients and one out of 4 placebo-treated patients (Fig. 7D).

Next, we examined the expression of TGF-β, which contributes to persistent epithelial to mesenchymal transition and abnormal repair in COPD [14], and the expression of pro-inflammatory cytokines, IL-6 and IL-8. Placebo-treated patients showed no alteration in the expression of either TGF-β or IL-8. IL-6 was below the detection limit in both placebo-and quercetin-treated patients. Interestingly, 3 out of 7 quercetin-treated patients showed a reduction in the mRNA expression of TGF-β and IL-8 (Fig. 7E and F). Together, these results indicate that quercetin may promote normal repair/regeneration of airway epithelium in COPD by inducing the expression of developmental genes.

## Discussion

The present study shows that treatment of COPD BC with quercetin corrects defects in airway epithelial regeneration and this was associated with upregulation in the expression of several developmental genes that may participate in epidermal and epithelial differentiation. Interestingly, we also show that two of the developmental genes, HOXB2 and ELF3 which increased in COPD BC after treatment with quercetin in vitro, were also significantly increased in the bronchial brushings of COPD patients treated with 2000 mg/day quercetin for 6 months.

Human BC from tracheobronchial tissue regenerate epithelium that resembles airway epithelium in vivo. The BC express several developmental genes including ELF3, ELF5, WNT5A, NOTCH1-4, GRHL1, GRHL2 and VGLL1. Out of these genes, WNT, NOTCH, GRHL2 and ELF3 have been demonstrated to play a role in bronchial epithelial regeneration and maintaining homeostasis [35–38]. COPD BC regenerate abnormal airway epithelium showing goblet cell hyperplasia and pro-inflammatory type [12, 39]. This was associated with reduced expression of several of these developmental genes and also previously unidentified developmental genes HOXA1 and HOXB2 [16]. In the present study, we found that treatment of COPD BC with 1µM quercetin for 3 days significantly increases the expression of developmental genes including HOXB2, ELF3, ELF5, GRHL1, GRHL2, and WNT5A in these cells and this was associated with improved polarization and differentiation.

Previously, we have shown that knockdown of HOXB2 contributes to polarization of airway epithelial cells [16]. Previous research demonstrates that knockdown of ELF3 resulted in delayed repair of bronchiolar epithelium following injury in mice and also a reduction in the expression of TGF-β type II receptor, which is involved in epithelial cell differentiation indicating ELF3 may be required for both airway basal cell proliferation and differentiation [35]. Genetic inhibition of GRHL2 in airway epithelial cells reduced the expression of E-cadherin and claudin 4. Therefore, it is possible that GRHL2 may play a role in the development of tight junctions [40]. Canonical activation of WNT/β-catenin signaling not only maintains the stemness of the basal cells during the proliferation of BC but also cilia formation in multiciliated cells during differentiation [37]. Based on this literature, it is reasonable to presume that several developmental genes function in a coordinated fashion to regenerate airway epithelium. Therefore, it is plausible that quercetin-induced expression of several developmental genes may function in coordinated fashion to improve airway epithelial regeneration in COPD BC. The mechanism by which quercetin alters gene expression in COPD BC is not known at present. But given the capacity of quercetin to modulate epigenetic changes [41], we speculate that quercetin may alter the expression of developmental genes by erasing the acquired epigenetic changes in COPD BC, which will be explored in the future.

Interestingly, we also noted negative regulation of genes that are associated with cell cycle and proliferation in quercetin-treated COPD BC. Inhibition of cell cycle and proliferation may be necessary during the repair process after the cells reach confluency (which represent wound closure following injury) to promote polarization and differentiation. If the proliferation of BC continues after the wound closure, it can lead to basal cell hyperplasia, which is often observed in patients with COPD [10]. In addition, it may also lead to persistent EMT and abnormal differentiation resulting in goblet cell and squamous metaplasia. Based on these observations, quercetin-promoted normal airway epithelial regeneration may depend on both increased expression of developmental genes and inhibition of cell cycle genes after the cells reach 90 to 100% confluency.

Intriguingly, we observed that treatment with quercetin, but not placebo for 6 months significantly upregulated the expression of HOXB2 and ELF3 in the bronchial epithelial cells of COPD patients. We did not observe significant changes in other developmental genes examined and this may be attributed to presence of other cell types such as ciliated cells, goblet cells in addition to BC in bronchial brushings. Some of the developmental genes may be specific to BC and presence of other cell types can mask the effects of quercetin. Since HOXB2 and ELF3 expression is increased both in vitro and in vivo, their role in airway epithelial repair/regeneration in COPD will be explored in the future.

Four out of 7 quercetin-treated patients also showed increased expression of ciliated cell marker, FOXJ1. Interestingly, three of these patients showed 65 to 80% and 60 to 90% reduction in the expression of TGF-β and IL-8 respectively. All these three patients are current smokers and show higher basal levels of TGF-β and IL-8. These results may indicate that active smokers may have increased expression of pro-inflammatory cytokines and less ciliated cells, which may be corrected by quercetin treatment. Previous research has shown that cigarette smoke enhances TGF-β expression, which drives epithelial to mesenchymal transition [14]. Sustained epithelial to mesenchymal transition impair regeneration of airway epithelium and enhance expression of pro-inflammatory cytokines [42]. HOXB2 which promotes polarization [43] and ELF3, which represses epithelial to mesenchymal transition and participates in bronchial epithelial repair respectively [35, 44] together may improve polarization and repair of bronchial epithelium in quercetin-treated COPD patients. This in turn may promote normal differentiation of cells subsequently reducing the expression of TGF-β and IL-8.

The plasma quercetin levels in COPD patients after treatment with quercetin for 6 months varied from one patient to another and was increased by 1.4 to 4.9 folds. This may be due to variable compliance or variability in absorption of quercetin. However, there was no correlation between plasma quercetin levels and gene expression. This may be because the plasma quercetin levels may not represent levels or distribution of quercetin in the lungs.

There are some limitations with this study. The COPD BC used for microarray analysis are from patients with end-stage disease undergoing lung transplantation, who have ceased smoking for at least six months prior to collection of the airway tissue. Therefore, we expect that BC from COPD patients with mild to moderate lung disease and active smokers may show variable changes in the expression of these developmental genes, which will be a subject for future investigation. Second, in the clinical trial there were very few patients in placebo and quercetin group. Third, we utilized bronchial brushings which will have a mixture of ciliated, goblet and basal cells along with other minor cell types of bronchial epithelium to determine the quercetin-induced expression of developmental genes due to the availability of limited sample. Four, we could not determine the level or distribution of quercetin in the lungs, which may be necessary to correlate the changes in the gene expression in bronchial brushings. Despite these limitations, we observed significant increases in HOXB2 and ELF3 in quercetin-treated patients, which were also increased in COPD BC treated with quercetin in vitro. There was also indication that current COPD smokers may have increased expression of pro-inflammatory cytokines, which may be reduced by quercetin treatment. A larger clinical trial with quercetin in COPD patients is necessary to confirm the findings of this small study and also to understand the effects of quercetin in-depth in COPD population.

In summary, our results suggest that quercetin may promote normal airway epithelium regeneration from COPD BC by modulating the expression of developmental genes, particularly, HOXB2 and ELF3. In the future studies, we will focus on the elucidating the mechanisms by which these two developmental genes coordinate to promote polarization and differentiation of airway epithelium in COPD.

### Electronic supplementary material

Below is the link to the electronic supplementary material.

Supplementary Material 1

## Data Availability

All the data are available are presented in the manuscript. The microarray data has been deposited into public domain in Gene Expression Omnibus (GEO) with accession number GSE137557.
