# Supplementary material for: Quercetin improves epithelial regeneration from airway basal cells of COPD patients
Source: Respir Res. 2024 Mar 11;25:120. doi: 10.1186/s12931-024-02742-0 (PMC10926630; doi:10.1186/s12931-024-02742-0)
Supplement: Supplementary file 1 — Supplementary Material 1 [file 12931_2024_2742_MOESM1_ESM.pdf]

Supplemental data

Quercetin improves epithelial regeneration from airway basal cells of COPD patients

Elizabeth S. McCluskey[1], Nathan Liu[1], Abhimaneu Pandey[1], Nathaniel Marchetti[3], Steven G Kelsen[3] and Umadevi S. Sajjan[1,2,3]

Supplemental Figure Legends

Supplemental Figure 1. Optimization of quercetin dose and time of culture at ALI for TER measurements. Ninety % confluent BC from one COPD patient were treated with vehicle or 01, 1 and 5 μM quercetin for 3 days. The cells were then cultured at ALI up to 10 days and TER was monitored on days 4, 6, 8 and 10. BC from one healthy non-smoker was cultured similarly without treatment with DMSO or quercetin. The experiment was repeated three times using P1 cells. For each experiment, cells at P0 were thawed grown to 80% confluence on 10 cm dish, dissociated from the plate and seeded in transwells. The data represent mean ± SEM calculated from 3 independent experiments and the statistical significance was determined by ANOVA with Tukey post-hoc analysis (*$p \leq 0.05$, different from normal; # $p \leq 0.05$, different from COPD cells treated with 1 μM quercetin).

Supplemental Figure 2. Detection of Ciliated, goblet and basal cells in mucociliary-differentiated normal and COPD cultures. Basal cells obtained 6 COPD patients were grown to 90% confluency and treated with DMSO or 1 μM quercetin for three days. The cells were then cultured at ALI for 4 weeks to promote mucociliary differentiation. BC obtained from 6 healthy non-smokers cultured at ALI for 4 weeks were used as controls. The cells were dissociated from the transwells, stained with Zombie to detect dead cells, fixed/permeabilized, blocked with 5% BSA and incubated with antibodies to acetylated tubulin (ciliated cells), MUC5AC (goblet cells) or TP63 (basal cells) or normal mouse IgG (control to detect FITC positive cells). Cells were then washed to remove unbound antibodies and then incubated with antimouse IgG labeled with FITC and analyzed by flow cytometry. The cells were selected on the basis of forward and side scatter, dead cells were gated out based on Zombie staining and live cells were gated on the basis of FITC positivity. The data in Panel A shows gating strategy used for cells incubated with

normal mouse IgG (negative control). Panel B to D shows live cells gated on the basis of FITC positivity.

Supplemental Figure 3. Differential expression of genes by microarray in COPD BC treated with quercetin. COPD BC were treated with placebo or quercetin for 3 consecutive days, total RNA was isolated and subjected to microarray analysis using Human Gene 2.1 ST arrays. The data was analyzed by TAC software analysis to identify the number of altered genes at least by 2 fold with FDR p value <0.05.

Table 1. Study population

Subject	Age	Sex	Pack-years	FEV1% predicted
**Normal (8)**	**50**	**4:4**		
N4	59	F	-	-
N17	33	M	-	-
N18	16	M	-	-
N19	50	F	-	-
N20	52	M	-	-
N22	62	M	-	-
N24	50	F	-	-
N27	48	F	-	-
**COPD (8)**	**54**	**5:3**	**41.25**	**17**
C6	49	F	25	17
C10	53	F	37.5	14
C11	59	M	70	22
C12	49	F	136	13
C14	55	M	25	17
C15	45	M	31.25	17
C18	67	M	90	20
C19	64	M	45	29

Supplemental Table 2: Characteristics of patients treated with Placebo or Quercetin

Characteristics	Placebo group	Quercetin group	P Value
Age yr, mean ± SD	67.7 (± 4.99)	67.4 (± 63.9)	0.201
Total (Males)	4 (2)	7 (3)	0.331
FEV1 (% of predicted)	57.5 (± 10.24)	53.7 (± 12.29)	0.476
FEV1/FVC	0.61 (± 0.11)	0.55 (± 0.08)	0.345

Supplemental Table 4. Top 10 enriched Biological Process terms for downregulated genes in COPD-Q group

Gene Set	Description	Size	Expect	Ratio	P Value	FDR
GO:0007049	Cell cycle	1661	41.123	4.0124	<2.2e-16	<2.2e-16
GO:0022402	Cell cycle process	1219	2.7787	9.3569	<2.2e-16	<2.2e-16
GO:0051726	Regulation of cell cycle	1050	10.224	4.3038	<2.2e-16	<2.2e-16
GO:0006259	DNA metabolic process	932	7.3400	5.0409	<2.2e-16	<2.2e-16
GO:0000278	Mitotic cell cycle	885	8.6769	4.6100	<2.2e-16	<2.2e-16
GO:0006974	Cellular response to DNA damage stimulus	767	5.5050	5.6313	<2.2e-16	<2.2e-16
GO:0007346	Regulation of mitotic cell cycle	552	18.664	3.0539	<2.2e-16	<2.2e-16
GO:0045786	Negative regulation of cell cycle	537	30.566	2.2247	<2.2e-16	<2.2e-16
GO:0006281	DNA repair	489	48.208	1.9084	<2.2e-16	<2.2e-16
GO:0006260	DNA replication	259	6.4123	8.7333	<2.2e-16	<2.2e-16

Supplemental Table3. CP vs CQ pairwise comparison									
**ID**	**C_Q Avg (l**	**C Avg (log**	**C_Q Stand**	**C Standard**	**Fold Chan**	**P-val**	**FDR P-val**	**Gene Symbol**	**Description**
16872611	9.05	1.42	0.93	1.06	197.91	1.03E-11	4.52E-08	CEACAM7	carcinoembryonic antigen-related cell adhesion molecule 7
16949322	9	1.93	1.78	1.26	135	6.20E-08	8.80E-06	FETUB	fetuin B
16872777	11.18	4.39	0.86	1.23	110.52	9.69E-11	1.90E-07	CNFN	cornifelin
16910766	10.4	3.83	1.69	1.68	94.73	3.06E-07	2.59E-05	TGM3	transglutaminase 3
16693323	10.84	4.46	0.98	1.43	83.31	3.39E-09	1.27E-06	CRNN	cornulin
17122142	9.6	3.63	3.13	1.94	62.67	0.0009	0.0104		
16840799	8.22	2.38	0.76	1.3	57.12	9.45E-10	6.24E-07	ALOX12B	arachidonate 12-lipoxygenase, 12R type
17025738	7.09	1.32	1.5	0.71	54.62	2.07E-08	4.23E-06	LOC441178; LOC1	uncharacterized LOC441178; uncharacterized LOC105378132
16924785	6.96	1.23	1.28	0.52	53.32	4.91E-10	4.02E-07	CLDN17	claudin 17
17081945	8.97	3.24	1.36	0.95	53.05	1.26E-08	3.15E-06	SLURP1	secreted LY6/PLAUR domain containing 1
16882834	9.51	3.79	1.09	0.9	52.8	3.70E-10	3.52E-07	MAL	mal, T-cell differentiation protein
16976488	8.9	3.36	1.63	0.75	46.6	4.43E-08	7.08E-06	TMPRSS11B	transmembrane protease, serine 11B
16874751	8.06	3	1.5	0.96	33.35	6.29E-08	8.90E-06	KLK12	kallikrein related peptidase 12
16851708	7.58	2.61	0.94	1.81	31.51	4.34E-07	3.28E-05	DSG1	desmoglein 1
16868453	10.17	5.35	1.07	1.08	28.27	4.64E-08	7.36E-06	ZNF812P	zinc finger protein 812, pseudogene
16852849	7.51	2.82	0.73	1.42	25.75	9.40E-08	1.15E-05	SERPINB11	serpin peptidase inhibitor, clade B (ovalbumin), member 11 (gene/pseudogene)
16773260	9.77	5.09	0.97	1.25	25.62	3.12E-08	5.47E-06	ATP12A	ATPase, H+/K+ transporting, nongastric, alpha polypeptide
16990787	9.69	5.02	0.87	1.39	25.46	4.10E-08	6.73E-06	SPINK7	serine peptidase inhibitor, Kazal type 7 (putative)
17038014	8.46	3.9	1.11	1.61	23.54	2.61E-06	0.0001		
16884602	5.81	1.31	1.6	0.32	22.67	4.00E-08	6.58E-06	IL36A	interleukin 36, alpha
17033237	7.64	3.19	1.16	1.36	21.9	1.12E-06	6.37E-05		
16699560	7.78	3.33	1.13	1.24	21.81	7.60E-07	4.84E-05		
16872978	5.91	1.46	1.58	0.52	21.73	5.32E-07	3.72E-05		
16871349	7.32	2.89	0.89	0.89	21.59	1.73E-09	8.59E-07	KRTDAP	keratinocyte differentiation-associated protein
16844477	9.55	5.13	0.8	1.42	21.44	9.54E-08	1.16E-05	KRT10	keratin 10, type I
17006532	7.7	3.32	1.09	1.42	20.86	1.53E-06	7.96E-05	MUC21	mucin 21, cell surface associated
17026232	7.7	3.32	1.09	1.42	20.86	1.53E-06	7.96E-05		
17030532	7.7	3.32	1.09	1.42	20.86	1.53E-06	7.96E-05		
16871403	9.18	4.8	0.68	1.3	20.77	2.14E-08	4.30E-06	SBSN	suprabasin
17035325	7.91	3.57	1.08	1.38	20.29	1.57E-06	8.08E-05		
16862790	7.17	2.88	1.72	0.68	19.64	3.13E-06	0.0001	CD177	CD177 molecule
16914171	11.57	7.33	0.29	1.1	18.97	3.55E-09	1.29E-06	PI3	peptidase inhibitor 3, skin-derived
17014712	7.54	3.37	1.08	1.02	18.05	4.17E-07	3.19E-05		
17002429	7.91	3.84	0.68	1.26	16.79	1.50E-07	1.58E-05	ATP10B	ATPase, class V, type 10B
17123812	5.4	1.34	1.39	0.47	16.67	4.66E-07	3.42E-05		
16761549	5.97	2.04	1.57	0.63	15.27	1.36E-06	7.29E-05	PRB1	proline-rich protein BstNI subfamily 1
16976455	6.35	2.45	1.05	0.93	14.96	2.67E-07	2.36E-05	TMPRSS11A	transmembrane protease, serine 11A
17001349	4.95	1.06	1.55	0.13	14.87	3.77E-06	0.0002	SPINK1	serine peptidase inhibitor, Kazal type 1
16852824	6.7	2.83	0.86	1.05	14.68	1.33E-07	1.46E-05	SERPINB12	serpin peptidase inhibitor, clade B (ovalbumin), member 12
17123802	5.35	1.49	1.36	0.45	14.58	5.96E-07	4.01E-05		
16830238	5	1.15	1.66	0.32	14.45	3.21E-06	0.0001	ALOX12	arachidonate 12-lipoxygenase
17081951	5.84	2.03	1.47	0.2	14	1.06E-06	6.14E-05	LYPD2	LY6/PLAUR domain containing 2
16765080	10.15	6.35	0.85	1.09	13.93	3.17E-07	2.64E-05	KRT4	keratin 4, type II
16793299	7.81	4.04	0.85	0.63	13.65	1.43E-08	3.32E-06	LINC00520	long intergenic non-protein coding RNA 520
17123836	5.47	1.74	1.18	1.1	13.26	2.85E-06	0.0001		
16699550	4.23	0.61	0.74	0.37	12.31	1.44E-10	2.25E-07		
16699552	4.96	1.39	0.84	0.44	11.88	3.05E-09	1.20E-06	LOC105372950	uncharacterized LOC105372950
16705216	6.38	2.82	0.61	0.77	11.76	1.06E-09	6.79E-07	LOC105378325	uncharacterized LOC105378325
16765116	5.04	1.52	1.45	0.37	11.52	1.65E-06	8.41E-05	KRT78	keratin 78, type II
16955580	6.21	2.7	0.95	0.82	11.41	1.53E-07	1.59E-05	FAM3D	family with sequence similarity 3, member D
16693427	5.68	2.2	0.72	0.84	11.14	5.23E-08	7.86E-06	S100A7	S100 calcium binding protein A7
16874798	8.1	4.62	0.6	0.9	11.1	1.44E-08	3.33E-06	VSIG10L	V-set and immunoglobulin domain containing 10 like
16976483	5.46	2.02	1.01	0.8	10.9	5.76E-07	3.90E-05	TMPRSS11BNL	TMPRSS11B N-terminal like, pseudogene
17109142	4.6	1.17	1.73	0.47	10.82	0.0001	0.0021		
16693383	6.61	3.18	0.67	0.92	10.77	2.29E-08	4.38E-06	PGLYRP3	peptidoglycan recognition protein 3
16761693	7.37	3.97	1.39	1.36	10.61	7.65E-05	0.0015	LINC01559	long intergenic non-protein coding RNA 1559
16990321	4.69	1.33	0.82	0.88	10.28	3.53E-07	2.86E-05	PCDHB14	protocadherin beta 14
16716507	7.43	4.09	1.99	1.19	10.08	0.0002	0.0033	PPP1R3C	protein phosphatase 1, regulatory subunit 3C
16707096	4.52	1.21	1.01	0.34	9.98	5.50E-08	8.13E-06	LIPN	lipase, family member N
16730157	4.98	1.67	0.65	0.63	9.89	3.40E-09	1.27E-06	HEPHL1	hephaestin-like 1
16671023	8.43	5.14	0.73	1.1	9.81	4.00E-07	3.11E-05	CRCT1	cysteine rich C-terminal 1
16693295	9.17	5.89	0.48	1.88	9.7	1.23E-05	0.0004	RPTN	repetin
17087879	4.22	0.96	1.31	0.2	9.57	5.02E-06	0.0002		
16872621	7.38	4.25	0.45	0.81	8.77	5.12E-09	1.65E-06		
17123540	5.16	2.05	1.25	0.51	8.66	4.59E-06	0.0002		
16824352	4.95	1.89	1.39	0.51	8.34	1.59E-05	0.0005	XYLT1	xylosyltransferase I
16693335	5.12	2.16	1.25	0.45	7.76	1.87E-07	1.83E-05	LCE3D	late cornified envelope 3D
16813112	9.18	6.23	0.51	0.92	7.75	7.27E-08	9.74E-06	RHCG	Rh family, C glycoprotein
16976177	4.57	1.62	1.05	0.21	7.73	5.33E-07	3.72E-05	HOPX	HOP homeobox
16761726	6.63	3.68	0.74	0.71	7.69	1.07E-07	1.24E-05	PLBD1	phospholipase B domain containing 1
16925872	4.85	1.91	1.01	0.42	7.67	2.46E-07	2.22E-05	TMPRSS2	transmembrane protease, serine 2
16707729	8.35	5.41	0.68	1.46	7.66	2.29E-05	0.0006	CYP2C18	cytochrome P450, family 2, subfamily C, polypeptide 18
16884372	5.22	2.3	1.04	0.42	7.58	2.64E-06	0.0001	MERTK	MER proto-oncogene, tyrosine kinase
17061129	5.96	3.04	0.95	1.62	7.55	0.0011	0.0124	RASA4B; RASA4	RAS p21 protein activator 4B; RAS p21 protein activator 4

16671094	6.66	3.77	0.55	1.05	7.44	2.40E-06	0.0001	IVL	involucrin
17081297	5.31	2.46	0.85	0.38	7.2	1.96E-07	1.89E-05		
16896170	6.7	3.86	0.92	0.89	7.13	2.44E-06	0.0001	CAPN14	calpain 14
17094917	6.98	4.15	1.18	1.34	7.1	0.0002	0.0026	LOC105376081	uncharacterized LOC105376081
16746217	4.03	1.21	1.22	0.54	7.06	1.72E-05	0.0005		
16962799	6.65	3.84	0.78	1.12	7.03	2.90E-06	0.0001	ATP13A4	ATPase type 13A4
16869803	7.41	4.6	0.64	0.96	7.01	1.40E-06	7.44E-05	EPHX3	epoxide hydrolase 3
17011279	7.12	4.31	0.41	0.26	7.01	2.85E-12	1.53E-08	PRDM1	PR domain containing 1, with ZNF domain
17095345	5.51	2.71	0.76	0.83	6.97	4.61E-07	3.40E-05	SLC28A3	solute carrier family 28 (concentrative nucleoside transporter), member 3
16861945	10.24	7.45	0.56	0.78	6.93	1.20E-07	1.35E-05	NCCRP1	non-specific cytotoxic cell receptor protein 1 homolog (zebrafish)
16766334	4.66	1.88	0.98	1.01	6.85	2.30E-05	0.0006	SDR9C7	short chain dehydrogenase/reductase family 9C, member 7
16976438	8.37	5.59	0.59	0.53	6.83	1.45E-08	3.34E-06	TMPRSS11D	transmembrane protease, serine 11D
16967327	8.33	5.57	0.82	0.86	6.77	2.58E-06	0.0001	TMPRSS11E	transmembrane protease, serine 11E
16980021	6.08	3.34	1.02	0.87	6.68	1.49E-05	0.0004	SETD7	SET domain containing (lysine methyltransferase) 7
17096030	5.4	2.67	1.58	0.46	6.64	0.0004	0.0056	FBP1	fructose-1,6-bisphosphatase 1
17087874	3.72	1.03	1.26	0.33	6.48	4.58E-05	0.001		
17122140	5.32	2.62	1.49	0.2	6.47	5.73E-05	0.0012		
16703659	4.65	1.95	0.61	0.69	6.46	5.66E-08	8.31E-06	MAP3K8	mitogen-activated protein kinase kinase kinase 8
17010544	9.39	6.7	0.69	0.56	6.46	7.69E-08	9.96E-06	SH3BGRL2	SH3 domain binding glutamate-rich protein like 2
16865590	6.95	4.28	0.64	0.41	6.37	2.92E-09	1.17E-06	EPS8L1	EPS8-like 1
16867558	4.11	1.45	0.85	0.47	6.3	3.08E-07	2.60E-05	FUT6	fucosyltransferase 6 (alpha (1,3) fucosyltransferase)
16808066	5.5	2.84	0.63	1.35	6.29	9.76E-06	0.0003	TGM5	transglutaminase 5
16660014	5.91	3.26	0.91	0.74	6.29	2.46E-06	0.0001	PADI1	peptidyl arginine deiminase, type I
16981791	10.29	7.65	0.8	0.75	6.24	5.22E-07	3.68E-05		
16914935	3.13	0.49	1.29	0.98	6.24	0.0024	0.0228		
16977502	6.87	4.24	1.03	0.6	6.22	1.32E-05	0.0004	PLAC8	placenta specific 8
16712373	5.92	3.32	0.64	0.87	6.08	1.61E-06	8.27E-05	NEBL	nebulette
16764907	3.16	0.58	1.27	0.78	5.98	0.0003	0.0042	KRT6C	keratin 6C, type II
17123808	3.92	1.35	1.25	0.36	5.93	1.42E-05	0.0004		
16844735	10.31	7.75	0.98	1.61	5.87	0.0008	0.0099	KRT13	keratin 13, type I
16769481	6.82	4.27	0.54	0.67	5.87	7.26E-08	9.74E-06	ALDH1L2	aldehyde dehydrogenase 1 family, member L2
16974049	4.32	1.77	0.27	0.27	5.86	2.68E-13	3.95E-09	PPP2R2C	protein phosphatase 2, regulatory subunit B, gamma
16962850	5.6	3.08	0.93	0.69	5.75	1.72E-06	8.67E-05	LOC105374284	uncharacterized LOC105374284
16703563	5.65	3.19	1.02	0.84	5.49	4.73E-05	0.001	BAMBI	BMP and activin membrane-bound inhibitor
16731490	4.07	1.62	1.15	0.59	5.46	6.97E-05	0.0014	LOC105369502	uncharacterized LOC105369502
17094893	5.78	3.34	0.83	0.89	5.44	1.15E-05	0.0004	ALDH1A1	aldehyde dehydrogenase 1 family, member A1
16877097	4.27	1.83	0.62	0.59	5.44	4.46E-07	3.34E-05	ATP6V1C2	ATPase, H+ transporting, lysosomal 42kDa, V1 subunit C2
16756310	6.01	3.57	0.46	0.31	5.41	1.12E-10	1.93E-07	TCP11L2	t-complex 11, testis-specific-like 2
17044253	6.45	4.03	1.14	1.27	5.36	0.0005	0.0068	GPNMB	glycoprotein (transmembrane) nmb
17061097	5.26	2.85	0.65	0.98	5.32	2.60E-05	0.0007	RASA4B; RASA4	RAS p21 protein activator 4B; RAS p21 protein activator 4
16737282	6.65	4.24	0.64	0.64	5.31	1.07E-07	1.24E-05	ELF5	E74-like factor 5 (ets domain transcription factor)
16716525	5.06	2.66	0.52	0.48	5.26	1.43E-08	3.32E-06	CPEB3	cytoplasmic polyadenylation element binding protein 3
17059567	6.68	4.29	0.87	0.55	5.24	7.28E-07	4.70E-05	STEAP4	STEAP family member 4
16829801	4.54	2.15	0.79	0.62	5.22	1.53E-07	1.59E-05	SPNS2	spinster homolog 2 (Drosophila)
16955220	3.38	1	1.33	0.29	5.21	0.0001	0.0021	WNT5A	wingless-type MMTV integration site family, member 5A
16888578	7.17	4.8	0.66	0.32	5.19	2.86E-08	5.11E-06	GULP1	GULP, engulfment adaptor PTB domain containing 1
16968274	3.21	0.84	1.12	0.25	5.17	4.84E-06	0.0002	LINC01088	long intergenic non-protein coding RNA 1088
16764791	6.15	3.8	0.82	0.35	5.12	7.84E-07	4.96E-05	KRT80	keratin 80, type II
16846218	4.96	2.63	0.74	0.63	5	1.12E-06	6.37E-05	HOXB2	homeobox B2
17061119	4.32	2.02	1.06	1.32	4.94	0.0026	0.0241	RASA4B; RASA4	RAS p21 protein activator 4B; RAS p21 protein activator 4
17105903	3.21	0.92	1.16	0.2	4.91	3.40E-06	0.0001	MUM1L1	melanoma associated antigen (mutated) 1-like 1
16862548	11.53	9.24	0.28	1.01	4.9	9.02E-07	5.52E-05	CEACAM5	carcinoembryonic antigen-related cell adhesion molecule 5
16816978	7.15	4.86	0.62	0.81	4.9	5.58E-07	3.84E-05	SCNN1B	sodium channel, non voltage gated 1 beta subunit
16861126	4.27	1.98	1.14	0.61	4.87	7.38E-05	0.0015	UPK1A	uroplakin 1A
16764894	6.12	3.84	0.6	0.89	4.86	2.99E-06	0.0001	KRT6B	keratin 6B, type II
16966621	7.04	4.76	0.3	0.81	4.86	1.62E-07	1.66E-05	CWH43	cell wall biogenesis 43 C-terminal homolog
16819257	3.82	1.54	1.63	0.95	4.85	0.0009	0.0105	MT1H	metallothionein 1H
17102566	6.4	4.13	0.85	1.13	4.84	0.0003	0.0039	SYTL5	synaptotagmin-like 5
16968428	3.56	1.29	1.23	0.69	4.84	0.0003	0.0048		
16762337	7.39	5.12	0.79	0.39	4.83	6.12E-07	4.10E-05	BCAT1	branched chain amino-acid transaminase 1, cytosolic
16874740	9.21	6.95	0.44	0.53	4.8	3.66E-09	1.30E-06	KLK11	kallikrein related peptidase 11
17028938	4.02	1.77	1.39	0.61	4.77	0.0002	0.0028		
17076285	6.46	4.22	0.82	0.26	4.75	2.79E-07	2.44E-05	RAB11FIP1	RAB11 family interacting protein 1 (class I)
16687602	6.31	4.07	0.52	0.29	4.72	1.44E-08	3.33E-06	TTC22	tetratricopeptide repeat domain 22
16965606	5.75	3.53	1.03	0.63	4.66	2.09E-05	0.0006	SLC34A2	solute carrier family 34 (type II sodium/phosphate cotransporter), member 2
16693393	5.29	3.07	0.78	1.08	4.66	7.86E-05	0.0016	PGLYRP4	peptidoglycan recognition protein 4
16975777	4.83	2.61	0.64	0.61	4.64	1.52E-06	7.95E-05	TEC	tec protein tyrosine kinase
16839177	5.59	3.38	0.37	0.7	4.63	9.98E-08	1.19E-05	METRNL	meteorin, glial cell differentiation regulator-like
16693409	7.21	5.01	0.77	0.7	4.61	3.71E-06	0.0002	S100A12	S100 calcium binding protein A12
17093920	8.56	6.36	0.52	0.49	4.59	2.23E-08	4.36E-06	GNE	glucosamine (UDP-N-acetyl)-2-epimerase/N-acetylmannosamine kinase
16671104	10.88	8.69	0.23	1.18	4.56	6.31E-06	0.0002	SPRR1A	small proline-rich protein 1A
16906175	4.04	1.85	0.93	0.24	4.55	5.50E-06	0.0002	FRZB	frizzled-related protein
16949264	6.21	4.03	0.94	0.46	4.54	0.0001	0.0025	LOC344887	NmrA-like family domain containing 1 pseudogene
16929368	3.59	1.42	0.52	0.24	4.52	3.35E-09	1.27E-06	SLC5A1	solute carrier family 5 (sodium/glucose cotransporter), member 1
17073213	5.65	3.48	0.84	1.05	4.51	9.60E-05	0.0018	PSCA	prostate stem cell antigen
17008092	8.16	5.98	0.33	0.38	4.51	4.69E-10	3.90E-07	PIM1	Pim-1 proto-oncogene, serine/threonine kinase
16914925	6.23	4.06	1.14	0.39	4.49	7.05E-05	0.0014	TSHZ2	teashirt zinc finger homeobox 2
17081957	4.21	2.04	0.74	0.46	4.49	4.04E-07	3.13E-05	LYNX1	Ly6/neurotoxin 1
17060287	6.92	4.76	0.6	1.01	4.47	2.55E-06	0.0001	CYP3A5	cytochrome P450, family 3, subfamily A, polypeptide 5
16837915	4.14	1.98	0.57	0.35	4.47	5.70E-08	8.31E-06	SMIM5	small integral membrane protein 5

16980026	4.28	2.12	1.09	0.74	4.45	0.0001	0.002	LOC101927490	uncharacterized LOC101927490
16874765	3.93	1.78	0.98	0.17	4.44	2.44E-06	0.0001	KLK13	kallikrein related peptidase 13
16717005	5.56	3.41	0.56	0.47	4.43	4.21E-09	1.43E-06	PIK3AP1	phosphoinositide-3-kinase adaptor protein 1
16874702	9.52	7.38	0.54	0.5	4.43	4.97E-08	7.64E-06	KLK7	kallikrein related peptidase 7
16703642	5.05	2.91	0.66	0.43	4.41	1.29E-07	1.43E-05	MAP3K8	mitogen-activated protein kinase kinase kinase 8
17025046	5.95	3.82	0.58	0.45	4.39	1.73E-07	1.73E-05	CNKSR3	CNKSR family member 3
17060049	4.55	2.42	0.49	0.52	4.37	8.62E-08	1.07E-05	DLX5	distal-less homeobox 5
16818820	3.78	1.66	0.4	0.63	4.35	3.34E-08	5.73E-06	NOD2	nucleotide-binding oligomerization domain containing 2
16874693	7.64	5.52	0.91	0.69	4.35	1.37E-05	0.0004	KLK6	kallikrein related peptidase 6
17013657	6.03	3.91	0.45	0.75	4.34	2.58E-07	2.30E-05	ULBP1	UL16 binding protein 1
16828471	4.77	2.65	1.22	0.9	4.34	0.0004	0.0056	CHST6	carbohydrate (N-acetylglucosamine 6-O) sulfotransferase 6
16825647	7.55	5.44	0.45	0.73	4.33	9.38E-07	5.62E-05	GDPD3	glycerophosphodiester phosphodiesterase domain containing 3
16785943	6.98	4.87	0.73	0.74	4.31	1.55E-05	0.0005	LINC01269	long intergenic non-protein coding RNA 1269
16698766	6.05	3.95	0.46	0.44	4.28	2.14E-08	4.30E-06	YOD1	YOD1 deubiquitinase
16790143	4.18	2.09	0.62	0.14	4.27	1.06E-08	2.77E-06	NDRG2	NDRG family member 2
17017363	5.75	3.65	1.06	0.54	4.27	2.17E-05	0.0006	LY6G6C	lymphocyte antigen 6 complex, locus G6C
17029219	5.75	3.65	1.06	0.54	4.27	2.17E-05	0.0006		
17032004	5.75	3.65	1.06	0.54	4.27	2.17E-05	0.0006		
17034474	5.75	3.65	1.06	0.54	4.27	2.17E-05	0.0006		
17036722	5.75	3.65	1.06	0.54	4.27	2.17E-05	0.0006		
17042000	5.75	3.65	1.06	0.54	4.27	2.17E-05	0.0006		
17092901	7.08	4.99	0.39	0.64	4.26	5.28E-08	7.88E-06	CDKN2B	cyclin-dependent kinase inhibitor 2B (p15, inhibits CDK4)
16670681	4.54	2.45	1.44	0.48	4.25	0.0011	0.0119	ANXA9	annexin A9
17078943	9.56	7.47	0.93	1.01	4.24	0.0001	0.0022	CALB1	calbindin 1
16945682	6.54	4.46	0.73	0.9	4.23	5.07E-05	0.0011	ACPP	acid phosphatase, prostate
16873528	3.25	1.17	1.89	0.39	4.22	0.0007	0.0084	LOC645553	uncharacterized LOC645553
16695535	3.96	1.89	0.65	0.64	4.22	7.34E-07	4.72E-05	ARHGAP30	Rho GTPase activating protein 30
16666799	9.86	7.79	1.07	1.19	4.2	0.0006	0.0082	CLCA4	chloride channel accessory 4
16980727	7.06	4.99	0.81	0.36	4.19	1.20E-06	6.70E-05		
17039517	5.19	3.12	1.2	0.44	4.18	4.20E-05	0.001		
17123804	3.28	1.22	1.11	0.18	4.17	0.0001	0.0021		
16880707	3.14	1.08	1.22	0.95	4.17	0.0009	0.0106		
16864427	5.19	3.14	0.58	0.67	4.15	1.13E-06	6.40E-05	MYH14	myosin, heavy chain 14, non-muscle
16990718	10.25	8.2	0.42	1.08	4.15	1.26E-05	0.0004	SPINK5	serine peptidase inhibitor, Kazal type 5
16670479	5.62	3.57	0.47	0.61	4.15	1.37E-07	1.49E-05	CIART	circadian associated repressor of transcription
16699545	4.91	2.86	0.73	0.48	4.14	2.93E-07	2.51E-05	LOC101929771	uncharacterized LOC101929771
16962316	7.21	5.16	0.54	0.56	4.14	4.26E-07	3.24E-05	LIPH	lipase, member H
17000168	8.85	6.8	1.05	1.34	4.14	0.0038	0.0329	CXCL14	chemokine (C-X-C motif) ligand 14
16695558	7.85	5.81	0.42	0.7	4.13	2.68E-07	2.36E-05	PVRL4	poliovirus receptor-related 4
16670710	6.53	4.49	0.52	0.68	4.13	5.31E-07	3.72E-05	BNIPL	BCL2/adenovirus E1B 19kD interacting protein like
16715212	3.73	1.69	0.88	0.19	4.11	5.72E-06	0.0002	UNC5B-AS1	UNC5B antisense RNA 1
16955197	5.18	3.14	0.47	0.32	4.09	3.52E-09	1.29E-06	WNT5A	wingless-type MMTV integration site family, member 5A
16728617	5	2.98	0.62	0.33	4.05	2.09E-07	1.97E-05	FOLR3	folate receptor 3 (gamma)
16844294	7.77	5.76	0.7	0.88	4.02	7.41E-05	0.0015	NR1D1	nuclear receptor subfamily 1, group D, member 1
17002846	9.71	7.7	0.42	0.4	4.01	5.01E-09	1.63E-06	DUSP1	dual specificity phosphatase 1
16741142	7.9	5.91	0.75	1.04	3.97	7.30E-05	0.0015	ALDH3B2	aldehyde dehydrogenase 3 family, member B2
16697095	8.22	6.24	0.56	0.47	3.94	8.63E-08	1.07E-05	NCF2	neutrophil cytosolic factor 2
16799662	3.39	1.41	0.8	0.15	3.93	4.93E-07	3.56E-05	C15orf62	chromosome 15 open reading frame 62
16919531	7.03	5.06	1.13	0.91	3.93	0.0006	0.0082	WFDC5	WAP four-disulfide core domain 5
16872803	7.03	5.05	0.54	0.4	3.92	7.93E-08	1.01E-05	CEACAM1	carcinoembryonic antigen-related cell adhesion molecule 1 (biliary glycoprotein)
16666055	6.23	4.26	0.38	0.56	3.91	1.84E-08	3.83E-06	CTH	cystathionine gamma-lyase
16750974	6.74	4.77	1.32	1.22	3.91	0.002	0.0197	AQP5	aquaporin 5
16971737	6.83	4.87	0.8	0.76	3.9	4.86E-05	0.0011	GUCY1B3	guanylate cyclase 1, soluble, beta 3
16840817	5.91	3.95	0.42	0.5	3.89	1.43E-08	3.32E-06	ALOXE3	arachidonate lipoxygenase 3
16836528	4.81	2.86	0.71	0.74	3.88	4.59E-06	0.0002	YPEL2	yippee like 2
16713779	6.66	4.71	0.53	0.48	3.87	3.11E-07	2.60E-05	FAM25C; FAM25G	family with sequence similarity 25, member C; family with sequence similarity 25, mer
16686201	7.08	5.13	0.3	0.67	3.86	1.22E-07	1.38E-05	SLC6A9	solute carrier family 6 (neurotransmitter transporter, glycine), member 9
17123272	3.87	1.93	1.37	0.32	3.84	0.0004	0.006		
17125182	3.16	1.22	0.53	0.56	3.82	4.51E-07	3.36E-05		
16997143	2.87	0.94	1.06	0.77	3.81	0.0008	0.0091	LOC105379031	uncharacterized LOC105379031
17100671	4.91	2.98	1.03	0.46	3.81	0.0001	0.0022		
16693308	7.29	5.37	0.7	0.95	3.79	3.93E-05	0.0009	FLG	filaggrin
16785938	8.65	6.72	0.37	0.31	3.79	8.55E-10	5.89E-07	TTC9	tetratricopeptide repeat domain 9
16704607	6.65	4.73	0.53	0.46	3.79	3.43E-07	2.80E-05	FAM25C; FAM25G	family with sequence similarity 25, member C; family with sequence similarity 25, mer
16910284	2.95	1.03	1.08	0.32	3.77	0.0001	0.002	C2orf54	chromosome 2 open reading frame 54
16670185	7.33	5.42	0.63	1.31	3.77	2.48E-05	0.0006	LOC284561	uncharacterized LOC284561
16698185	5.11	3.2	0.74	0.79	3.76	0.0001	0.002	CHI3L1	chitinase 3-like 1 (cartilage glycoprotein-39)
16693406	7.44	5.53	0.33	0.51	3.76	3.23E-08	5.60E-06		
16919538	2.9	0.99	0.66	0.34	3.75	3.13E-07	2.62E-05	WFDC12	WAP four-disulfide core domain 12
16975737	4.83	2.94	0.61	0.74	3.71	1.14E-05	0.0004	CNGA1	cyclic nucleotide gated channel alpha 1
16900875	3.41	1.52	0.63	0.31	3.71	5.06E-07	3.60E-05	MGAT4A	mannosyl (alpha-1,3-)-glycoprotein beta-1,4-N-acetylglucosaminyltransferase, isozyme
16866290	6.67	4.78	0.56	0.6	3.69	1.03E-06	6.03E-05	RNF225	ring finger protein 225
16667778	3.06	1.19	0.82	0.45	3.66	4.00E-05	0.0009	LOC105378872	uncharacterized LOC105378872
16785709	4.2	2.34	1.03	0.96	3.65	1.55E-05	0.0005	RDH12	retinol dehydrogenase 12 (all-trans/9-cis/11-cis)
16874731	8.64	6.77	0.47	0.39	3.64	3.34E-08	5.73E-06	KLK10	kallikrein related peptidase 10
16977760	7.04	5.18	0.31	0.65	3.61	3.57E-07	2.87E-05	SLC10A6	solute carrier family 10 (sodium/bile acid cotransporter), member 6
16671632	7.27	5.42	0.3	0.65	3.61	5.54E-08	8.18E-06	EFNA3	ephrin-A3
16683541	6.26	4.41	0.64	0.34	3.61	6.21E-07	4.14E-05	IL22RA1	interleukin 22 receptor, alpha 1
17119822	5.21	3.36	0.4	1.1	3.6	7.55E-05	0.0015		
16763650	3.5	1.65	1.37	0.39	3.59	0.0013	0.0144	ENDOU	endonuclease, polyU-specific

17124140	6.71	4.87	0.86	1.04	3.59	0.0007	0.0088		
16801604	4.63	2.79	0.8	0.29	3.58	2.41E-06	0.0001	GCNT3	glucosaminyl (N-acetyl) transferase 3, mucin type
16948572	5.57	3.73	0.34	0.21	3.57	4.10E-11	1.39E-07	KLHL24	kelch-like family member 24
16777190	8.5	6.67	0.68	0.89	3.55	5.00E-05	0.0011	GJB6	gap junction protein beta 6
16744991	8.69	6.86	0.27	0.33	3.55	1.18E-09	7.24E-07	MPZL3	myelin protein zero-like 3
16845937	7.05	5.24	0.69	0.54	3.52	5.23E-06	0.0002	PLEKHM1	pleckstrin homology domain containing, family M (with RUN domain) member 1
17019877	2.85	1.04	0.92	0.2	3.51	1.76E-05	0.0005	CRISP3	cysteine-rich secretory protein 3
16983765	6.39	4.58	0.67	0.97	3.51	6.46E-05	0.0013	NPR3	natriuretic peptide receptor 3
16676764	8.79	6.99	0.56	0.33	3.5	7.50E-08	9.95E-06	CD55	CD55 molecule, decay accelerating factor for complement (Cromer blood group)
16840976	3.86	2.05	0.73	0.34	3.5	1.08E-06	6.21E-05	RNF222	ring finger protein 222
16923145	6.1	4.29	0.31	0.44	3.5	1.60E-08	3.58E-06	ABCG1	ATP binding cassette subfamily G member 1
17058152	7.18	5.38	0.51	0.38	3.49	6.19E-08	8.80E-06	ERV3-1; ZNF117	endogenous retrovirus group 3, member 1; zinc finger protein 117
16850344	9.79	7.99	0.37	0.25	3.48	1.04E-09	6.79E-07	MIR4315-1; MIR4:	microRNA 4315-1; microRNA 4315-2; pleckstrin homology domain containing, family M
16671100	4.22	2.42	0.87	0.39	3.47	2.70E-05	0.0007	SPRR4	small proline-rich protein 4
17058142	5.63	3.84	0.7	0.7	3.46	3.72E-05	0.0009	ZNF117	zinc finger protein 117
16802497	6.22	4.43	0.85	1.15	3.46	0.0062	0.048	PAQR5	progestin and adipoQ receptor family member V
17047795	3.42	1.63	0.93	0.49	3.46	6.32E-05	0.0013	CD36	CD36 molecule (thrombospondin receptor)
16693331	2.48	0.69	1.45	0.28	3.45	0.0007	0.0086	LCE3E	late cornified envelope 3E
17039213	2.78	1	1.27	0.35	3.45	6.11E-05	0.0013		
16938271	7.18	5.39	0.49	0.28	3.44	4.77E-08	7.49E-06	KAT2B	K(lysine) acetyltransferase 2B
16929562	4.35	2.57	0.89	0.34	3.44	1.69E-05	0.0005	HMOX1	heme oxygenase 1
16667783	2.4	0.62	1.06	0.21	3.43	2.37E-05	0.0006	DNAJA1P5	DnaJ (Hsp40) homolog, subfamily A, member 1 pseudogene 5
16874005	7.53	5.76	0.37	0.91	3.42	2.53E-05	0.0006	DBP	D site of albumin promoter (albumin D-box) binding protein
16833060	4.39	2.62	0.61	0.36	3.41	9.26E-07	5.59E-05	LRRC37B	leucine rich repeat containing 37B
16751552	3.48	1.72	0.72	0.31	3.39	1.51E-06	7.87E-05		
16884607	3.86	2.1	0.8	0.42	3.39	5.57E-06	0.0002	IL36RN	interleukin 36 receptor antagonist
16875559	4.79	3.03	0.75	0.33	3.39	7.76E-06	0.0003	RDH13	retinol dehydrogenase 13 (all-trans/9-cis)
16915712	3.47	1.71	0.44	0.41	3.38	2.97E-07	2.54E-05	SLCO4A1	solute carrier organic anion transporter family, member 4A1
16729323	3.35	1.61	0.86	0.34	3.36	3.48E-06	0.0001	B3GNT6	UDP-GlcNAc:betaGal beta-1,3-N-acetylglucosaminyltransferase 6
16713955	7.64	5.89	0.56	0.47	3.35	1.46E-06	7.70E-05	FAM25C; FAM25G	family with sequence similarity 25, member C; family with sequence similarity 25, mem
17013520	7.07	5.33	0.57	0.59	3.35	1.32E-05	0.0004	SASH1	SAM and SH3 domain containing 1
16925328	3.42	1.67	0.91	0.59	3.35	0.0005	0.007	KCNE1	potassium channel, voltage gated subfamily E regulatory beta subunit 1
16828108	8.36	6.63	0.98	0.56	3.33	0.0002	0.0032	SNORD71	small nucleolar RNA, C/D box 71
16867572	6.18	4.45	0.64	0.6	3.33	4.18E-06	0.0002	FUT3	fucosyltransferase 3 (galactoside 3(4)-L-fucosyltransferase, Lewis blood group)
16709610	9.15	7.42	0.5	0.85	3.32	2.32E-05	0.0006	PNLIPRP3	pancreatic lipase-related protein 3
16996387	8.01	6.28	0.69	0.18	3.31	4.86E-06	0.0002	MIER3	mesoderm induction early response 1, family member 3
16664608	3.72	2	0.51	0.27	3.31	1.22E-07	1.38E-05		
17009426	3.17	1.44	0.65	0.4	3.3	1.21E-06	6.74E-05	ADGRF2; ADGRF4	adhesion G protein-coupled receptor F2; adhesion G protein-coupled receptor F4
17099466	4.89	3.17	0.74	0.73	3.29	2.43E-05	0.0006	ABO	ABO blood group (transferase A, alpha 1-3-N-acetylgalactosaminyltransferase; transfe
17005223	4.49	2.78	0.69	0.71	3.28	0.0003	0.0048	ID4	inhibitor of DNA binding 4, dominant negative helix-loop-helix protein
16923239	4.62	2.91	0.68	0.31	3.27	3.08E-06	0.0001	PDE9A	phosphodiesterase 9A
16881138	9.2	7.5	0.34	0.31	3.26	3.68E-09	1.30E-06	MXD1	MAX dimerization protein 1
16671139	10.19	8.49	0.39	0.51	3.26	2.90E-07	2.50E-05	S100A9	S100 calcium binding protein A9
16705220	2.76	1.06	0.79	0.17	3.25	1.24E-06	6.91E-05	ZNF365	zinc finger protein 365
16738803	7.68	5.98	0.38	1.61	3.24	0.0006	0.0073	TCN1	transcobalamin I (vitamin B12 binding protein, R binder family)
16976599	5.01	3.31	0.85	0.71	3.24	0.0001	0.0023	SULT1B1	sulfotransferase family 1B member 1
16748015	9.82	8.13	0.28	0.71	3.23	1.46E-06	7.69E-05	A2ML1	alpha-2-macroglobulin-like 1
16862563	10.46	8.78	0.48	0.53	3.21	4.07E-06	0.0002	CEACAM6	carcinoembryonic antigen-related cell adhesion molecule 6 (non-specific cross reacting
16767020	3.18	1.5	0.85	0.49	3.21	6.03E-05	0.0013	C12orf56	chromosome 12 open reading frame 56
16961056	6.77	5.1	0.85	0.93	3.2	0.0016	0.0165	SPTSSB	serine palmitoyltransferase, small subunit B
16729485	4.67	3	0.71	0.22	3.19	2.55E-06	0.0001	USP35	ubiquitin specific peptidase 35
16792601	4.76	3.09	0.48	0.31	3.18	3.73E-08	6.29E-06	LOC105378179	uncharacterized LOC105378179
17124354	5.77	4.1	0.84	1.17	3.17	0.0004	0.0054		
16720307	6.3	4.64	0.59	0.29	3.17	2.67E-06	0.0001	TMEM80	transmembrane protein 80
16746546	5.75	4.09	0.33	0.62	3.15	4.94E-07	3.56E-05	B4GALNT3	beta-1,4-N-acetyl-galactosaminyl transferase 3
16809913	4.72	3.07	0.47	0.74	3.14	1.70E-05	0.0005		
17001005	6.63	4.99	0.38	0.32	3.13	2.94E-08	5.24E-06	PCDH1	protocadherin 1
17061545	4.56	2.92	0.52	0.48	3.12	1.61E-06	8.27E-05	NAMPT	nicotinamide phosphoribosyltransferase
16855244	2.37	0.73	0.7	0.28	3.11	7.34E-06	0.0003	MIR4320	microRNA 4320
16861997	8.69	7.05	0.42	0.4	3.11	5.60E-07	3.84E-05	ZFP36	ZFP36 ring finger protein
16997275	6.57	4.94	0.4	0.3	3.09	1.58E-08	3.57E-06	GCNT4	glucosaminyl (N-acetyl) transferase 4, core 2
16732397	4.48	2.85	0.61	0.55	3.09	9.33E-06	0.0003	POU2F3	POU class 2 homeobox 3
17123814	2.53	0.91	1.06	0.15	3.07	0.0002	0.0031		
16698590	4.28	2.67	0.73	0.5	3.06	2.79E-05	0.0007	SLC26A9	solute carrier family 26 (anion exchanger), member 9
16682144	6.27	4.66	0.61	0.47	3.05	1.39E-05	0.0004	ANO7P1	anoctamin 7 pseudogene 1
17054439	5.86	4.25	0.4	0.49	3.04	1.59E-07	1.63E-05	TMEM184A	transmembrane protein 184A
16874722	4.05	2.45	0.45	0.59	3.04	8.33E-06	0.0003	KLK9; KLK8	kallikrein related peptidase 9; kallikrein related peptidase 8
16992557	6.56	4.96	0.27	0.28	3.03	7.17E-10	5.32E-07	CPEB4	cytoplasmic polyadenylation element binding protein 4
16876978	8.54	6.94	0.42	0.62	3.03	6.34E-07	4.22E-05	GRHL1	grainyhead-like transcription factor 1
16974493	3	1.41	0.52	0.37	3.02	4.03E-07	3.12E-05	LOC105374500	uncharacterized LOC105374500
16977309	3.55	1.95	0.54	0.24	3.02	3.32E-07	2.73E-05	RASGEF1B	RasGEF domain family member 1B
16947613	3.12	1.53	0.3	0.47	3.02	7.02E-08	9.52E-06	PPM1L	protein phosphatase, Mg2+/Mn2+ dependent, 1L
17100201	6.75	5.16	0.45	0.58	3.02	1.72E-06	8.67E-05	CLIC3	chloride intracellular channel 3
16910609	5.99	4.4	0.51	0.67	3.02	3.18E-05	0.0008	TRIB3	tribbles pseudokinase 3
16789149	3.83	2.24	0.45	0.45	3.01	8.42E-06	0.0003	TNFAIP2	tumor necrosis factor, alpha-induced protein 2
16992467	4.54	2.95	0.43	0.15	3	6.78E-09	2.07E-06	CREBRF	CREB3 regulatory factor
16975971	4.56	2.98	0.78	0.51	3	6.26E-05	0.0013	LNX1	ligand of numb-protein X 1, E3 ubiquitin protein ligase
16709136	2.97	1.4	0.74	0.26	2.99	1.15E-05	0.0004		
17061125	5.12	3.54	1.02	1.09	2.98	0.0028	0.0259	RASA4B; RASA4	RAS p21 protein activator 4B; RAS p21 protein activator 4
16825638	6.06	4.49	0.33	0.23	2.98	1.95E-09	9.20E-07	YPEL3	yippee like 3

16919547	11.47	9.9	0.22	0.58	2.97	1.47E-07	1.56E-05	SLPI	secretory leukocyte peptidase inhibitor
16742647	2.45	0.88	0.28	0.22	2.96	2.58E-10	2.97E-07	GAB2	GRB2-associated binding protein 2
17121344	4.43	2.87	0.68	0.31	2.95	6.53E-05	0.0013		
16761631	6.4	4.84	0.61	0.46	2.95	3.43E-06	0.0001	DUSP16	dual specificity phosphatase 16
16687479	5.52	3.97	0.89	0.7	2.94	0.0002	0.0038	LDLRAD1	low density lipoprotein receptor class A domain containing 1
17101341	4.17	2.62	0.3	0.44	2.93	7.72E-07	4.90E-05	TBL1X	transducin (beta)-like 1X-linked
17098698	7.75	6.2	0.19	0.56	2.93	3.98E-08	6.57E-06	FAM102A	family with sequence similarity 102, member A
16874713	6.77	5.22	0.56	0.73	2.93	6.47E-06	0.0002	KLK8	kallikrein related peptidase 8
16794318	3.51	1.96	0.34	0.39	2.93	7.54E-08	9.95E-06	ADAM20P1	ADAM metallopeptidase domain 20 pseudogene 1
16953321	2.29	0.75	1.01	0.16	2.92	0.0002	0.0037	SPINK8	serine peptidase inhibitor, Kazal type 8 (putative)
16976430	3.46	1.92	1.07	0.36	2.91	0.0008	0.0095	GNRHR	gonadotropin releasing hormone receptor
16875034	6.72	5.19	0.47	0.38	2.9	1.34E-06	7.25E-05	ZNF836	zinc finger protein 836
16976468	7.06	5.52	0.6	0.67	2.9	8.88E-05	0.0017	TMPRSS11F	transmembrane protease, serine 11F
16891555	7.13	5.59	0.64	0.69	2.9	9.26E-05	0.0018	SGPP2	sphingosine-1-phosphate phosphatase 2
16733288	3.67	2.13	0.76	0.3	2.89	0.0002	0.0031	ST3GAL4	ST3 beta-galactoside alpha-2,3-sialyltransferase 4
17021596	4.86	3.34	0.27	0.7	2.87	5.95E-07	4.01E-05	RRAGD	Ras-related GTP binding D
16990862	7.22	5.7	0.65	1.27	2.87	0.003	0.0275	ABLIM3	actin binding LIM protein family, member 3
16929529	5.93	4.41	0.37	0.24	2.87	1.43E-08	3.32E-06	TOM1	target of myb1 membrane trafficking protein
17118792	6.14	4.62	0.29	0.44	2.86	2.60E-08	4.75E-06		
16687875	7.46	5.94	0.56	0.4	2.86	2.24E-06	0.0001	JUN	jun proto-oncogene
16844026	3.27	1.76	0.66	0.37	2.86	0.0001	0.0022	C17orf98	chromosome 17 open reading frame 98
16898578	9.58	8.07	0.29	0.18	2.85	2.16E-09	1.00E-06	GFPT1	glutamine--fructose-6-phosphate transaminase 1
16947111	6.87	5.37	0.65	0.53	2.84	1.73E-05	0.0005	P2RY1	purinergic receptor P2Y, G-protein coupled, 1
16965162	7.82	6.31	0.32	0.36	2.84	2.27E-08	4.36E-06	CPEB2	cytoplasmic polyadenylation element binding protein 2
16927082	3.5	1.99	0.45	0.45	2.84	3.27E-05	0.0008	GP1BB; SEPT5	glycoprotein Ib (platelet), beta polypeptide; septin 5
17124276	2.05	0.55	0.79	0.3	2.83	4.55E-05	0.001		
17122830	3.24	1.74	0.65	0.31	2.83	3.00E-05	0.0007		
16836511	5.28	3.78	0.39	0.44	2.82	2.09E-07	1.97E-05	GDPD1	glycerophosphodiester phosphodiesterase domain containing 1
17062457	4.05	2.55	0.4	0.44	2.82	5.70E-07	3.87E-05		
16934671	8.03	6.54	0.46	0.29	2.82	1.33E-06	7.23E-05	TST	thiosulfate sulfurtransferase (rhodanese)
16698764	4.52	3.03	0.87	0.81	2.82	0.002	0.0199		
16863287	3.52	2.04	0.5	0.41	2.81	8.26E-07	5.18E-05	FOSB	FBJ murine osteosarcoma viral oncogene homolog B
17013694	7.14	5.65	0.37	0.77	2.8	6.74E-06	0.0002	PLEKHG1	pleckstrin homology domain containing, family G (with RhoGef domain) member 1
17123246	3.43	1.94	0.36	0.39	2.8	4.31E-07	3.26E-05		
16840417	4.29	2.8	0.98	0.53	2.8	0.0007	0.0086	SLC13A5	solute carrier family 13 (sodium-dependent citrate transporter), member 5
16715529	6.97	5.49	0.48	0.36	2.79	5.11E-07	3.62E-05	USP54	ubiquitin specific peptidase 54
17124376	2.77	1.29	0.93	0.26	2.79	0.0001	0.0024		
16709108	4.74	3.26	0.37	0.28	2.78	5.04E-08	7.65E-06	MXI1	MAX interactor 1, dimerization protein
16922047	2.75	1.28	0.87	0.28	2.77	0.0009	0.0109		
17025005	5.62	4.15	0.58	0.34	2.77	3.24E-06	0.0001	RGS17	regulator of G-protein signaling 17
16749939	2.99	1.52	0.73	0.47	2.77	0.0003	0.0049	LRRK2	leucine-rich repeat kinase 2
16981606	5.2	3.73	0.67	0.7	2.77	4.61E-05	0.001	HPGD	hydroxyprostaglandin dehydrogenase 15-(NAD)
16939590	2.83	1.36	0.68	0.37	2.76	3.15E-05	0.0008		
16911261	5.65	4.19	0.43	0.6	2.76	1.46E-05	0.0004	BMP2	bone morphogenetic protein 2
16671108	7.99	6.53	0.77	1.06	2.76	0.0001	0.0019	SPRR3	small proline-rich protein 3
16775546	10.31	8.84	0.3	0.24	2.76	5.08E-09	1.64E-06	SCEL	sciellin
16824706	6.35	4.88	0.32	0.23	2.76	2.97E-09	1.18E-06	DCUN1D3; ERI2	DCN1, defective in cullin neddylation 1, domain containing 3; ERI1 exoribonuclease far
16993235	2.43	0.97	0.8	0.4	2.75	0.0003	0.0041	N4BP3	NEDD4 binding protein 3
16863877	6.49	5.03	0.41	0.28	2.75	7.05E-08	9.52E-06	PPP1R15A	protein phosphatase 1, regulatory subunit 15A
17023799	4.22	2.77	0.62	0.51	2.74	2.09E-05	0.0006	SLC2A12	solute carrier family 2 (facilitated glucose transporter), member 12
17119076	4.78	3.33	0.49	0.87	2.73	0.0003	0.0049		
16709128	5.53	4.09	0.38	0.69	2.73	1.70E-05	0.0005	DUSP5	dual specificity phosphatase 5
16840915	2.7	1.25	0.61	0.29	2.73	2.73E-06	0.0001	LINC00324	long intergenic non-protein coding RNA 324
16823215	6.56	5.11	0.66	0.57	2.72	0.0001	0.0019	PRSS22	protease, serine, 22
16996564	4.83	3.39	0.54	0.29	2.72	5.30E-06	0.0002	ELOVL7	ELOVL fatty acid elongase 7
16964735	2.9	1.46	0.65	0.35	2.72	3.44E-05	0.0008	S100P	S100 calcium binding protein P
16979917	10.13	8.69	0.51	0.6	2.72	3.63E-05	0.0009	SLC7A11	solute carrier family 7 (anionic amino acid transporter light chain, xc- system), membe
16975751	2.34	0.9	0.71	0.19	2.72	0.0007	0.0084	TXK	TXK tyrosine kinase
16998551	2.86	1.41	1.03	0.38	2.72	0.0017	0.0175	SLCO4C1	solute carrier organic anion transporter family, member 4C1
16693357	2.2	0.77	1.1	0.32	2.71	0.0011	0.012	SPRR2A	small proline-rich protein 2A
16863864	6.31	4.87	0.62	0.83	2.71	0.0007	0.0086	FUT2	fucosyltransferase 2 (secretor status included)
16902002	2.06	0.63	1.23	0.13	2.7	0.0006	0.0076	IL36B	interleukin 36, beta
16791219	10.16	8.72	0.65	0.76	2.7	0.0013	0.014	TGM1	transglutaminase 1
16762759	4.51	3.09	0.64	0.37	2.69	0.0001	0.0021	TMTC1	transmembrane and tetratricopeptide repeat containing 1
16867915	4.48	3.05	0.73	0.36	2.69	1.82E-05	0.0005	INSR	insulin receptor
16922078	2.91	1.49	0.62	0.83	2.69	0.0011	0.0123	KRTAP20-1	keratin associated protein 20-1
16986583	6.78	5.35	0.31	0.32	2.69	8.26E-09	2.33E-06	JMY	junction mediating and regulatory protein, p53 cofactor
16948967	4.16	2.74	0.64	0.7	2.68	0.0002	0.0038	SNORD66	small nucleolar RNA, C/D box 66
16926111	3.52	2.1	0.48	0.2	2.68	9.70E-08	1.17E-05	CBS; CBSL	cystathionine-beta-synthase; cystathionine-beta-synthase like
17064325	2.31	0.89	0.57	0.32	2.67	4.99E-06	0.0002	ATG9B	autophagy related 9B
16875952	3.35	1.93	0.74	0.33	2.67	3.78E-05	0.0009	VN1R1	vomeronasal 1 receptor 1
16855111	3.8	2.38	0.44	0.55	2.66	4.16E-06	0.0002	ZBTB7C	zinc finger and BTB domain containing 7C
17083197	8.56	7.15	0.16	0.3	2.66	1.38E-09	8.14E-07	VLDLR	very low density lipoprotein receptor
17117424	5.37	3.97	0.59	0.44	2.65	1.52E-05	0.0004	LOC100287497; L	uncharacterized LOC100287497; uncharacterized LOC101926980
16949792	3.46	2.06	0.54	0.16	2.65	6.66E-07	4.39E-05	FAM43A	family with sequence similarity 43, member A
17120106	7.25	5.85	0.57	0.35	2.65	5.06E-06	0.0002		
16947107	8.12	6.72	0.53	0.66	2.64	4.35E-05	0.001	P2RY1	purinergic receptor P2Y, G-protein coupled, 1
16845537	4.63	3.24	0.49	0.31	2.63	6.93E-07	4.52E-05	HDAC5	histone deacetylase 5
16857021	3.67	2.28	0.74	0.63	2.63	0.0002	0.003	TJP3	tight junction protein 3
16850294	8.89	7.5	0.35	1.09	2.62	0.0002	0.0026	ZNF750	zinc finger protein 750

16801400	3.11	1.72	0.76	0.43	2.62	1.64E-05	0.0005	CGNL1	cingulin-like 1
16658536	7.24	5.85	0.39	0.37	2.62	1.07E-06	6.15E-05	PER3	period circadian clock 3
17061110	4.55	3.16	0.7	0.83	2.62	0.0008	0.0094	RASA4B; RASA4	RAS p21 protein activator 4B; RAS p21 protein activator 4
16785696	7.11	5.72	0.45	0.57	2.61	1.65E-05	0.0005	ARG2	arginase 2
17118269	4.88	3.49	0.87	0.59	2.61	0.0019	0.0192		
17072601	6.67	5.29	0.14	0.33	2.61	3.11E-09	1.21E-06	TRIB1	tribbles pseudokinase 1
16857676	4.93	3.55	0.4	0.17	2.6	3.19E-08	5.55E-06	CAMSAP3	calmodulin regulated spectrin-associated protein family, member 3
16880291	4.83	3.45	0.84	0.78	2.6	0.0002	0.0029		
16800630	2.56	1.18	0.97	0.3	2.6	0.0003	0.0048	C15orf48; MIR147	chromosome 15 open reading frame 48; microRNA 147b
17124314	2.12	0.75	1	0.23	2.59	0.0008	0.0098		
16938378	7.18	5.8	0.25	0.48	2.59	5.03E-07	3.59E-05	NR1D2	nuclear receptor subfamily 1, group D, member 2
16781648	3.44	2.06	0.58	0.47	2.59	4.36E-05	0.001	SLC39A2	solute carrier family 39 (zinc transporter), member 2
16775498	2.17	0.8	0.65	0.66	2.58	0.0015	0.0157	LMO7DN-IT1	LMO7DN intronic transcript 1
16847343	2.43	1.07	0.74	0.23	2.58	2.35E-05	0.0006	WFDC21P	WAP four-disulfide core domain 21, pseudogene
16819909	4.31	2.95	0.51	0.72	2.57	0.0001	0.0023	CES3	carboxylesterase 3
17079317	7.31	5.96	0.22	0.39	2.56	2.14E-08	4.30E-06	TP53INP1	tumor protein p53 inducible nuclear protein 1
16700515	5.18	3.83	0.38	0.7	2.56	4.37E-05	0.001	SIPA1L2	signal-induced proliferation-associated 1 like 2
16752397	6.01	4.66	0.54	0.32	2.56	2.52E-06	0.0001	ERBB3	erb-b2 receptor tyrosine kinase 3
16704865	2.79	1.43	0.99	0.39	2.56	0.0012	0.0128	MSMB	microseminoprotein, beta-
17100651	9.73	8.38	0.79	0.49	2.56	0.0007	0.009		
16876842	4.04	2.69	0.72	0.7	2.56	0.0002	0.0036	LOC105373416	uncharacterized LOC105373416
16907904	4.01	2.65	0.55	0.39	2.55	4.66E-05	0.001	LOC105373862	uncharacterized LOC105373862
16912965	5.11	3.76	0.55	0.33	2.55	6.22E-06	0.0002	TP53INP2	tumor protein p53 inducible nuclear protein 2
16729336	5.61	4.26	0.65	0.47	2.55	6.80E-05	0.0014	CAPN5	calpain 5
17072439	5.1	3.76	0.57	1.02	2.54	0.0018	0.0185	FER1L6	fer-1-like family member 6
16987528	6.98	5.63	0.35	0.61	2.54	5.08E-06	0.0002	LOC102724748	uncharacterized LOC102724748
16680034	3.31	1.96	0.72	0.5	2.54	5.32E-05	0.0011	RNF223	ring finger protein 223
16960807	8.2	6.86	0.41	0.11	2.54	4.61E-07	3.40E-05	CCNL1	cyclin L1
16807519	5.49	4.14	0.46	1.05	2.54	0.0004	0.0054	RHOV	ras homolog family member V
16666545	8.26	6.92	0.39	0.4	2.54	2.44E-06	0.0001	ADGRL2	adhesion G protein-coupled receptor L2
16938973	4.91	3.57	0.67	0.45	2.53	6.36E-05	0.0013	C3orf35	chromosome 3 open reading frame 35
16842147	3.07	1.73	0.91	0.45	2.53	0.0004	0.0058	CCDC144B	coiled-coil domain containing 144B (pseudogene)
16676498	2.54	1.2	1.39	0.26	2.53	0.0053	0.0425	MFSD4	major facilitator superfamily domain containing 4
17016787	4.81	3.48	0.73	0.36	2.53	0.0002	0.0027	RNF39	ring finger protein 39
17026593	4.81	3.48	0.73	0.36	2.53	0.0002	0.0027		
17031459	4.81	3.48	0.73	0.36	2.53	0.0002	0.0027		
17036198	4.81	3.48	0.73	0.36	2.53	0.0002	0.0027		
17041542	4.81	3.48	0.73	0.36	2.53	0.0002	0.0027		
16969711	3.35	2.01	0.85	0.74	2.53	0.0016	0.0164	RRH	retinal pigment epithelium-derived rhodopsin homolog
17017049	3.7	2.36	0.93	0.6	2.52	0.0002	0.0028	C6orf15	chromosome 6 open reading frame 15
17031716	3.7	2.36	0.93	0.6	2.52	0.0002	0.0028		
17034168	3.7	2.36	0.93	0.6	2.52	0.0002	0.0028		
17036418	3.7	2.36	0.93	0.6	2.52	0.0002	0.0028		
16762107	2.28	0.95	0.96	0.17	2.52	0.0005	0.0066	GYS2	glycogen synthase 2 (liver)
16872223	4.18	2.85	0.59	0.43	2.51	0.0001	0.0021		
17121266	4.12	2.79	0.99	0.51	2.51	0.0022	0.0217		
16821479	6.03	4.7	0.54	0.26	2.51	2.37E-05	0.0006	ATP2C2	ATPase, Ca++ transporting, type 2C, member 2
17061108	3.47	2.15	0.67	0.43	2.5	0.0003	0.0046	RASA4B; RASA4	RAS p21 protein activator 4B; RAS p21 protein activator 4
17118039	3.57	2.25	0.84	0.41	2.5	0.001	0.0112	LOC100507209	uncharacterized LOC100507209
17100420	3.89	2.57	0.42	0.51	2.49	1.30E-05	0.0004	NRARP	NOTCH-regulated ankyrin repeat protein
17010789	6.25	4.94	0.5	0.12	2.49	2.80E-06	0.0001	ZNF292	zinc finger protein 292
17050171	4.08	2.77	0.45	0.19	2.49	6.43E-07	4.27E-05	HBP1	HMG-box transcription factor 1
16697599	6.51	5.21	0.38	0.35	2.48	3.44E-07	2.80E-05	DENND1B	DENN/MADD domain containing 1B
17113053	2.89	1.59	0.63	0.32	2.47	1.96E-05	0.0005	LOC286437	uncharacterized LOC286437
16958786	3.99	2.69	0.58	0.28	2.47	8.20E-06	0.0003	MGLL	monoglyceride lipase
17009573	2.94	1.63	0.71	0.23	2.47	0.0003	0.0048	PAQR8	progestin and adipoQ receptor family member VIII
16776045	5.06	3.76	0.52	0.4	2.46	5.10E-05	0.0011		
16981848	2.65	1.35	0.77	0.49	2.46	0.0002	0.0026		
17096379	3.72	2.42	0.71	0.36	2.46	6.35E-05	0.0013	LOC105376171	uncharacterized LOC105376171
16875014	8.07	6.78	0.24	0.28	2.45	4.43E-09	1.48E-06	ZNF841; ZNF432	zinc finger protein 841; zinc finger protein 432
16660757	8.16	6.86	0.45	0.69	2.45	5.96E-05	0.0013	GRHL3	grainyhead-like transcription factor 3
17085635	3.09	1.8	0.3	0.31	2.45	1.13E-07	1.30E-05	PIP5K1B	phosphatidylinositol-4-phosphate 5-kinase, type I, beta
16871346	2.63	1.34	0.5	0.14	2.45	4.98E-07	3.57E-05	LOC101927522	uncharacterized LOC101927522
17122212	3.7	2.41	0.76	0.28	2.45	3.00E-05	0.0007		
17004657	2.11	0.82	1.06	0.24	2.45	0.002	0.0201	BMP6	bone morphogenetic protein 6
17069550	4.19	2.9	0.88	0.32	2.45	0.0002	0.0034	ADHFE1; C8orf46	alcohol dehydrogenase, iron containing 1; chromosome 8 open reading frame 46
17019484	6.12	4.83	0.21	0.13	2.45	4.32E-11	1.39E-07	GTPBP2	GTP binding protein 2
16841657	2.77	1.48	0.41	0.49	2.45	7.21E-05	0.0015		
16990379	3.72	2.43	0.86	0.31	2.45	0.0009	0.0107	PCDHGB8P	protocadherin gamma subfamily B, 8 pseudogene
17124318	2.09	0.8	0.96	0.21	2.45	0.0006	0.0082		
16839519	5.18	3.89	0.57	0.66	2.45	0.0001	0.002	TLCD2	TLC domain containing 2
17092512	7.92	6.63	0.49	0.33	2.44	6.44E-06	0.0002	ZDHHC21	zinc finger, DHHC-type containing 21
17000520	6.23	4.94	0.51	0.41	2.44	3.06E-06	0.0001	SNORD63	small nucleolar RNA, C/D box 63
16900084	4.4	3.12	0.35	0.84	2.43	0.0001	0.0023	IGKV1-5	immunoglobulin kappa variable 1-5
17123810	2.48	1.2	0.66	0.17	2.43	0.0002	0.003		
16920857	6.59	5.31	0.15	0.27	2.43	2.35E-09	1.05E-06	PPP1R3D	protein phosphatase 1, regulatory subunit 3D
16971966	6.17	4.89	0.54	0.38	2.43	3.40E-05	0.0008	FNIP2	folliculin interacting protein 2
17061117	2.46	1.18	1.03	0.49	2.43	0.0025	0.0234	RASA4B; RASA4	RAS p21 protein activator 4B; RAS p21 protein activator 4
16872990	4.34	3.06	0.31	0.29	2.42	4.94E-08	7.64E-06	PHLDB3	pleckstrin homology-like domain, family B, member 3
16977159	6.15	4.87	0.31	0.47	2.42	1.91E-06	9.38E-05	SOWAHB	sosondowah ankyrin repeat domain family member B

16767666	1.96	0.69	0.79	0.2	2.42	8.19E-05	0.0016	KCNC2	potassium channel, voltage gated Shaw related subfamily C, member 2
17065650	2.74	1.46	0.42	0.3	2.42	9.88E-07	5.87E-05		
17045592	2.61	1.33	0.35	0.27	2.42	4.95E-08	7.64E-06	LINC00957	long intergenic non-protein coding RNA 957
17118260	2.68	1.4	0.61	0.55	2.42	0.0001	0.0022	LOC84214	uncharacterized LOC84214
16660103	4.19	2.92	0.54	0.44	2.42	5.80E-05	0.0012	ARHGEF10L	Rho guanine nucleotide exchange factor 10 like
17092003	2.31	1.04	0.63	0.21	2.42	9.82E-05	0.0019	VLDLR-AS1	VLDLR antisense RNA 1
16925442	4.35	3.08	0.4	0.39	2.41	2.36E-05	0.0006	CBR3-AS1	CBR3 antisense RNA 1
16874778	2.17	0.9	1.24	0.31	2.41	0.0026	0.024	KLK14	kallikrein related peptidase 14
16670574	7.65	6.38	0.36	0.26	2.41	1.01E-07	1.20E-05	ECM1	extracellular matrix protein 1
17019190	6.98	5.72	0.47	0.26	2.41	4.87E-06	0.0002	C6orf132	chromosome 6 open reading frame 132
16915457	6.43	5.17	0.84	0.37	2.4	0.0021	0.0206	CDH26	cadherin 26
17121932	3.14	1.87	0.99	0.3	2.4	0.0004	0.0058		
16731068	2.53	1.27	0.57	0.42	2.4	5.68E-05	0.0012	LAYN	layilin
16903552	4.45	3.19	0.7	0.36	2.4	0.0001	0.0021	NEB	nebulin
16860691	6.76	5.5	0.27	0.16	2.39	2.77E-09	1.15E-06	KIAA0355	KIAA0355
16881838	7.96	6.71	0.34	0.1	2.38	1.54E-08	3.49E-06	HK2	hexokinase 2
17123530	2.21	0.96	0.66	0.15	2.38	5.04E-05	0.0011		
16985704	7.33	6.08	0.33	0.52	2.38	5.19E-06	0.0002	OCLN	occludin
16863820	7.88	6.63	0.32	0.74	2.38	4.11E-05	0.0009	SULT2B1	sulfotransferase family 2B member 1
16942202	7.15	5.9	0.42	0.32	2.38	3.09E-07	2.60E-05	PXK	PX domain containing serine/threonine kinase
16920169	8.52	7.28	0.51	0.35	2.38	1.28E-05	0.0004	B4GALT5	UDP-Gal:betaGlcNAc beta 1,4- galactosyltransferase, polypeptide 5
17096285	8.33	7.08	0.57	0.34	2.38	2.95E-05	0.0007	CTSV	cathepsin V
16803197	4.93	3.69	0.38	0.42	2.37	1.14E-06	6.49E-05	NEIL1	nei-like DNA glycosylase 1
17063221	2.01	0.77	0.79	0.15	2.37	0.0002	0.0029	FAM180A	family with sequence similarity 180, member A
16854594	6.96	5.72	0.51	0.51	2.37	3.19E-05	0.0008	GAREM1	GRB2 associated regulator of MAPK1 1
16849047	6.25	5.01	0.43	0.5	2.37	3.63E-06	0.0002	EVPL	envoplakin
17013279	2.83	1.59	0.4	0.31	2.37	2.81E-06	0.0001	STX11	syntaxin 11
16949062	5.03	3.79	0.29	0.8	2.36	7.02E-05	0.0014	EPHB3	EPH receptor B3
17124312	1.86	0.62	0.69	0.07	2.36	3.89E-05	0.0009		
17106051	7.89	6.66	0.3	0.15	2.36	1.04E-08	2.73E-06	MID2	midline 2
17038957	3.59	2.35	0.84	0.76	2.36	0.0035	0.0309		
16972750	6.26	5.02	0.51	0.27	2.36	1.42E-05	0.0004	CDKN2AIP	CDKN2A interacting protein
16757766	3.07	1.83	0.55	0.4	2.36	7.65E-06	0.0003	HSPB8	heat shock 22kDa protein 8
16729168	3.13	1.89	0.47	0.24	2.36	1.25E-06	6.94E-05	DGAT2	diacylglycerol O-acyltransferase 2
16971660	3.28	2.05	0.45	0.48	2.35	1.37E-05	0.0004	LRAT	lecithin retinol acyltransferase (phosphatidylcholine--retinol O-acyltransferase)
16907912	5.85	4.62	0.52	0.4	2.35	6.59E-05	0.0014	IKZF2	IKAROS family zinc finger 2
16810133	3.17	1.95	0.4	0.17	2.34	7.36E-08	9.84E-06	RORA	RAR-related orphan receptor A
16963094	3.85	2.62	0.41	0.25	2.34	4.06E-07	3.13E-05		
16801624	2.83	1.61	0.37	0.39	2.34	1.30E-06	7.15E-05		
16675034	2.14	0.92	0.8	0.68	2.34	0.0039	0.0334		
16926253	11.34	10.12	0.23	0.42	2.33	1.00E-06	5.90E-05	CSTB	cystatin B (stefin B)
17000518	3.66	2.43	0.52	0.51	2.33	0.0001	0.0021	HSPA9	heat shock 70kDa protein 9 (mortalin)
16847878	3.71	2.49	0.49	0.5	2.33	1.65E-05	0.0005	PLEKHM1P	pleckstrin homology domain containing, family M (with RUN domain) member 1 pseuc
17028680	4.37	3.16	0.75	0.3	2.33	0.0002	0.0037		
16786587	6.28	5.06	0.8	0.61	2.33	0.0048	0.0391	FOS	FBJ murine osteosarcoma viral oncogene homolog
17061127	6.89	5.68	0.71	1.15	2.32	0.0025	0.0235	RASA4B; RASA4	RAS p21 protein activator 4B; RAS p21 protein activator 4
17003180	4.96	3.74	0.46	0.42	2.32	2.56E-05	0.0007	RAB24; MXD3	RAB24, member RAS oncogene family; MAX dimerization protein 3
17097209	3.09	1.88	0.67	0.51	2.32	0.0019	0.0187		
17112607	8.5	7.28	0.58	0.19	2.32	7.48E-06	0.0003	TSPAN6	tetraspanin 6
16691214	7.85	6.64	0.2	0.42	2.32	5.49E-07	3.79E-05	DENND2C	DENN/MADD domain containing 2C
16707744	1.99	0.78	0.76	0.28	2.32	0.0003	0.0047	CYP2C19	cytochrome P450, family 2, subfamily C, polypeptide 19
16809549	2.31	1.09	1.13	0.3	2.32	0.0029	0.0264	WDR72	WD repeat domain 72
16690566	8.69	7.47	0.39	0.45	2.32	1.10E-05	0.0003	SORT1	sortilin 1
16972507	2.74	1.53	1.03	0.34	2.32	0.0002	0.0034	LOC102723627	uncharacterized LOC102723627
16916407	6.4	5.19	0.54	0.34	2.31	2.50E-05	0.0006	FKBP1A; SDCBP2;	FK506 binding protein 1A; syndecan binding protein (syntenin) 2; FKBP1A-SDCBP2 reac
17010604	3.33	2.12	0.91	0.57	2.31	0.002	0.0199		
16669278	3.15	1.94	1.06	0.38	2.31	0.0043	0.0358	FAM46C	family with sequence similarity 46, member C
16997636	2.86	1.65	0.47	0.67	2.31	0.0002	0.0037		
16885976	4.43	3.22	0.62	0.63	2.31	0.0008	0.0096		
16834176	3.48	2.28	0.84	0.39	2.31	0.0008	0.01		
16862959	3.17	1.96	0.43	0.3	2.31	3.61E-06	0.0002	LOC105372415	uncharacterized LOC105372415
16823169	3.81	2.6	0.76	0.54	2.31	0.0007	0.0089	PRSS27	protease, serine 27
16883647	4.6	3.4	0.52	0.47	2.31	0.0003	0.0045	IL1R1	interleukin 1 receptor, type I
16823263	3.42	2.22	0.32	0.37	2.31	1.31E-06	7.17E-05	CCDC64B	coiled-coil domain containing 64B
16844817	9.77	8.57	0.32	0.94	2.3	0.0002	0.0035	KRT16	keratin 16, type I
17083557	1.96	0.76	0.81	0.41	2.29	0.0021	0.0202	LOC105375969	uncharacterized LOC105375969
16884629	8.7	7.5	0.29	0.28	2.29	3.50E-08	5.92E-06	IL1RN	interleukin 1 receptor antagonist
16803642	2.65	1.46	0.53	0.36	2.29	5.45E-05	0.0012	KIAA1024	KIAA1024
16659904	3.59	2.39	0.72	0.46	2.29	0.0032	0.0286		
16840054	5.26	4.07	0.68	0.92	2.29	0.0033	0.0293	GGT6	gamma-glutamyltransferase 6
16939419	6.29	5.1	0.58	0.84	2.28	0.0004	0.0054		
17125022	3.23	2.04	0.45	0.23	2.28	7.05E-06	0.0003		
17125794	4.69	3.51	0.31	0.43	2.28	2.51E-06	0.0001		
16809872	2.34	1.15	0.6	0.72	2.27	0.0043	0.0363	LOC101928635	uncharacterized LOC101928635
16979133	7.18	6	0.29	0.35	2.27	1.22E-07	1.38E-05	CAMK2D	calcium/calmodulin-dependent protein kinase II delta
16877847	5.79	4.61	0.25	0.26	2.27	1.72E-08	3.70E-06		
16777794	8.08	6.89	0.36	0.15	2.27	8.22E-08	1.04E-05	UBL3	ubiquitin-like 3
17047738	4.53	3.34	0.65	0.52	2.27	0.0015	0.0155	LOC105375368	uncharacterized LOC105375368
16912127	7.24	6.06	0.53	0.79	2.27	0.0022	0.0213	LOC105372578	uncharacterized LOC105372578
17125034	7.93	6.75	0.47	0.25	2.27	2.01E-06	9.78E-05		

16678114	6.06	4.88	0.48	0.41	2.27	1.72E-05	0.0005	EPHX1	epoxide hydrolase 1, microsomal (xenobiotic)
17104201	3.48	2.3	0.65	0.33	2.26	0.002	0.0197	FAAH2	fatty acid amide hydrolase 2
16959575	4.64	3.46	0.54	0.37	2.26	5.53E-05	0.0012		
16676619	5.58	4.41	0.19	0.22	2.26	2.58E-09	1.10E-06	RASSF5	Ras association (RalGDS/AF-6) domain family member 5
16708796	4.52	3.35	0.44	0.49	2.26	5.20E-05	0.0011	INA	internexin neuronal intermediate filament protein, alpha
16713318	10.02	8.84	0.41	0.29	2.26	1.17E-06	6.61E-05		
16718232	4.46	3.29	0.34	0.24	2.26	2.86E-07	2.48E-05	ITPRIP	inositol 1,4,5-trisphosphate receptor interacting protein
16854486	10.95	9.77	0.25	0.44	2.26	1.50E-06	7.84E-05	DSC2	desmocollin 2
17024700	2.67	1.5	0.84	0.49	2.25	0.0021	0.0202	RAET1L	retinoic acid early transcript 1L
17033915	4.73	3.56	0.79	0.27	2.25	0.0004	0.0059		
17022588	6.52	5.35	0.18	0.37	2.25	1.57E-07	1.62E-05	CDK19	cyclin-dependent kinase 19
16963097	2.6	1.43	0.5	0.67	2.25	0.0004	0.0057		
17100679	8.37	7.2	0.59	0.22	2.25	1.30E-05	0.0004		
16666959	3.22	2.05	0.65	0.4	2.25	0.0002	0.0031	GBP1P1	guanylate binding protein 1, interferon-inducible pseudogene 1
16877007	5.97	4.8	0.43	0.23	2.24	1.07E-06	6.15E-05	KLF11	Kruppel-like factor 11
16790360	5.03	3.87	0.36	0.63	2.24	0.0004	0.0054	SNORD9	small nucleolar RNA, C/D box 9
16956285	2.87	1.7	0.41	0.3	2.24	5.85E-07	3.95E-05	PDZRN3	PDZ domain containing ring finger 3
16866065	5.46	4.3	0.31	0.58	2.23	7.42E-05	0.0015	ZNF773	zinc finger protein 773
16836624	7.56	6.4	0.39	0.44	2.23	1.32E-05	0.0004	MIR21; VMP1	microRNA 21; vacuole membrane protein 1
16792483	2.15	0.99	0.67	0.29	2.23	0.0002	0.003		
16908897	6.56	5.4	0.41	1.21	2.23	0.0017	0.0178	EPHA4	EPH receptor A4
16980470	2.88	1.73	0.64	0.33	2.23	0.0001	0.0019	NR3C2	nuclear receptor subfamily 3, group C, member 2
16824166	6.11	4.95	0.27	0.73	2.23	0.0004	0.0057	PKD1P6; NPIPP1	polycystic kidney disease 1 (autosomal dominant) pseudogene 6; nuclear pore comple [illegible]
16819689	2.73	1.57	0.72	0.38	2.23	0.0018	0.0185	NDRG4	NDRG family member 4
16765226	5.32	4.17	0.39	0.46	2.23	6.34E-06	0.0002	ITGB7	integrin beta 7
16929920	8.73	7.57	0.3	0.3	2.23	4.99E-07	3.58E-05	H1F0	H1 histone family, member 0
16774851	5.18	4.02	0.35	0.33	2.23	6.41E-07	4.26E-05	KCNRG; TRIM13	potassium channel regulator; tripartite motif containing 13
16747211	4.38	3.23	0.21	0.44	2.22	4.67E-07	3.42E-05	PLEKHG6	pleckstrin homology domain containing, family G (with RhoGef domain) member 6
16700806	7.43	6.28	0.29	0.34	2.22	9.58E-07	5.72E-05	LYST	lysosomal trafficking regulator
16711731	7.59	6.44	0.45	0.13	2.22	8.92E-07	5.47E-05	USP6NL	USP6 N-terminal like
16990312	1.58	0.43	0.59	0.27	2.22	5.39E-05	0.0012	PCDHB13	protocadherin beta 13
17096827	5.83	4.68	0.46	0.49	2.22	3.46E-05	0.0008	KLF4	Kruppel-like factor 4 (gut)
17104159	7.06	5.91	0.45	0.53	2.22	3.60E-05	0.0008	KLF8	Kruppel-like factor 8
16909162	3.46	2.31	0.56	0.55	2.22	0.0014	0.0153	LOC105373913	uncharacterized LOC105373913
16869506	4.3	3.15	0.49	0.4	2.21	8.02E-06	0.0003	PODNL1	podocan-like 1
16935455	3.53	2.38	0.4	0.17	2.21	2.98E-06	0.0001	PMM1	phosphomannomutase 1
16974482	2.33	1.19	0.49	0.26	2.21	1.50E-06	7.83E-05	LOC101929095	uncharacterized LOC101929095
16711750	2.92	1.78	0.75	0.45	2.21	0.001	0.0113	USP6NL	USP6 N-terminal like
16706499	7.49	6.35	0.53	0.4	2.21	0.0002	0.0026	PPIF	peptidylprolyl isomerase F
17052737	3.24	2.1	0.85	0.84	2.2	0.0048	0.0394	PIP	prolactin-induced protein
17021116	3.12	1.98	0.77	0.39	2.2	0.0028	0.0262	LOC102724168	uncharacterized LOC102724168
16782548	5.28	4.14	0.43	0.65	2.2	2.43E-05	0.0006	PCK2	phosphoenolpyruvate carboxykinase 2 (mitochondrial)
16910618	6.87	5.73	0.32	0.33	2.2	3.86E-06	0.0002	RBCK1	RanBP-type and C3HC4-type zinc finger containing 1
16828577	6.03	4.9	0.53	0.73	2.2	0.0037	0.0322	MAF	v-maf avian musculoaponeurotic fibrosarcoma oncogene homolog
16961598	2.35	1.21	0.71	0.27	2.2	7.80E-05	0.0016	PLD1	phospholipase D1, phosphatidylcholine-specific
17021217	9.6	8.47	0.49	0.24	2.19	1.97E-05	0.0005	ME1	malic enzyme 1, NADP(+)-dependent, cytosolic
17102639	2.51	1.37	0.61	0.36	2.19	0.0003	0.0047		
16873049	6.14	5.01	0.59	0.42	2.19	0.0002	0.0036	CADM4	cell adhesion molecule 4
16678143	4.17	3.04	0.86	0.48	2.19	0.0056	0.0441		
17013014	4.55	3.42	0.6	0.65	2.19	0.0005	0.0064	FLJ46906	uncharacterized LOC441172
16851078	5.33	4.2	0.78	0.57	2.19	0.0054	0.0434	LOC105371997	uncharacterized LOC105371997
17078626	5.43	4.3	0.54	0.61	2.19	0.0011	0.0119	SLC10A5	solute carrier family 10, member 5
16713652	1.9	0.77	0.38	0.27	2.19	1.70E-06	8.62E-05		
16714237	1.9	0.77	0.38	0.27	2.19	1.70E-06	8.62E-05		
16998293	4.62	3.5	0.33	0.37	2.18	2.31E-06	0.0001	GLRX	glutaredoxin
16838746	2.27	1.14	0.53	0.18	2.18	0.0001	0.0025	PP13	vegetative cell wall protein gp1
16911804	2.1	0.97	0.66	0.19	2.18	7.75E-05	0.0015	SLC24A3	solute carrier family 24 (sodium/potassium/calcium exchanger), member 3
17123310	5.11	3.98	0.81	0.39	2.18	0.0025	0.0235		
16917004	9.23	8.1	0.16	0.21	2.18	1.82E-09	8.76E-07	GPCPD1	glycerophosphocholine phosphodiesterase 1
17077135	7.96	6.83	0.29	0.1	2.18	8.17E-09	2.33E-06	RB1CC1	RB1-inducible coiled-coil 1
16703036	5.53	4.41	0.97	0.51	2.18	0.0047	0.0387	PLXDC2	plexin domain containing 2
16874978	4.19	3.07	0.23	0.35	2.18	8.82E-08	1.09E-05	ZNF350	zinc finger protein 350
17012281	6.26	5.14	0.22	0.28	2.18	1.36E-08	3.27E-06	TPD52L1	tumor protein D52-like 1
16991663	4.23	3.11	0.62	0.57	2.17	0.0003	0.0043	LOC105377682	uncharacterized LOC105377682
16962853	3.29	2.17	0.69	0.51	2.17	0.0008	0.0099	LOC105374285	uncharacterized LOC105374285
16727074	5.75	4.63	0.39	0.82	2.17	0.0022	0.0217	OVOL1	ovo-like zinc finger 1
16997279	3.48	2.36	0.46	0.38	2.17	1.40E-05	0.0004	LINC01336	long intergenic non-protein coding RNA 1336
17020823	6.5	5.38	0.28	0.2	2.17	1.29E-07	1.43E-05	SLC17A5	solute carrier family 17 (acidic sugar transporter), member 5
17087107	5.84	4.72	0.38	0.54	2.17	2.25E-06	0.0001	MIR23B; C9orf3	microRNA 23b; chromosome 9 open reading frame 3
16890207	5.7	4.59	0.47	0.65	2.17	0.0016	0.0163	MAP2	microtubule associated protein 2
17050120	2.09	0.98	0.64	0.53	2.17	0.0015	0.0159	LOC105375440	uncharacterized LOC105375440
16929615	6.19	5.07	0.33	0.41	2.16	1.83E-06	9.07E-05	APOL6	apolipoprotein L, 6
16718622	7.61	6.49	0.23	0.6	2.16	1.25E-05	0.0004	ABLIM1	actin binding LIM protein 1
17099463	4.69	3.57	0.82	0.28	2.16	0.0004	0.0051	ABO	ABO blood group (transferase A, alpha 1-3-N-acetylgalactosaminyltransferase; transfe [illegible]
16832880	4.41	3.29	0.49	0.36	2.16	2.12E-05	0.0006	ADAP2	ArfGAP with dual PH domains 2
16844137	6.73	5.62	0.19	0.32	2.16	1.42E-07	1.53E-05	FBXL20	F-box and leucine-rich repeat protein 20
16841660	6.47	5.36	0.28	0.77	2.16	0.0011	0.0121		
16674292	7.43	6.32	0.61	0.21	2.16	0.0005	0.0068	RALGPS2	Ral GEF with PH domain and SH3 binding motif 2
16860183	3.02	1.91	0.65	0.55	2.15	0.0007	0.0083	ZNF493	zinc finger protein 493
16712421	1.54	0.43	0.68	0.19	2.15	0.0002	0.0031	C10orf113	chromosome 10 open reading frame 113

17011450	3.19	2.09	0.71	0.47	2.15	0.0005	0.0072	FOXO3	forkhead box O3
17024356	3.75	2.64	0.52	0.42	2.15	5.57E-05	0.0012		
17086193	9.89	8.78	0.39	0.36	2.15	3.12E-06	0.0001	PSAT1	phosphoserine aminotransferase 1
16722684	7.34	6.24	0.31	0.2	2.15	2.76E-07	2.42E-05	ZDHHC13	zinc finger, DHHC-type containing 13
16904024	5.74	4.64	0.19	0.33	2.14	1.03E-06	6.03E-05	WDSUB1	WD repeat, sterile alpha motif and U-box domain containing 1
17123414	3.39	2.29	0.78	0.47	2.14	0.0008	0.0098		
16689400	1.65	0.55	0.89	0.22	2.14	0.0018	0.0185	GBP5	guanylate binding protein 5
16742422	2.22	1.12	0.96	0.31	2.14	0.0033	0.0291		
17074815	4.96	3.87	0.56	0.18	2.14	7.84E-06	0.0003	LONRF1	LON peptidase N-terminal domain and ring finger 1
16976502	6.15	5.05	0.42	0.36	2.14	9.01E-06	0.0003	YTHDC1	YTH domain containing 1
16873117	3.32	2.23	0.54	0.21	2.14	3.63E-05	0.0009	ZNF404	zinc finger protein 404
16997537	1.63	0.53	0.93	0.17	2.14	0.0005	0.0061	DMGDH	dimethylglycine dehydrogenase
16819892	9.58	8.48	0.21	0.53	2.14	2.81E-06	0.0001	CES2	carboxylesterase 2
16775434	7.72	6.63	0.33	0.32	2.13	1.24E-06	6.89E-05	LMO7	LIM domain 7
16959441	5.36	4.27	0.39	0.22	2.13	5.69E-07	3.87E-05	AMOTL2	angiomotin like 2
17059771	8.55	7.46	0.56	0.36	2.13	0.0001	0.0025	SAMD9	sterile alpha motif domain containing 9
17061106	4.94	3.85	0.68	0.42	2.13	0.0004	0.0058	RASA4B; RASA4	RAS p21 protein activator 4B; RAS p21 protein activator 4
16823666	9.25	8.16	0.34	0.39	2.13	8.78E-07	5.40E-05	PPL	periplakin
16949911	4.02	2.93	0.44	0.3	2.13	4.29E-05	0.001	LINC00969	long intergenic non-protein coding RNA 969
17055006	4.19	3.1	0.39	0.46	2.13	9.94E-05	0.0019	FAM220A	family with sequence similarity 220, member A
16976615	4.46	3.37	0.54	0.82	2.13	0.0003	0.0039	SULT1E1; SULT1D	sulfotransferase family 1E member 1; sulfotransferase family 1D member 1, pseudoge
16819099	7.88	6.8	0.41	0.51	2.13	0.0001	0.0021	CAPNS2	calpain, small subunit 2
17095587	2.56	1.47	0.37	0.13	2.13	2.24E-07	2.08E-05	SEMA4D	sema domain, immunoglobulin domain (Ig), transmembrane domain (TM) and short cy
17064600	4.05	2.96	0.51	0.27	2.13	1.88E-05	0.0005		
16779006	3.82	2.73	0.48	0.59	2.12	0.0003	0.0045	LPAR6	lysophosphatidic acid receptor 6
17014714	8.94	7.86	0.34	0.14	2.12	1.49E-07	1.57E-05	MLLT4	myeloid/lymphoid or mixed-lineage leukemia; translocated to, 4
17000854	7.55	6.47	0.38	0.19	2.12	4.48E-06	0.0002	TAF7	TAF7 RNA polymerase II, TATA box binding protein (TBP)-associated factor, 55kDa
16852858	8.83	7.74	0.5	0.33	2.12	0.0001	0.0025	SERPINB7	serpin peptidase inhibitor, clade B (ovalbumin), member 7
16990327	1.98	0.9	0.41	0.3	2.11	3.82E-05	0.0009	PCDHB18P	protocadherin beta 18 pseudogene
16829894	7.08	6	0.32	0.2	2.11	1.44E-07	1.54E-05	PLD2	phospholipase D2
16797985	1.88	0.81	1	0.31	2.11	0.0031	0.0281	PWRN1	Prader-Willi region non-protein coding RNA 1
17074004	3.43	2.36	0.22	0.34	2.11	9.21E-07	5.59E-05	LOC101927506	uncharacterized LOC101927506
16672052	2.41	1.33	0.84	0.24	2.11	0.0015	0.0154	RHBG	Rh family, B glycoprotein (gene/pseudogene)
17061153	3.1	2.03	0.48	0.22	2.1	2.92E-06	0.0001		
16698466	6.86	5.78	0.42	0.2	2.1	2.49E-06	0.0001	NUAK2	NUAK family, SNF1-like kinase, 2
16978624	1.86	0.78	0.44	0.41	2.1	0.0002	0.0034		
16934045	3.18	2.11	0.43	0.28	2.1	6.15E-06	0.0002	PIK3IP1	phosphoinositide-3-kinase interacting protein 1
16702524	6.93	5.86	0.65	0.74	2.1	0.0023	0.022	CAMK1D	calcium/calmodulin-dependent protein kinase ID
16886818	8.6	7.53	0.31	0.51	2.1	1.57E-05	0.0005	TANC1	tetratricopeptide repeat, ankyrin repeat and coiled-coil containing 1
16675924	5.29	4.22	0.53	0.7	2.1	0.0003	0.0044	ELF3	E74-like factor 3 (ets domain transcription factor, epithelial-specific)
16849635	5.9	4.83	0.43	0.47	2.09	3.70E-05	0.0009	CBX4	chromobox homolog 4
16850009	5.39	4.32	0.22	0.47	2.09	5.03E-06	0.0002	SIRT7	sirtuin 7
16679567	2.31	1.25	0.51	0.5	2.09	0.0002	0.0036		
16898326	9.02	7.96	0.15	0.42	2.09	2.32E-07	2.13E-05	PELI1	pellino E3 ubiquitin protein ligase 1
17075922	6.4	5.34	0.49	0.35	2.09	2.88E-05	0.0007	KIF13B	kinesin family member 13B
17004879	5.86	4.8	0.14	0.13	2.09	8.74E-11	1.83E-07	HIVEP1	human immunodeficiency virus type I enhancer binding protein 1
16847863	5.22	4.16	0.55	0.6	2.08	0.0007	0.0087	PLEKHM1P	pleckstrin homology domain containing, family M (with RUN domain) member 1 pseuc
16816010	3.27	2.21	0.52	0.27	2.08	0.0004	0.0061	MIR193BHG	MIR193B host gene
16820822	1.77	0.72	0.86	0.21	2.08	0.0005	0.0065	IL34	interleukin 34
16875949	2.71	1.65	0.52	0.51	2.08	0.0004	0.0056		
17095461	8.42	7.36	0.29	0.14	2.08	2.74E-07	2.41E-05	ZCCHC6	zinc finger, CCHC domain containing 6
16775491	2.31	1.26	0.63	0.32	2.08	0.0002	0.003	LMO7DN	LMO7 downstream neighbor
17015166	6.67	5.61	0.39	0.39	2.08	1.03E-05	0.0003	SLC22A23	solute carrier family 22, member 23
16809219	2.26	1.21	0.52	0.3	2.08	0.0002	0.0027	TNFAIP8L3	tumor necrosis factor, alpha-induced protein 8-like 3
16802064	2.95	1.9	0.54	0.2	2.08	4.03E-06	0.0002	ANKDD1A	ankyrin repeat and death domain containing 1A
17031422	6.38	5.33	0.76	0.38	2.07	0.0007	0.0089		
17033878	6.38	5.33	0.76	0.38	2.07	0.0007	0.0089		
17038920	6.38	5.33	0.76	0.38	2.07	0.0007	0.0089		
16819539	9.19	8.14	0.44	0.24	2.07	6.07E-06	0.0002	ADGRG1	adhesion G protein-coupled receptor G1
17088116	2.01	0.96	0.68	0.48	2.07	0.0001	0.0021	MIR4668	microRNA 4668
16784787	7.62	6.58	0.32	0.15	2.07	2.58E-07	2.30E-05	DAAM1	dishevelled associated activator of morphogenesis 1
17124530	5.43	4.38	0.2	0.25	2.07	2.25E-08	4.36E-06		
17121944	4.97	3.93	0.54	0.55	2.07	0.002	0.0195		
17119722	4.24	3.19	0.44	0.66	2.07	0.0001	0.0021		
16795612	1.93	0.88	0.71	0.2	2.06	0.0008	0.0096	EML5	echinoderm microtubule associated protein like 5
17078061	6.81	5.76	0.37	0.34	2.06	8.70E-06	0.0003	NCOA2	nuclear receptor coactivator 2
17060061	6.71	5.67	0.48	0.44	2.06	5.53E-05	0.0012	ASNS	asparagine synthetase (glutamine-hydrolyzing)
16789305	3.81	2.77	0.48	0.32	2.06	0.0002	0.0028	KLC1	kinesin light chain 1
16840961	3.05	2.01	0.85	0.47	2.06	0.0017	0.0178	LOC100128288	uncharacterized LOC100128288
16765404	5.46	4.42	0.32	0.27	2.06	5.62E-07	3.84E-05	CALCOCO1	calcium binding and coiled-coil domain 1
17102352	2.6	1.56	0.6	0.79	2.06	0.0049	0.0396	GK	glycerol kinase
16961551	6.97	5.93	0.2	0.76	2.06	6.44E-05	0.0013	PLD1	phospholipase D1, phosphatidylcholine-specific
17101896	6	4.96	0.28	0.18	2.06	1.20E-07	1.35E-05	CDKL5	cyclin-dependent kinase-like 5
16845899	4.66	3.62	0.3	0.28	2.06	1.19E-06	6.69E-05	ARHGAP27	Rho GTPase activating protein 27
17121428	2.13	1.09	0.9	0.19	2.05	0.0005	0.0062		
17057134	4.84	3.8	0.56	0.31	2.05	1.53E-05	0.0004	RASA4CP	RAS p21 protein activator 4C, pseudogene
17061121	3.62	2.58	0.8	0.69	2.05	0.0021	0.0206	RASA4B; RASA4	RAS p21 protein activator 4B; RAS p21 protein activator 4
16969414	6.93	5.89	0.28	0.25	2.05	7.84E-07	4.96E-05	TET2	tet methylcytosine dioxygenase 2
16977537	6.5	5.47	0.46	0.16	2.05	6.18E-06	0.0002	HPSE	heparanase
17063681	6.19	5.16	0.26	0.3	2.05	7.33E-07	4.72E-05	KIAA1147	KIAA1147

17070492	2.75	1.71	0.94	0.44	2.05	0.0034	0.0299	ATP6V0D2	ATPase, H+ transporting, lysosomal 38kDa, V0 subunit d2
16829784	2.51	1.48	0.48	0.55	2.05	0.0023	0.0222		
16964749	6.74	5.71	0.25	0.26	2.05	1.34E-07	1.46E-05	KIAA0232	KIAA0232
17123522	3.24	2.2	0.77	0.37	2.05	0.0006	0.0076		
16766940	5	3.97	0.83	0.39	2.05	0.0055	0.0434		
16873104	3.71	2.68	0.66	0.44	2.05	0.0007	0.0089	LYPD5	LY6/PLAUR domain containing 5
16775187	0.96	-0.08	0.92	0.24	2.05	0.0042	0.0356		
16672489	7.69	6.66	0.56	0.35	2.04	8.29E-05	0.0016	LINC01133	long intergenic non-protein coding RNA 1133
17116946	3.14	2.11	0.78	0.41	2.04	0.0039	0.0336	MAFIP	MAFF interacting protein (pseudogene)
16833965	3.72	2.7	0.33	0.51	2.04	0.0002	0.0029	THRA; NR1D1	thyroid hormone receptor, alpha; nuclear receptor subfamily 1, group D, member 1
17061138	8.78	7.75	0.52	0.58	2.04	0.0002	0.0034	UPK3BL; POLR2J2;	uroplakin 3B-like; polymerase (RNA) II (DNA directed) polypeptide J2; polymerase (RNA
16990897	4.71	3.68	0.29	0.52	2.04	0.0001	0.0019	AFAP1L1	actin filament associated protein 1-like 1
17121346	5.6	4.57	0.37	0.48	2.04	0.0001	0.0019		
17123970	2.16	1.14	0.79	0.26	2.03	0.0022	0.0214		
16696824	6.56	5.54	0.58	0.39	2.03	0.0006	0.0072	ABL2	ABL proto-oncogene 2, non-receptor tyrosine kinase
17024285	6.39	5.37	0.41	0.35	2.03	5.42E-05	0.0012	CITED2	Cbp/p300-interacting transactivator, with Glu/Asp rich carboxy-terminal domain, 2
16657572	5	3.98	0.46	0.25	2.03	2.28E-05	0.0006	PLEKHN1	pleckstrin homology domain containing, family N member 1
16721593	5.46	4.44	0.3	0.45	2.03	9.93E-06	0.0003	PPFIBP2	PTPRF interacting protein, binding protein 2 (liprin beta 2)
17076859	2.31	1.29	0.52	0.27	2.02	4.81E-05	0.0011		
16893697	6.34	5.32	0.37	0.39	2.02	1.46E-05	0.0004	LOC728323	uncharacterized LOC728323
16949505	2.51	1.5	0.41	0.32	2.02	0.0003	0.0045	TPRG1	tumor protein p63 regulated 1
16936640	4.87	3.86	0.46	0.59	2.02	0.0004	0.0061	LOC105373098	uncharacterized LOC105373098
16780917	6.01	5	0.27	0.47	2.02	1.59E-05	0.0005	IRS2	insulin receptor substrate 2
16840846	7.2	6.19	0.36	0.49	2.02	0.0005	0.0071	PER1	period circadian clock 1
16795908	4.93	3.92	0.32	0.47	2.02	2.64E-05	0.0007	CATSPERB	catsper channel auxiliary subunit beta
16990294	2.6	1.59	0.58	0.32	2.02	0.0001	0.0025	PCDHB10	protocadherin beta 10
16966008	8.39	7.38	0.27	0.33	2.02	1.89E-07	1.85E-05	KLF3	Kruppel-like factor 3 (basic)
16710020	6.19	5.18	0.27	0.3	2.01	5.05E-07	3.60E-05	TACC2	transforming, acidic coiled-coil containing protein 2
16911108	4.2	3.19	0.38	0.4	2.01	0.0009	0.0107	SMOX	spermine oxidase
17124532	3.8	2.79	0.46	0.27	2.01	2.89E-06	0.0001		
17045380	5.63	4.62	0.64	0.3	2.01	0.0002	0.0026	LINC00265	long intergenic non-protein coding RNA 265
17052935	5.57	4.56	0.78	0.61	2.01	0.0064	0.0492	OR2A9P; OR2A20I	olfactory receptor, family 2, subfamily A, member 9 pseudogene; olfactory receptor, fa
17126024	7.91	6.9	0.29	0.22	2.01	6.69E-07	4.41E-05		
16819325	7.84	6.83	0.29	0.37	2.01	1.81E-06	9.02E-05	HERPUD1	homocysteine-inducible, endoplasmic reticulum stress-inducible, ubiquitin-like domair
16872796	10.4	9.4	0.27	0.56	2	3.32E-05	0.0008	CXCL17	chemokine (C-X-C motif) ligand 17
16710360	9.03	8.03	0.2	0.11	2	1.73E-09	8.59E-07	ZRANB1	zinc finger, RAN-binding domain containing 1
16880669	9.1	8.1	0.43	0.38	2	9.17E-05	0.0018	LGALSL	lectin, galactoside-binding-like
16829664	2.41	3.41	0.24	0.38	-2	4.32E-06	0.0002	LOC105371489	uncharacterized LOC105371489
17067314	4.7	5.71	0.59	0.34	-2.01	0.0007	0.0089	SCARA3	scavenger receptor class A, member 3
16686557	2.73	3.74	0.27	0.39	-2.01	1.30E-05	0.0004	PIK3R3	phosphoinositide-3-kinase, regulatory subunit 3 (gamma)
17120506	7.56	8.57	0.2	0.3	-2.01	1.29E-07	1.43E-05		
17121840	3.76	4.77	0.37	0.45	-2.01	0.0002	0.0029		
17061662	5.33	6.34	0.48	0.87	-2.01	0.0013	0.014	LAMB1	laminin, beta 1
16829420	1.55	2.56	0.26	0.89	-2.01	0.0005	0.0062		
16757347	4.55	5.56	0.37	0.36	-2.01	0.0009	0.0104	OAS3; OAS2	2-5-oligoadenylate synthetase 3; 2-5-oligoadenylate synthetase 2
16866951	5.16	6.17	0.12	0.26	-2.01	2.19E-08	4.30E-06	LMNB2	lamin B2
17059974	6.1	7.11	0.38	0.17	-2.01	1.77E-06	8.83E-05	SLC25A13	solute carrier family 25 (aspartate/glutamate carrier), member 13
16987125	6.26	7.27	0.41	0.32	-2.01	8.97E-06	0.0003	POLR3G	polymerase (RNA) III (DNA directed) polypeptide G (32kD)
16851486	7.34	8.35	0.63	0.3	-2.02	0.0006	0.0079	LAMA3	laminin, alpha 3
16852206	3.54	4.56	0.28	0.29	-2.02	1.66E-06	8.46E-05	HAUS1	HAUS augmin like complex subunit 1
16849982	4.13	5.14	0.17	0.12	-2.02	5.74E-10	4.54E-07	ALYREF	Aly/REF export factor
17120866	7.15	8.17	0.49	0.22	-2.02	5.11E-05	0.0011		
17073078	1.64	2.66	0.3	0.35	-2.02	1.28E-05	0.0004	DENND3	DENN/MADD domain containing 3
17080747	1.86	2.88	0.48	0.65	-2.02	0.0027	0.025		
16981506	4.99	6.01	0.33	0.43	-2.02	4.50E-05	0.001	HMGB2	high mobility group box 2
16694617	3.7	4.72	0.7	0.5	-2.02	0.0008	0.0097	IQGAP3	IQ motif containing GTPase activating protein 3
16858970	4.83	5.85	0.74	0.52	-2.02	0.001	0.0116	ADGRE5	adhesion G protein-coupled receptor E5
16845158	6.36	7.38	0.33	0.21	-2.02	3.19E-07	2.65E-05	PTRF	polymerase I and transcript release factor
17070221	2.21	3.23	0.48	0.7	-2.02	0.0002	0.0034	PKIA	protein kinase (cAMP-dependent, catalytic) inhibitor alpha
17021845	4.83	5.85	0.18	0.29	-2.02	1.04E-07	1.22E-05	MMS22L	MMS22-like, DNA repair protein
17089898	6.7	7.72	0.24	0.15	-2.02	1.54E-08	3.49E-06	NUP188	nucleoporin 188kDa
16705934	4.49	5.51	0.46	0.33	-2.02	7.52E-06	0.0003	CHST3	carbohydrate (chondroitin 6) sulfotransferase 3
16755928	3.99	5.01	0.39	0.25	-2.02	1.03E-06	6.04E-05	PARPBP	PARP1 binding protein
17048083	2.21	3.23	0.37	0.42	-2.03	3.41E-05	0.0008	STEAP2	STEAP family member 2, metalloreductase
16935517	3.18	4.2	0.26	0.31	-2.03	1.24E-06	6.89E-05	CENPM	centromere protein M
16976211	7.31	8.33	0.34	0.24	-2.03	2.74E-06	0.0001	IGFBP7	insulin like growth factor binding protein 7
16938933	1.94	2.96	0.53	0.81	-2.03	0.0058	0.0457		
16717106	4.72	5.74	0.42	0.32	-2.03	0.0001	0.0024	RRP12	ribosomal RNA processing 12 homolog
17076063	7.39	8.41	0.39	0.24	-2.03	1.41E-05	0.0004	GSR	glutathione reductase
16835483	2.53	3.55	0.29	0.25	-2.03	1.65E-06	8.40E-05	ATP5G1	ATP synthase, H+ transporting, mitochondrial Fo complex subunit C1 (subunit 9)
16719802	3.16	4.18	0.38	0.63	-2.03	0.0005	0.0063	ADAM8	ADAM metallopeptidase domain 8
16733530	1.17	2.19	0.37	0.75	-2.03	0.0035	0.0311	LOC105369575	uncharacterized LOC105369575
17016043	5.37	6.39	0.34	0.28	-2.03	5.16E-06	0.0002	MBOAT1	membrane bound O-acyltransferase domain containing 1
17024079	5.78	6.8	0.33	0.27	-2.04	1.46E-05	0.0004	MAP3K5	mitogen-activated protein kinase kinase kinase 5
17092615	5.17	6.19	0.28	0.18	-2.04	6.34E-08	8.90E-06	PSIP1	PC4 and SFRS1 interacting protein 1
16657821	4.52	5.55	0.34	0.3	-2.04	2.28E-06	0.0001	ATAD3A; ATAD3B	ATPase family, AAA domain containing 3A; ATPase family, AAA domain containing 3B
17015240	2.28	3.31	0.43	0.36	-2.04	9.59E-06	0.0003	ECI2	enoyl-CoA delta isomerase 2
16733104	5.63	6.66	0.22	0.25	-2.04	2.47E-08	4.66E-06	CHEK1	checkpoint kinase 1
16678496	3.99	5.02	0.69	0.31	-2.04	0.0002	0.0031	HIST3H2BB	histone cluster 3, H2bb
16885432	7.01	8.04	0.46	0.11	-2.04	1.64E-05	0.0005	UGGT1	UDP-glucose glycoprotein glucosyltransferase 1

16691972	1.78	2.81	0.34	0.3	-2.04	3.82E-05	0.0009	PDE4DIP	phosphodiesterase 4D interacting protein
16927198	1.32	2.35	0.19	0.12	-2.04	1.63E-09	8.59E-07	RANBP1	RAN binding protein 1
17009862	4.07	5.1	0.16	0.15	-2.04	3.21E-10	3.46E-07	PRIM2	primase, DNA, polypeptide 2 (58kDa)
16674767	7.74	8.77	0.09	0.06	-2.05	1.27E-12	1.13E-08	DHX9	DEAH (Asp-Glu-Ala-His) box helicase 9
16765884	3.55	4.58	0.49	0.42	-2.05	0.0021	0.0206		
17062321	2.14	3.18	0.58	0.44	-2.05	0.0004	0.0054	CADPS2	Ca++-dependent secretion activator 2
17068782	7.3	8.33	0.19	0.19	-2.05	2.69E-09	1.13E-06	MCM4	minichromosome maintenance complex component 4
16985162	5.5	6.53	0.19	0.12	-2.05	9.33E-10	6.24E-07	IPO11; KIF2A	importin 11; kinesin heavy chain member 2A
16909413	3.65	4.69	0.51	0.47	-2.05	3.47E-05	0.0008	SP110	SP110 nuclear body protein
16753800	5.71	6.75	0.11	0.1	-2.05	1.96E-11	7.26E-08	NUP107	nucleoporin 107kDa
16885978	6.12	7.16	0.25	0.2	-2.05	1.15E-07	1.32E-05	R3HDM1	R3H domain containing 1
16699795	4.24	5.28	0.18	0.17	-2.06	1.49E-09	8.57E-07	NVL	nuclear VCP-like
17121916	2.79	3.83	0.43	0.45	-2.06	0.0002	0.0028		
16691969	4.37	5.41	0.5	0.37	-2.06	0.0003	0.0045	LOC105371220	uncharacterized LOC105371220
16757687	5.66	6.7	0.24	0.15	-2.06	2.91E-09	1.17E-06	RFC5	replication factor C subunit 5
17125762	1.49	2.53	0.37	0.36	-2.06	7.51E-06	0.0003		
17038792	4.16	5.2	0.5	0.35	-2.06	7.16E-06	0.0003		
16839131	5.61	6.65	0.35	0.17	-2.06	3.56E-07	2.87E-05	TBCD	tubulin folding cofactor D
16907960	4.97	6.02	0.41	0.14	-2.06	1.46E-06	7.70E-05	BARD1	BRCA1 associated RING domain 1
17106183	4.66	5.7	0.4	0.27	-2.06	5.44E-07	3.77E-05	TMEM164	transmembrane protein 164
16772144	4.43	5.48	0.32	0.1	-2.06	1.33E-07	1.46E-05	SCARB1	scavenger receptor class B, member 1
17121912	2.54	3.59	0.5	0.63	-2.07	0.0058	0.0453		
16702254	4.69	5.73	0.38	0.36	-2.07	4.64E-06	0.0002	LOC102723721	uncharacterized LOC102723721
17034090	4.23	5.28	0.24	0.1	-2.07	1.66E-09	8.59E-07		
16999379	5.32	6.37	0.47	0.38	-2.07	0.0001	0.0024	ALDH7A1	aldehyde dehydrogenase 7 family, member A1
16867432	4.57	5.63	0.61	0.32	-2.07	0.0002	0.0027	PTPRS	protein tyrosine phosphatase, receptor type, S
16904193	6.92	7.98	0.61	0.5	-2.07	0.0002	0.0035	ITGB6	integrin beta 6
16678579	6.36	7.41	0.37	0.18	-2.08	4.12E-07	3.16E-05	GALNT2	polypeptide N-acetylgalactosaminyltransferase 2
16933062	0.97	2.03	0.25	0.55	-2.08	9.16E-05	0.0018	DERL3	derlin 3
16725806	5.43	6.49	0.31	0.32	-2.08	3.80E-06	0.0002	INCENP	inner centromere protein
17083742	2.79	3.85	0.32	0.25	-2.08	1.92E-07	1.86E-05	CNTLN	centlein, centrosomal protein
16690843	5.85	6.9	0.31	0.11	-2.08	1.01E-07	1.20E-05	WDR77	WD repeat domain 77
16902945	3.29	4.35	0.55	0.17	-2.08	9.91E-06	0.0003	NCKAP5	NCK-associated protein 5
16970563	5.4	6.46	0.42	0.23	-2.08	3.33E-06	0.0001	PLK4	polo-like kinase 4
16677425	6.19	7.25	0.53	0.8	-2.09	0.0031	0.0284	CENPF	centromere protein F
16901830	5.77	6.83	0.37	0.28	-2.09	5.18E-06	0.0002	ANAPC1	anaphase promoting complex subunit 1
16819678	4.55	5.61	0.36	0.23	-2.09	1.36E-06	7.29E-05	GINS3	GINS complex subunit 3 (Psf3 homolog)
16776160	1.27	2.33	0.29	0.71	-2.09	0.0001	0.0021	ITGBL1	integrin beta like 1
16830235	3.1	4.16	0.57	0.6	-2.09	0.0011	0.0123	ALOX12P2	arachidonate 12-lipoxygenase pseudogene 2
17005871	1.53	2.6	0.3	0.57	-2.09	0.0007	0.0083	HIST1H2BN	histone cluster 1, H2bn
16799426	5.26	6.33	0.19	0.41	-2.09	1.93E-06	9.46E-05	PAK6; BUB1B	p21 protein (Cdc42/Rac)-activated kinase 6; BUB1 mitotic checkpoint serine/threonine
17113525	0.91	1.97	0.19	0.23	-2.09	1.87E-08	3.88E-06	KLHL13	kelch-like family member 13
16791086	4.46	5.52	0.29	0.24	-2.09	1.43E-07	1.53E-05	IPO4	importin 4
16823229	1.52	2.58	0.35	0.39	-2.09	1.62E-05	0.0005	PKMYT1	protein kinase, membrane associated tyrosine/threonine 1
16777896	7.47	8.53	0.18	0.24	-2.09	7.25E-09	2.13E-06	HSPH1	heat shock 105kDa/110kDa protein 1
16735873	4.38	5.45	0.25	0.27	-2.1	1.54E-06	8.00E-05	GALNT18	polypeptide N-acetylgalactosaminyltransferase 18
16908948	8.11	9.18	0.28	0.14	-2.1	5.18E-09	1.66E-06	FARSB	phenylalanyl-tRNA synthetase beta subunit
17085190	2.36	3.44	0.6	0.58	-2.1	0.0052	0.042		
17085457	2.36	3.44	0.6	0.58	-2.1	0.0052	0.042		
17094376	2.36	3.44	0.6	0.58	-2.1	0.0052	0.042		
16977052	0.71	1.78	0.32	0.72	-2.1	0.0023	0.0224	CXCL10	chemokine (C-X-C motif) ligand 10
16811073	4.26	5.33	0.33	0.32	-2.1	3.30E-07	2.72E-05	CLN6	ceroid-lipofuscinosis, neuronal 6, late infantile, variant
17016386	2.16	3.23	0.25	0.44	-2.1	1.33E-05	0.0004	HIST1H3D; HIST1H	histone cluster 1, H3d; histone cluster 1, H2ad
16687188	4.28	5.36	0.25	0.23	-2.1	1.05E-08	2.74E-06	ORC1	origin recognition complex subunit 1
16805422	3.41	4.48	0.31	0.31	-2.11	2.84E-06	0.0001	NR2F2	nuclear receptor subfamily 2, group F, member 2
17111998	1.59	2.66	0.26	0.28	-2.11	4.30E-07	3.26E-05	ERCC6L	excision repair cross-complementation group 6-like
16730467	2.14	3.22	0.12	0.57	-2.11	0.0001	0.0024	TMEM133	transmembrane protein 133
16771067	4.38	5.46	0.46	0.43	-2.11	0.0001	0.002	CIT; MIR1178	citron rho-interacting serine/threonine kinase; microRNA 1178
17067696	4.77	5.85	0.23	0.69	-2.11	0.0005	0.0071	NRG1	neuregulin 1
16753670	2.17	3.25	0.63	0.52	-2.11	0.0025	0.0237	IRAK3	interleukin 1 receptor associated kinase 3
16899799	4.1	5.18	0.36	0.67	-2.11	0.0019	0.0194	POLR1A	polymerase (RNA) I polypeptide A
16792381	4.69	5.77	0.11	0.33	-2.11	1.51E-07	1.59E-05	MIS18BP1	MIS18 binding protein 1
16845349	4.34	5.42	0.19	0.16	-2.12	9.27E-10	6.24E-07	BRCA1	breast cancer 1, early onset
17123164	2.99	4.07	0.87	0.37	-2.12	0.0023	0.0221		
16664218	6.19	7.27	0.53	0.45	-2.12	2.16E-05	0.0006	TSPAN1	tetraspanin 1
17093031	5.19	6.28	0.36	0.34	-2.12	2.29E-06	0.0001	MOB3B	MOB kinase activator 3B
17075553	1.01	2.1	0.54	1.33	-2.12	0.0015	0.0159	STC1	stanniocalcin 1
16707221	6.67	7.76	0.32	0.37	-2.12	3.16E-07	2.63E-05	KIF20B	kinesin family member 20B
16910501	3.76	4.84	0.44	0.35	-2.12	8.38E-06	0.0003	DTYMK	deoxythymidylate kinase
16862439	5.51	6.59	0.37	0.62	-2.13	0.0001	0.0022	AXL	AXL receptor tyrosine kinase
16967831	4.58	5.67	0.45	0.65	-2.13	0.0034	0.0302	EPGN	epithelial mitogen
16803754	5.39	6.48	0.33	0.42	-2.13	1.03E-05	0.0003	CEMIP	cell migration inducing protein, hyaluronan binding
16832429	4.53	5.62	0.39	0.35	-2.13	2.65E-06	0.0001	TMEM97	transmembrane protein 97
16929573	5.33	6.42	0.25	0.3	-2.13	1.58E-07	1.63E-05	MCM5	minichromosome maintenance complex component 5
16677071	2.95	4.04	0.46	0.56	-2.13	0.0002	0.0028	SERTAD4	SERTA domain containing 4
16849576	6.34	7.43	0.53	0.35	-2.13	0.0002	0.0032	LGALS3BP	lectin, galactoside-binding, soluble, 3 binding protein
16873239	1.71	2.8	0.47	0.62	-2.13	0.0011	0.0124	TRAPPC6A	trafficking protein particle complex 6A
17125218	4.37	5.46	0.41	0.31	-2.13	4.22E-06	0.0002		
17090713	2.6	3.69	0.51	0.7	-2.13	0.0026	0.0245	CEL	carboxyl ester lipase
16890602	4.29	5.39	0.67	0.4	-2.14	0.0002	0.003	PKI55	DKFZp434H1419

17109503	4.81	5.91	0.46	0.23	-2.14	2.34E-05	0.0006	PHKA2	phosphorylase kinase, alpha 2 (liver)
16856910	6.23	7.33	0.27	0.16	-2.14	1.23E-08	3.10E-06	NCLN	nicalin
16756212	2.98	4.07	0.48	0.64	-2.14	0.0008	0.0098	CHST11	carbohydrate (chondroitin 4) sulfotransferase 11
16768936	2.88	3.98	0.16	0.39	-2.14	6.99E-07	4.55E-05	TMPO-AS1	TMPO antisense RNA 1
16783836	3.46	4.55	0.3	0.27	-2.14	6.58E-07	4.36E-05	FANCM	Fanconi anemia complementation group M
17087716	6.65	7.75	0.41	0.22	-2.14	3.42E-07	2.80E-05	SMC2	structural maintenance of chromosomes 2
17020044	6.36	7.46	0.41	0.4	-2.14	7.33E-06	0.0003	TRAM2	translocation associated membrane protein 2
16798820	0.79	1.89	0.34	0.99	-2.14	0.0008	0.0098		
16840766	2.33	3.43	0.45	0.27	-2.14	0.0001	0.0024	LOC100128281	uncharacterized LOC100128281
17016494	9.88	10.98	0.44	0.23	-2.15	8.45E-07	5.24E-05	HIST1H4K; HIST1H	histone cluster 1, H4k; histone cluster 1, H4j
17039133	4.25	5.35	0.26	0.13	-2.15	1.84E-09	8.76E-07		
16671457	5.89	6.99	0.42	0.25	-2.15	6.87E-06	0.0002	IL6R	interleukin 6 receptor
16873848	5.04	6.14	0.43	0.19	-2.15	1.56E-06	8.07E-05	LIG1	ligase I, DNA, ATP-dependent
17041700	4.18	5.28	0.24	0.09	-2.15	7.79E-10	5.60E-07		
16861318	2.46	3.57	0.16	0.35	-2.15	2.33E-07	2.13E-05	WDR62	WD repeat domain 62
16897797	6.82	7.92	0.32	0.22	-2.15	2.08E-07	1.97E-05	PNPT1	polyribonucleotide nucleotidyltransferase 1
16889054	2.57	3.68	0.26	0.52	-2.16	6.17E-05	0.0013	CCDC150	coiled-coil domain containing 150
16676526	0.96	2.07	0.3	0.69	-2.16	0.0001	0.0024	FAM72A	family with sequence similarity 72, member A
17107045	5.68	6.79	0.3	0.16	-2.16	4.98E-08	7.64E-06	HPRT1	hypoxanthine phosphoribosyltransferase 1
16804629	1.38	2.49	0.37	0.56	-2.16	0.0001	0.0025		
17033890	2	3.11	0.66	0.41	-2.16	0.0004	0.0059		
17038932	2	3.11	0.66	0.41	-2.16	0.0004	0.0059		
17069545	4.18	5.29	0.6	0.45	-2.16	0.0001	0.0024	RRS1	ribosome biogenesis regulator homolog
16683377	2.33	3.45	0.48	0.61	-2.16	0.0007	0.0085	ID3	inhibitor of DNA binding 3, dominant negative helix-loop-helix protein
16777401	4.94	6.05	0.41	0.26	-2.16	2.85E-06	0.0001	MIPEP	mitochondrial intermediate peptidase
16843417	2.57	3.68	0.27	0.6	-2.16	3.58E-05	0.0008	SLFN12	schlafen family member 12
17117588	6.45	7.57	0.22	0.14	-2.16	2.28E-09	1.03E-06		
16836457	4.39	5.5	0.37	0.18	-2.17	3.49E-07	2.83E-05	RAD51C	RAD51 paralog C
17083357	3.9	5.02	0.68	0.44	-2.17	0.0003	0.0041	CD274	CD274 molecule
16835781	3.48	4.6	0.45	0.22	-2.17	2.13E-05	0.0006	XYLT2	xylosyltransferase II
16970409	5.27	6.38	0.42	0.11	-2.17	4.43E-07	3.33E-05	SPATA5	spermatogenesis associated 5
16878890	5.82	6.93	0.18	0.22	-2.17	2.99E-09	1.18E-06	TTC27	tetratricopeptide repeat domain 27
16834196	5.1	6.22	0.41	0.25	-2.17	3.55E-05	0.0008	FKBP10	FK506 binding protein 10
17097914	3.7	4.82	0.34	0.48	-2.17	1.85E-05	0.0005	PHF19	PHD finger protein 19
16777502	4.41	5.53	0.17	0.31	-2.17	7.06E-08	9.52E-06	CENPJ	centromere protein J
16995409	7.32	8.44	0.17	0.15	-2.18	3.73E-10	3.52E-07	NUP155	nucleoporin 155kDa
17103413	3.88	5.01	0.4	0.15	-2.18	2.38E-07	2.17E-05	SUV39H1	suppressor of variegation 3-9 homolog 1 (Drosophila)
16997662	1.3	2.42	0.3	0.43	-2.18	1.67E-06	8.46E-05	DHFR	dihydrofolate reductase
17084184	6.81	7.94	0.36	0.16	-2.18	2.14E-07	2.01E-05	ACO1	aconitase 1, soluble
16920548	3.1	4.23	0.2	0.51	-2.19	8.62E-06	0.0003	AURKA	aurora kinase A
17001800	1.57	2.7	0.34	1.1	-2.19	0.0016	0.0165	ANXA6	annexin A6
16959985	5.11	6.24	0.4	0.16	-2.19	4.67E-06	0.0002	ATR	ATR serine/threonine kinase
17083915	5.84	6.97	0.34	0.12	-2.19	2.11E-07	1.99E-05	FOCAD	focadhesin
16884523	6.6	7.73	0.4	0.53	-2.19	2.42E-05	0.0006	SLC20A1	solute carrier family 20 (phosphate transporter), member 1
16819213	3.52	4.65	0.81	0.68	-2.19	0.0008	0.0097	MT1L; MT1M	metallothionein 1L (gene/pseudogene); metallothionein 1M
16948021	6.73	7.86	0.22	0.26	-2.2	2.52E-08	4.67E-06	ECT2	epithelial cell transforming 2
16820537	7.7	8.83	0.28	0.19	-2.2	4.99E-08	7.64E-06	UTP4	UTP4 small subunit (SSU) processome component
16925239	4.68	5.82	0.31	0.23	-2.2	2.80E-07	2.44E-05	DONSON	downstream neighbor of SON
16725735	4.43	5.57	0.2	0.32	-2.2	2.49E-08	4.66E-06	FEN1; FADS2	flap structure-specific endonuclease 1; fatty acid desaturase 2
16842812	4.94	6.08	0.19	0.25	-2.21	1.41E-08	3.32E-06	TLCD1	TLC domain containing 1
17019805	7.31	8.45	0.52	0.29	-2.21	4.96E-05	0.0011	TNFRSF21	tumor necrosis factor receptor superfamily, member 21
16748449	4.94	6.08	0.33	0.27	-2.21	1.87E-07	1.83E-05	MIR1244-1; MIR12	microRNA 1244-1; microRNA 1244-2; microRNA 1244-3; microRNA 1244-4
16761116	4.94	6.08	0.33	0.27	-2.21	1.87E-07	1.83E-05	MIR1244-1; MIR12	microRNA 1244-1; microRNA 1244-2; microRNA 1244-3; microRNA 1244-4
16988297	4.94	6.08	0.33	0.27	-2.21	1.87E-07	1.83E-05	MIR1244-1; MIR12	microRNA 1244-1; microRNA 1244-2; microRNA 1244-3; microRNA 1244-4
16687418	7.34	8.49	0.23	0.09	-2.21	4.55E-10	3.88E-07	NDC1	NDC1 transmembrane nucleoporin
17026706	4.02	5.17	0.22	0.1	-2.21	2.27E-10	2.82E-07		
17031635	4.02	5.17	0.22	0.1	-2.21	2.27E-10	2.82E-07		
16949562	1.91	3.06	0.47	0.62	-2.22	0.0017	0.017	MIR944	microRNA 944
17120002	2.04	3.19	0.33	0.26	-2.22	1.39E-07	1.51E-05		
16870925	2.96	4.11	0.39	0.57	-2.22	0.0002	0.0031	ZNF724P	zinc finger protein 724, pseudogene
16691046	2.98	4.14	0.46	0.3	-2.22	6.83E-06	0.0002	PHTF1	putative homeodomain transcription factor 1
16938899	4.91	6.06	0.21	0.08	-2.22	2.10E-10	2.81E-07	MLH1	mutL homolog 1
17126286	4.52	5.68	0.95	0.53	-2.23	0.003	0.0275		
16890935	1.56	2.72	0.33	0.62	-2.23	0.0002	0.0038	PLCD4	phospholipase C, delta 4
16918074	4.67	5.83	0.54	0.43	-2.23	0.0003	0.0046	LOC105372580	uncharacterized LOC105372580
17031434	2.02	3.18	0.77	0.49	-2.23	0.0008	0.0096		
16702257	5.43	6.59	0.74	0.47	-2.24	0.0002	0.0034	LINC00707	long intergenic non-protein coding RNA 707
16785316	7.31	8.47	0.34	0.18	-2.24	1.03E-07	1.21E-05	MTHFD1	methylenetetrahydrofolate dehydrogenase (NADP+ dependent) 1, methenyltetrahydr
17028857	4.16	5.32	0.24	0.09	-2.24	4.14E-10	3.76E-07		
17036357	4.16	5.32	0.24	0.09	-2.24	4.14E-10	3.76E-07		
16798136	2.62	3.78	0.47	0.39	-2.24	3.63E-06	0.0002	SNRPN	small nuclear ribonucleoprotein polypeptide N
16888143	2.94	4.1	0.58	0.33	-2.25	0.0001	0.0022	LOC101927027	uncharacterized LOC101927027
17104947	4.34	5.51	0.46	0.27	-2.25	8.98E-06	0.0003	SLC16A2	solute carrier family 16, member 2 (thyroid hormone transporter)
16728785	2.93	4.1	0.92	0.7	-2.25	0.0048	0.0393	LOC105369382	uncharacterized LOC105369382
16942648	2.01	3.18	0.52	0.49	-2.25	7.57E-05	0.0015	GXYLT2	glucoside xylosyltransferase 2
17027743	1.39	2.56	0.31	0.72	-2.25	0.0002	0.0034		
16799793	6.83	8.01	0.62	0.39	-2.26	4.57E-06	0.0002	NUSAP1	nucleolar and spindle associated protein 1
16670383	9.85	11.03	0.27	0.23	-2.26	7.66E-08	9.96E-06	HIST2H2AA4; HIST	histone cluster 2, H2aa4; histone cluster 2, H2aa3
16856812	4.03	5.21	0.17	0.17	-2.27	6.33E-10	4.77E-07	THOP1	thimet oligopeptidase 1
17123142	4.98	6.16	0.36	0.24	-2.27	3.26E-07	2.70E-05		

16852445	5.17	6.35	0.34	0.34	-2.27	4.55E-07	3.39E-05	C18orf54	chromosome 18 open reading frame 54
16850477	6.7	7.88	0.37	0.29	-2.27	1.02E-06	6.00E-05	TYMS	thymidylate synthetase
16834091	3.42	4.6	0.6	0.64	-2.27	0.0026	0.0244	IGFBP4	insulin like growth factor binding protein 4
16729557	2.36	3.55	0.25	0.52	-2.27	1.82E-05	0.0005	DDIAS	DNA damage-induced apoptosis suppressor
16913486	2.1	3.28	0.55	0.35	-2.27	9.06E-05	0.0017	VSTM2L	V-set and transmembrane domain containing 2 like
16995938	3.7	4.88	0.38	0.49	-2.27	1.94E-05	0.0005	C5orf34	chromosome 5 open reading frame 34
16681611	7.91	9.1	0.44	0.23	-2.27	8.70E-07	5.36E-05	SRM	spermidine synthase
16955939	4.23	5.41	0.26	0.54	-2.27	6.09E-06	0.0002	LRIG1	leucine-rich repeats and immunoglobulin-like domains 1
17016360	1.17	2.36	0.29	0.46	-2.28	2.54E-06	0.0001	HIST1H4B	histone cluster 1, H4b
16657598	4.69	5.88	0.31	0.27	-2.28	2.29E-07	2.11E-05	AGRN	agrin
16760048	5.2	6.39	0.27	0.33	-2.28	8.99E-08	1.10E-05	FOXM1	forkhead box M1
16774303	3.75	4.94	1.04	0.43	-2.28	0.0028	0.0259	RGCC	regulator of cell cycle
17085082	4.66	5.85	0.53	0.36	-2.28	6.91E-06	0.0002	ALDH1B1	aldehyde dehydrogenase 1 family, member B1
16745139	1.64	2.83	0.27	0.5	-2.29	5.91E-06	0.0002		
17005586	3.12	4.31	0.25	0.32	-2.29	8.58E-08	1.07E-05	HIST1H4E	histone cluster 1, H4e
16989265	3.17	4.36	0.21	0.54	-2.29	1.13E-05	0.0004	JADE2	jade family PHD finger 2
16780322	2.04	3.24	0.3	0.65	-2.29	9.55E-05	0.0018	DZIP1	DAZ interacting zinc finger protein 1
17010552	5.44	6.63	0.51	0.49	-2.29	8.71E-06	0.0003	TTK	TTK protein kinase
16833345	8.75	9.95	0.27	0.2	-2.3	3.78E-08	6.33E-06	AP2B1	adaptor-related protein complex 2, beta 1 subunit
16689734	4.89	6.09	0.72	0.96	-2.3	0.0001	0.0025	MIG7	mig-7
16697544	5.92	7.12	0.53	0.63	-2.3	9.64E-05	0.0018	ASPM	abnormal spindle microtubule assembly
17101587	0.75	1.95	0.37	0.42	-2.3	1.35E-05	0.0004	EGFL6	EGF-like-domain, multiple 6
16980974	1.72	2.92	0.49	0.49	-2.3	7.36E-05	0.0015	FAM198B	family with sequence similarity 198, member B
16666509	1.89	3.09	0.39	0.79	-2.3	0.0002	0.0034	IFI44	interferon-induced protein 44
16798919	6.38	7.58	0.4	0.37	-2.3	2.71E-06	0.0001	ARHGAP11A	Rho GTPase activating protein 11A
16951756	6.68	7.89	0.34	0.39	-2.3	6.13E-06	0.0002	SLC4A7	solute carrier family 4, sodium bicarbonate cotransporter, member 7
16730429	4.87	6.08	0.5	0.44	-2.31	2.86E-05	0.0007	ARHGAP42	Rho GTPase activating protein 42
16959505	0.82	2.02	0.27	0.73	-2.31	0.0002	0.0034		
17011005	2.56	3.77	0.29	0.44	-2.31	2.10E-06	0.0001		
16863104	6.19	7.4	0.43	0.25	-2.31	7.91E-07	4.99E-05	TOMM40	translocase of outer mitochondrial membrane 40 homolog (yeast)
16714998	4.18	5.4	0.34	0.12	-2.32	4.36E-08	7.05E-06	DNA2	DNA replication helicase/nuclease 2
16820483	2.98	4.2	0.45	0.4	-2.32	4.15E-06	0.0002	CDH3	cadherin 3, type 1, P-cadherin (placental)
17010354	8.33	9.54	0.83	0.47	-2.32	0.0052	0.0415	CD109	CD109 molecule
17048879	5.57	6.78	0.38	0.28	-2.32	3.09E-06	0.0001	ARPC1B; ARPC1A	actin related protein 2/3 complex subunit 1B; actin related protein 2/3 complex subun
17084904	6.53	7.74	0.31	0.19	-2.32	1.69E-08	3.69E-06	MELK	maternal embryonic leucine zipper kinase
16679936	4.83	6.05	0.73	0.83	-2.32	0.0013	0.0143		
16748835	1.78	3	0.2	0.65	-2.33	9.54E-06	0.0003	PIK3C2G	phosphatidylinositol-4-phosphate 3-kinase, catalytic subunit type 2 gamma
17065047	4.24	5.46	0.5	0.57	-2.33	6.27E-05	0.0013	LOC389831; LOC1	uncharacterized LOC389831; uncharacterized LOC105379498; uncharacterized LOC10
16720077	4.86	6.08	1.03	0.87	-2.33	0.0023	0.0222	IFITM2	interferon induced transmembrane protein 2
17080935	1.36	2.59	0.58	0.68	-2.34	0.0004	0.0051	LOC105375743	uncharacterized LOC105375743
16897834	8.12	9.34	0.69	0.24	-2.34	7.22E-05	0.0015	EFEMP1	EGF containing fibulin-like extracellular matrix protein 1
16775324	4.64	5.87	0.45	0.61	-2.34	8.21E-05	0.0016	BORA	bora, aurora kinase A activator
17005593	5.59	6.82	0.42	0.43	-2.34	2.26E-06	0.0001	HIST1H3E	histone cluster 1, H3e
16937498	2.71	3.93	0.41	0.35	-2.34	2.03E-06	9.87E-05	PRRT3-AS1	PRRT3 antisense RNA 1
17066921	1.92	3.15	0.49	0.59	-2.34	2.88E-05	0.0007	ADAM28	ADAM metallopeptidase domain 28
16780640	3.83	5.06	0.71	0.15	-2.34	7.37E-05	0.0015	LOC105370333	uncharacterized LOC105370333
16926942	4.27	5.5	0.87	0.43	-2.35	0.0006	0.0075	USP18	ubiquitin specific peptidase 18
16893846	2.35	3.58	0.52	0.81	-2.35	0.0009	0.0101	PXDN	peroxidasin
17080749	5.88	7.11	0.24	0.22	-2.35	1.57E-09	8.59E-07	ATAD2	ATPase family, AAA domain containing 2
16712576	2.37	3.61	0.3	0.42	-2.35	5.53E-07	3.81E-05	PRTFDC1	phosphoribosyl transferase domain containing 1
16857258	3.87	5.11	0.43	0.41	-2.36	9.19E-07	5.59E-05	UHRF1	ubiquitin-like with PHD and ring finger domains 1
16837270	9.11	10.34	0.29	0.43	-2.36	5.03E-07	3.59E-05	KPNA2	karyopherin alpha 2 (RAG cohort 1, importin alpha 1)
16735861	1.93	3.17	0.35	0.25	-2.36	8.52E-08	1.07E-05		
16692583	3.28	4.52	0.33	0.72	-2.36	0.0001	0.002		
16819269	6.05	7.29	0.22	0.11	-2.36	6.15E-10	4.77E-07	NUP93	nucleoporin 93kDa
16688386	4.91	6.15	0.34	0.61	-2.36	1.12E-05	0.0004	DEPDC1	DEP domain containing 1
16664118	5.65	6.89	0.22	0.12	-2.36	1.34E-10	2.22E-07	NASP	nuclear autoantigenic sperm protein (histone-binding)
16832852	5.1	6.34	0.27	0.11	-2.37	3.54E-10	3.52E-07	ATAD5	ATPase family, AAA domain containing 5
16896196	4.36	5.6	0.88	0.83	-2.37	0.0045	0.0375	XDH	xanthine dehydrogenase
17012379	5.3	6.54	0.44	0.32	-2.37	1.33E-06	7.23E-05	CENPW	centromere protein W
16774235	2.66	3.91	0.4	0.26	-2.37	2.13E-06	0.0001	SLC25A15; MIR62	solute carrier family 25 (mitochondrial carrier; ornithine transporter) member 15; micr
16969495	2.98	4.22	0.36	0.59	-2.37	0.0007	0.0089	NPNT	nephronectin
17059828	7.98	9.23	0.6	0.73	-2.37	8.77E-05	0.0017	TFPI2	tissue factor pathway inhibitor 2
17093260	4.42	5.67	0.44	0.37	-2.37	2.68E-06	0.0001	NOL6	nucleolar protein 6 (RNA-associated)
16821239	5.32	6.56	0.26	0.18	-2.37	3.62E-09	1.30E-06	CENPN	centromere protein N
16661192	2.25	3.5	0.62	0.42	-2.38	4.75E-05	0.0011	HMGN2	high mobility group nucleosomal binding domain 2
16673557	4.26	5.51	0.3	0.19	-2.38	4.38E-09	1.48E-06	C1orf112	chromosome 1 open reading frame 112
16792519	4.18	5.43	0.25	0.29	-2.38	3.81E-08	6.36E-06	POLE2	polymerase (DNA directed), epsilon 2, accessory subunit
16845172	4.57	5.82	0.28	0.26	-2.38	6.79E-08	9.32E-06	PSMC3IP	PSMC3 interacting protein
17122274	7.22	8.47	0.27	0.18	-2.38	1.28E-08	3.17E-06		
17005396	5.15	6.41	0.26	0.23	-2.39	1.07E-08	2.78E-06	GMNN	geminin, DNA replication inhibitor
16788142	4.49	5.74	0.24	0.12	-2.39	3.56E-10	3.52E-07	VRK1; LINC00618	vaccinia related kinase 1; long intergenic non-protein coding RNA 618
16701398	3.33	4.59	0.38	0.43	-2.39	1.79E-05	0.0005		
16890490	6.78	8.04	0.26	0.18	-2.39	2.47E-09	1.06E-06	ATIC	5-aminoimidazole-4-carboxamide ribonucleotide formyltransferase/IMP cyclohydrolas
16813342	6.49	7.75	0.27	0.35	-2.39	8.29E-08	1.05E-05	PRC1	protein regulator of cytokinesis 1
16728287	4.93	6.19	0.51	0.42	-2.39	1.05E-05	0.0003	ANO1	anoctamin 1, calcium activated chloride channel
16717676	0.84	2.1	0.3	0.45	-2.4	1.36E-06	7.29E-05		
16717394	1.11	2.37	0.2	0.59	-2.4	9.07E-05	0.0018	SLC25A28	solute carrier family 25 (mitochondrial iron transporter), member 28
16877473	5.75	7.01	0.45	0.3	-2.4	1.26E-06	6.95E-05	GEN1	GEN1 Holliday junction 5 flap endonuclease
16667206	4.8	6.06	0.18	0.33	-2.4	4.41E-08	7.08E-06	CCDC18	coiled-coil domain containing 18

16996722	5.07	6.34	0.28	0.17	-2.4	7.80E-09	2.26E-06	CENPK	centromere protein K
16811684	0.82	2.08	0.28	0.56	-2.4	5.12E-06	0.0002	CYP1A1	cytochrome P450, family 1, subfamily A, polypeptide 1
16919044	5.53	6.8	0.17	0.09	-2.41	2.76E-12	1.53E-08	RBL1	retinoblastoma-like 1
16830912	5.87	7.14	0.31	0.28	-2.41	8.72E-08	1.08E-05		
16758336	5.89	7.17	0.24	0.2	-2.42	1.16E-09	7.24E-07	KNTC1	kinetochore associated 1
16996612	1.79	3.06	0.49	0.35	-2.42	4.87E-06	0.0002	LOC105378992	uncharacterized LOC105378992
16819813	2.82	4.1	0.66	0.56	-2.42	0.0001	0.0019	LINC00920	long intergenic non-protein coding RNA 920
16867784	1.27	2.55	0.56	0.96	-2.42	0.0023	0.022	C3	complement component 3
17001299	1.48	2.76	0.14	0.52	-2.42	4.61E-07	3.40E-05	DPYSL3	dihydropyrimidinase-like 3
16992096	5.43	6.71	0.17	0.37	-2.42	6.15E-08	8.77E-06	SPDL1	spindle apparatus coiled-coil protein 1
16698984	3.09	4.37	0.46	0.83	-2.42	0.0009	0.0108	NEK2	NIMA-related kinase 2
16926087	2.24	3.52	0.37	0.37	-2.43	7.00E-07	4.55E-05	WDR4	WD repeat domain 4
16965190	2.16	3.44	0.24	0.27	-2.43	5.72E-09	1.80E-06	CC2D2A	coiled-coil and C2 domain containing 2A
16773946	6.72	8	0.23	0.13	-2.43	1.88E-10	2.59E-07	RFC3	replication factor C subunit 3
17065045	1.05	2.33	0.48	0.54	-2.43	3.23E-05	0.0008		
17016506	8.34	9.62	0.45	0.2	-2.43	1.19E-07	1.35E-05	HIST1H4L	histone cluster 1, H4l
17126288	4.94	6.23	1.08	0.44	-2.44	0.0019	0.0188		
16882544	4.84	6.13	0.74	0.32	-2.44	4.82E-05	0.0011	LOC102724642	anaphase-promoting complex subunit 1-like
16886717	8.21	9.5	0.56	0.23	-2.45	5.06E-06	0.0002	GALNT5	polypeptide N-acetylgalactosaminyltransferase 5
16764758	4.29	5.58	0.34	0.56	-2.45	1.61E-05	0.0005	GALNT6	polypeptide N-acetylgalactosaminyltransferase 6
17118948	1.92	3.22	0.4	0.29	-2.45	1.36E-06	7.31E-05		
16901957	6.46	7.76	0.48	0.38	-2.46	1.12E-06	6.37E-05	CKAP2L	cytoskeleton associated protein 2-like
17109042	4.66	5.96	0.32	0.25	-2.46	1.52E-07	1.59E-05	MID1	midline 1
17109211	3.64	4.94	0.25	0.24	-2.46	7.25E-09	2.13E-06	FANCB	Fanconi anemia complementation group B
16673983	5.47	6.77	0.44	0.17	-2.47	9.86E-07	5.87E-05	DARS2	aspartyl-tRNA synthetase 2, mitochondrial
16798801	5.82	7.13	0.42	0.32	-2.48	4.91E-07	3.55E-05	ARHGAP11B	Rho GTPase activating protein 11B
16912379	6.96	8.27	0.4	0.46	-2.48	3.68E-06	0.0002	TPX2	TPX2, microtubule-associated
16698947	5.46	6.77	0.42	0.82	-2.48	4.41E-05	0.001		
16843429	3.24	4.55	0.31	0.52	-2.48	3.82E-06	0.0002	SLFN13	schlafen family member 13
17005600	2.02	3.33	0.5	0.44	-2.48	6.95E-06	0.0002	HIST1H2BH	histone cluster 1, H2bh
16761843	3.88	5.19	0.84	0.69	-2.48	0.003	0.0272	ARHGDIB	Rho GDP dissociation inhibitor (GDI) beta
16996545	4.75	6.06	0.26	0.46	-2.48	8.38E-07	5.22E-05	DEPDC1B	DEP domain containing 1B
17119498	3.84	5.16	0.55	0.34	-2.49	1.42E-05	0.0004		
17119536	3.84	5.16	0.55	0.34	-2.49	1.42E-05	0.0004		
17119598	3.84	5.16	0.55	0.34	-2.49	1.42E-05	0.0004		
17023551	3.09	4.4	0.42	0.52	-2.49	3.72E-06	0.0002	EPB41L2	erythrocyte membrane protein band 4.1-like 2
16981444	1.67	2.99	0.47	0.34	-2.49	1.39E-06	7.41E-05	AADAT	aminoadipate aminotransferase
16801557	4.79	6.11	0.38	0.62	-2.49	2.29E-05	0.0006	CCNB2	cyclin B2
17075478	3.76	5.07	0.82	0.6	-2.49	0.0007	0.0086	LOXL2; ENTPD4	lysyl oxidase-like 2; ectonucleoside triphosphate diphosphohydrolase 4
16984725	2.04	3.36	0.45	0.32	-2.49	3.98E-06	0.0002	LOC257396	uncharacterized LOC257396
16913441	1.87	3.19	0.33	0.57	-2.49	2.96E-05	0.0007	NNAT	neuronatin
17121372	1.57	2.9	0.52	0.7	-2.5	0.0004	0.0053		
16686796	5.55	6.87	0.38	0.25	-2.5	5.47E-08	8.11E-06	STIL	SCL/TAL1 interrupting locus
17093397	3.17	4.49	0.33	0.23	-2.5	4.43E-08	7.08E-06	KIF24	kinesin family member 24
16937505	5.48	6.81	0.43	0.23	-2.51	1.43E-07	1.53E-05	FANCD2	Fanconi anemia complementation group D2
16962523	2.53	3.86	0.31	0.62	-2.51	7.63E-05	0.0015	RPL39L	ribosomal protein L39-like
16670377	5.85	7.18	0.31	0.24	-2.52	2.98E-08	5.26E-06	HIST2H4B; HIST2H	histone cluster 2, H4b; histone cluster 2, H4a
16692626	5.85	7.18	0.31	0.24	-2.52	2.98E-08	5.26E-06	HIST2H4B; HIST2H	histone cluster 2, H4b; histone cluster 2, H4a
17120356	2.03	3.36	0.47	0.79	-2.52	0.0003	0.0042		
16849379	5.83	7.17	0.33	0.21	-2.52	2.41E-09	1.06E-06	TK1	thymidine kinase 1, soluble
16766318	5.08	6.41	0.24	0.28	-2.53	8.88E-09	2.46E-06	PRIM1	primase, DNA, polypeptide 1 (49kDa)
16802204	6.07	7.41	0.3	0.12	-2.53	6.30E-10	4.77E-07	ZWILCH	zwilch kinetochore protein
16877956	4.41	5.74	0.43	0.74	-2.53	0.0003	0.0039	CENPA	centromere protein A
16868576	6.26	7.6	0.16	0.2	-2.54	1.59E-11	6.40E-08	DNMT1	DNA (cytosine-5-)-methyltransferase 1
16834056	4.31	5.66	0.35	0.38	-2.54	3.27E-07	2.70E-05	CDC6	cell division cycle 6
17005589	4.99	6.34	0.32	0.37	-2.54	9.30E-08	1.14E-05	HIST1H2AE	histone cluster 1, H2ae
16887840	3.5	4.85	0.4	0.4	-2.54	2.01E-07	1.93E-05	CDCA7	cell division cycle associated 7
16949261	1.6	2.95	0.46	0.44	-2.55	1.69E-05	0.0005		
16964000	3.42	4.77	0.23	0.38	-2.55	4.84E-08	7.56E-06	TACC3	transforming, acidic coiled-coil containing protein 3
16818600	4.85	6.2	0.22	0.21	-2.55	2.28E-10	2.82E-07	ORC6	origin recognition complex subunit 6
16662648	4.51	5.87	0.43	0.41	-2.55	3.03E-06	0.0001	CDCA8	cell division cycle associated 8
17058617	5.38	6.73	0.23	0.19	-2.55	1.67E-10	2.52E-07	RFC2	replication factor C subunit 2
16733719	3.35	4.7	0.31	0.38	-2.55	9.93E-07	5.88E-05	GLB1L2	galactosidase beta 1 like 2
16906571	2.32	3.67	0.78	0.39	-2.56	0.0002	0.0031	STAT4	signal transducer and activator of transcription 4
16868752	2.41	3.76	0.51	0.59	-2.56	0.0005	0.0061	S1PR5	sphingosine-1-phosphate receptor 5
16953279	3.39	4.74	0.39	0.53	-2.56	3.38E-06	0.0001	CDC25A	cell division cycle 25A
17075992	4.32	5.68	0.48	0.51	-2.56	4.85E-05	0.0011	LINC00589	long intergenic non-protein coding RNA 589
17123026	1.66	3.01	0.34	0.41	-2.56	6.40E-06	0.0002		
16798142	2.42	3.78	0.31	0.33	-2.57	1.07E-06	6.15E-05	SNRPN	small nuclear ribonucleoprotein polypeptide N
16889268	1.31	2.66	0.53	0.76	-2.57	0.0006	0.0074	AOX1	aldehyde oxidase 1
16726790	3.86	5.22	0.21	0.17	-2.57	6.83E-11	1.72E-07	POLA2	polymerase (DNA directed), alpha 2, accessory subunit
16711695	0.9	2.26	0.39	1.05	-2.57	0.001	0.0112	SFTA1P	surfactant associated 1, pseudogene
16911149	2.9	4.26	0.32	0.37	-2.57	1.18E-06	6.63E-05	PCNA-AS1	PCNA antisense RNA 1
17061189	3.38	4.75	0.54	0.47	-2.58	1.91E-05	0.0005	NAPEPLD	N-acyl phosphatidylethanolamine phospholipase D
16828826	5.76	7.13	0.37	0.46	-2.59	1.20E-06	6.70E-05	COTL1	coactosin-like F-actin binding protein 1
17016503	9.55	10.92	0.37	0.25	-2.59	8.01E-09	2.30E-06	HIST1H3I	histone cluster 1, H3i
16755750	4.15	5.53	0.41	0.36	-2.59	4.67E-07	3.42E-05	UTP20	UTP20 small subunit (SSU) processome component
17064135	6.32	7.7	0.58	0.24	-2.6	9.55E-06	0.0003	PDIA4	protein disulfide isomerase family A, member 4
16844312	7.55	8.93	0.6	0.53	-2.6	1.58E-05	0.0005	TOP2A	topoisomerase (DNA) II alpha
16889251	3.39	4.77	0.29	0.41	-2.6	1.70E-07	1.71E-05	SGOL2	shugoshin-like 2 (S. pombe)

16879863	5.32	6.7	0.49	0.45	-2.61	1.81E-06	9.00E-05	EPCAM	epithelial cell adhesion molecule
16870131	2.86	4.25	0.55	0.29	-2.61	5.34E-06	0.0002	HAUS8	HAUS augmin like complex subunit 8
16663958	5.03	6.41	0.4	0.49	-2.61	4.14E-06	0.0002	KIF2C	kinesin family member 2C
16904780	3.1	4.49	0.44	0.3	-2.61	9.31E-07	5.60E-05	SPC25	SPC25, NDC80 kinetochore complex component
16804902	4.06	5.45	0.29	0.29	-2.61	9.31E-09	2.55E-06	BLM	Bloom syndrome, RecQ helicase-like
16945101	3.86	5.25	0.5	0.48	-2.61	5.95E-06	0.0002	MCM2	minichromosome maintenance complex component 2
16927052	4.19	5.58	0.28	0.34	-2.61	1.15E-08	2.94E-06	CDC45	cell division cycle 45
16939960	3.8	5.19	0.43	0.37	-2.61	1.26E-07	1.41E-05	KIF15	kinesin family member 15
16914315	3.4	4.79	0.44	0.68	-2.62	4.28E-05	0.001	UBE2C	ubiquitin-conjugating enzyme E2C
16984032	6.37	7.77	0.34	0.3	-2.63	5.19E-08	7.85E-06	SKP2	S-phase kinase-associated protein 2, E3 ubiquitin protein ligase
17045198	8.14	9.54	0.44	0.3	-2.64	7.64E-08	9.96E-06	ANLN	anillin actin binding protein
16747014	4.8	6.2	0.36	0.31	-2.64	8.82E-08	1.09E-05	RAD51AP1	RAD51 associated protein 1
17096205	2.87	4.27	0.43	0.33	-2.64	2.06E-06	9.98E-05	ZNF367	zinc finger protein 367
16985599	5.17	6.57	0.37	0.79	-2.64	9.65E-05	0.0018	CCNB1	cyclin B1
16852025	5.98	7.38	0.32	0.31	-2.65	3.17E-08	5.53E-06	FHOD3	formin homology 2 domain containing 3
16799637	3.44	4.85	0.6	0.32	-2.66	1.88E-06	9.25E-05	RAD51	RAD51 recombinase
16807605	2.43	3.84	0.47	0.31	-2.66	3.96E-07	3.10E-05	OIP5	Opa interacting protein 5
17016363	0.43	1.84	0.44	0.54	-2.67	1.44E-06	7.62E-05	HIST1H3B	histone cluster 1, H3b
16951485	5.02	6.44	0.34	0.3	-2.67	8.66E-08	1.07E-05	SGOL1	shugoshin-like 1 (S. pombe)
16732755	2.23	3.65	0.3	0.42	-2.67	3.67E-07	2.92E-05	GRAMD1B	GRAM domain containing 1B
16683105	4.7	6.12	0.53	0.4	-2.67	1.61E-05	0.0005	HSPG2	heparan sulfate proteoglycan 2
17117441	5	6.42	0.72	0.93	-2.67	7.74E-05	0.0015	MIG7	mig-7
16997676	2.58	4	0.85	0.87	-2.67	0.0025	0.0235	MTRNR2L2	MT-RNR2-like 2
16845794	3.48	4.9	0.18	0.53	-2.67	3.62E-07	2.90E-05	KIF18B	kinesin family member 18B
16928965	0.78	2.21	0.47	1.06	-2.68	0.0011	0.0126		
16664243	2.73	4.16	0.4	0.24	-2.69	2.27E-08	4.36E-06	RAD54L	RAD54-like (S. cerevisiae)
16873087	3.8	5.23	0.63	0.52	-2.69	2.50E-05	0.0006	KCNN4	potassium channel, calcium activated intermediate/small conductance subfamily N alp
16699739	4.8	6.23	0.3	0.22	-2.7	7.36E-10	5.38E-07	PARP1	poly(ADP-ribose) polymerase 1
16793190	6.26	7.7	0.3	0.17	-2.7	1.30E-09	7.75E-07	WDHD1	WD repeat and HMG-box DNA binding protein 1
16708910	3.86	5.3	0.36	0.49	-2.71	1.22E-06	6.78E-05	SFR1	SWI5-dependent homologous recombination repair protein 1
17016393	0.42	1.87	0.17	0.46	-2.72	4.69E-08	7.40E-06	HIST1H1D	histone cluster 1, H1d
16707468	6.64	8.09	0.58	0.24	-2.72	3.62E-07	2.90E-05	KIF11	kinesin family member 11
17086167	5.45	6.89	0.17	0.25	-2.72	3.26E-10	3.46E-07	CEP78	centrosomal protein 78kDa
17119464	3.2	4.65	0.56	0.4	-2.72	4.03E-06	0.0002		
16957951	4.96	6.4	0.32	0.3	-2.73	6.91E-08	9.44E-06	POLQ	polymerase (DNA directed), theta
16697660	3.01	4.46	0.53	0.53	-2.73	9.35E-06	0.0003	MIR181B1	microRNA 181b-1
16963113	1.7	3.15	0.49	0.61	-2.73	2.06E-05	0.0006	APOD	apolipoprotein D
16911212	6.35	7.8	0.19	0.07	-2.73	1.41E-12	1.13E-08	MCM8	minichromosome maintenance 8 homologous recombination repair factor
16691883	4.22	5.67	0.46	0.8	-2.74	8.53E-05	0.0017	FAM72D; FAM72C	family with sequence similarity 72, member D; family with sequence similarity 72, mer
16731654	1.99	3.45	0.55	0.99	-2.74	0.0014	0.0152	TAGLN	transgelin
16972616	4.79	6.25	0.43	0.45	-2.74	1.16E-06	6.56E-05	NEIL3	nei-like DNA glycosylase 3
16922584	4.64	6.1	0.25	0.33	-2.75	6.69E-09	2.05E-06	CHAF1B; MORC3	chromatin assembly factor 1, subunit B (p60); MORC family CW-type zinc finger 3
16802519	6.77	8.23	0.46	0.38	-2.75	2.52E-07	2.25E-05	KIF23	kinesin family member 23
16719515	7.74	9.2	0.38	0.37	-2.75	9.65E-08	1.17E-05	MKI67	marker of proliferation Ki-67
16777278	5.69	7.15	0.41	0.24	-2.75	7.63E-08	9.96E-06	SKA3	spindle and kinetochore associated complex subunit 3
17018497	2.42	3.88	0.53	0.28	-2.76	4.81E-07	3.50E-05	FKBP5	FK506 binding protein 5
16707551	6.15	7.61	0.4	0.42	-2.76	3.77E-07	2.99E-05	CEP55	centrosomal protein 55kDa
16799315	8.18	9.65	0.47	0.46	-2.76	1.50E-06	7.85E-05	THBS1	thrombospondin 1
16735010	2.96	4.43	0.34	0.47	-2.76	6.79E-07	4.46E-05	PRKCDBP	protein kinase C, delta binding protein
17064939	5.19	6.66	0.43	0.2	-2.77	4.23E-08	6.87E-06	NCAPG2	non-SMC condensin II complex subunit G2
17016486	2.21	3.68	0.5	0.73	-2.77	3.54E-05	0.0008	HIST1H2BL	histone cluster 1, H2bl
16960114	6.06	7.53	0.47	0.29	-2.77	2.30E-07	2.12E-05	PLOD2	procollagen-lysine, 2-oxoglutarate 5-dioxygenase 2
16835797	2.99	4.46	0.45	0.46	-2.78	3.13E-06	0.0001	EME1	essential meiotic structure-specific endonuclease 1
16981219	3.71	5.19	0.58	0.56	-2.79	2.66E-05	0.0007	DDX60	DEAD (Asp-Glu-Ala-Asp) box polypeptide 60
17067102	4.52	6	0.33	0.58	-2.79	1.16E-06	6.56E-05	CDCA2	cell division cycle associated 2
16839425	1.48	2.96	0.36	0.62	-2.79	1.60E-05	0.0005	SLC43A2	solute carrier family 43 (amino acid system L transporter), member 2
17080595	4.1	5.59	0.48	0.39	-2.81	1.81E-06	9.02E-05	DSCC1	DNA replication and sister chromatid cohesion 1
16746379	5.1	6.6	0.16	0.1	-2.82	3.05E-13	3.95E-09	NCAPD3	non-SMC condensin II complex subunit D3
16966285	1.95	3.44	0.17	0.88	-2.82	0.0001	0.002	UCHL1	ubiquitin C-terminal hydrolase L1
16877762	3.98	5.47	0.24	0.25	-2.82	4.44E-10	3.88E-07	CENPO	centromere protein O
17093743	3.17	4.67	0.24	0.43	-2.83	6.00E-08	8.62E-06	TPM2	tropomyosin 2 (beta)
16852312	5	6.5	0.43	0.35	-2.83	2.20E-07	2.05E-05	SKA1	spindle and kinetochore associated complex subunit 1
17070381	3.22	4.72	0.47	0.22	-2.83	5.22E-08	7.86E-06	LRRCC1	leucine rich repeat and coiled-coil centrosomal protein 1
17023484	3.94	5.44	0.38	0.46	-2.83	1.81E-07	1.80E-05	ARHGAP18	Rho GTPase activating protein 18
17078134	3.96	5.46	0.28	0.45	-2.83	2.54E-07	2.26E-05	EYA1	EYA transcriptional coactivator and phosphatase 1
16669212	4.07	5.58	0.39	0.22	-2.83	1.30E-08	3.18E-06	TTF2	transcription termination factor, RNA polymerase II
16784299	5.15	6.65	0.41	0.69	-2.83	9.33E-06	0.0003	CDKN3	cyclin-dependent kinase inhibitor 3
16869588	3.14	4.65	0.39	0.17	-2.84	3.46E-09	1.28E-06	ASF1B	anti-silencing function 1B histone chaperone
16979515	7.07	8.58	0.36	0.36	-2.85	4.82E-08	7.54E-06	CCNA2	cyclin A2
16842673	5.07	6.58	0.5	0.45	-2.85	9.32E-07	5.60E-05	SPAG5	sperm associated antigen 5
17016383	5	6.52	0.62	0.3	-2.86	5.61E-07	3.84E-05	HIST1H4D	histone cluster 1, H4d
17057525	1.88	3.39	0.32	0.45	-2.86	1.45E-07	1.54E-05	TNS3	tensin 3
17093724	2.34	3.86	0.27	0.48	-2.89	1.03E-07	1.21E-05	ARHGEF39	Rho guanine nucleotide exchange factor 39
16850107	6.69	8.23	0.48	0.23	-2.9	1.60E-07	1.64E-05	FASN	fatty acid synthase
16906615	3.44	4.98	0.49	0.42	-2.9	1.07E-06	6.15E-05	SDPR	serum deprivation response
16692614	2.04	3.58	0.52	0.48	-2.91	1.17E-06	6.61E-05	HIST2H3D	histone cluster 2, H3d
16817017	6.39	7.94	0.46	0.73	-2.92	4.59E-05	0.001	PLK1	polo-like kinase 1
16755498	5.26	6.81	0.23	0.24	-2.92	8.64E-11	1.83E-07	TMPO	thymopoietin
17099076	5.26	6.81	0.59	0.27	-2.92	8.08E-07	5.09E-05	PTGES	prostaglandin E synthase
16901755	5.46	7	0.33	0.48	-2.92	9.75E-08	1.17E-05	BUB1	BUB1 mitotic checkpoint serine/threonine kinase

17004911	1.93	3.48	0.68	0.49	-2.92	1.32E-05	0.0004		
16943230	0.78	2.33	0.25	1.44	-2.92	0.0021	0.0202	LOC105374003	uncharacterized LOC105374003
16697695	6.07	7.62	0.42	0.59	-2.92	3.21E-06	0.0001	KIF14	kinesin family member 14
16999475	4.18	5.73	1.04	0.76	-2.93	0.0013	0.0143	FBN2	fibrillin 2
17102230	5.1	6.65	0.22	0.16	-2.94	2.65E-12	1.53E-08	POLA1	polymerase (DNA directed), alpha 1, catalytic subunit
16982635	4.27	5.83	0.25	0.26	-2.94	3.30E-10	3.46E-07	TRIP13	thyroid hormone receptor interactor 13
16929855	5.03	6.59	0.61	0.35	-2.94	6.99E-06	0.0002	LGALS1	lectin, galactoside-binding, soluble, 1
16663514	6.07	7.63	0.33	0.7	-2.95	5.98E-06	0.0002	CDC20	cell division cycle 20
17076867	7.11	8.67	0.42	0.18	-2.95	4.12E-08	6.73E-06	PRKDC	protein kinase, DNA-activated, catalytic polypeptide
16799598	5.73	7.29	0.28	0.39	-2.96	1.03E-08	2.73E-06	CASC5	cancer susceptibility candidate 5
16931384	3.78	5.35	0.45	0.69	-2.96	1.11E-05	0.0004	GTSE1	G-2 and S-phase expressed 1
16809748	2.19	3.75	0.58	0.57	-2.97	5.39E-06	0.0002	MNS1	meiosis-specific nuclear structural 1
16979389	4.59	6.16	0.24	0.38	-2.97	6.84E-09	2.07E-06	MAD2L1	MAD2 mitotic arrest deficient-like 1 (yeast)
17068385	2.44	4.02	0.27	0.26	-2.98	7.90E-10	5.60E-07	GINS4	GINS complex subunit 4 (Sld5 homolog)
16978568	5.35	6.93	0.61	0.51	-2.98	6.20E-06	0.0002	CENPE	centromere protein E
16692580	3.17	4.75	0.38	0.29	-2.99	7.13E-09	2.12E-06	HIST2H2BB; HIST2	histone cluster 2, H2bb (pseudogene); histone cluster 2, H3, pseudogene 2
17117537	4.34	5.93	0.58	0.35	-3	2.59E-06	0.0001	LOC100131262	uncharacterized LOC100131262
17055501	1.32	2.9	0.22	0.8	-3	1.68E-05	0.0005	AGR2	anterior gradient 2, protein disulphide isomerase family member
16991859	6.07	7.65	0.33	0.47	-3	8.57E-08	1.07E-05	HMMR	hyaluronan-mediated motility receptor (RHAMM)
16736891	4.3	5.89	0.45	0.62	-3.01	3.60E-06	0.0002	KIF18A	kinesin family member 18A
16745281	1.25	2.84	0.44	0.59	-3.01	1.54E-05	0.0005	MCAM	melanoma cell adhesion molecule
16963241	5.65	7.24	0.29	0.54	-3.01	1.03E-06	6.04E-05	TFRC	transferrin receptor
16723134	0.55	2.14	0.23	1.21	-3.02	0.0006	0.0082	LOC105376603	uncharacterized LOC105376603
17020019	5.84	7.44	0.39	0.37	-3.02	1.53E-07	1.59E-05	MCM3	minichromosome maintenance complex component 3
16838359	3.6	5.2	0.48	0.5	-3.02	9.24E-07	5.59E-05	BIRC5	baculoviral IAP repeat containing 5
16804559	5.83	7.43	0.41	0.13	-3.03	2.26E-09	1.03E-06	FANCI	Fanconi anemia complementation group I
16912192	4.64	6.25	0.17	0.3	-3.04	1.39E-10	2.23E-07	GINS1	GINS complex subunit 1 (Psf1 homolog)
17027710	3.67	5.28	0.39	0.29	-3.05	1.76E-08	3.70E-06		
17030560	3.67	5.28	0.39	0.29	-3.05	1.76E-08	3.70E-06		
17033265	3.67	5.28	0.39	0.29	-3.05	1.76E-08	3.70E-06		
17035356	3.67	5.28	0.39	0.29	-3.05	1.76E-08	3.70E-06		
17038044	3.67	5.28	0.39	0.29	-3.05	1.76E-08	3.70E-06		
17040632	3.67	5.28	0.39	0.29	-3.05	1.76E-08	3.70E-06		
17104484	5.45	7.06	0.55	0.45	-3.05	2.31E-06	0.0001	KIF4A	kinesin family member 4A
16803562	3.8	5.41	0.79	0.51	-3.05	6.89E-05	0.0014	CHRNA5	cholinergic receptor, nicotinic alpha 5
16843374	4.26	5.87	0.4	0.38	-3.06	1.79E-07	1.78E-05	RAD51D	RAD51 paralog D
17051553	5.88	7.5	0.43	0.71	-3.08	2.39E-05	0.0006	CPA4	carboxypeptidase A4
17077191	1.22	2.85	0.49	0.72	-3.09	9.61E-06	0.0003	LOC100507516	uncharacterized LOC100507516
16779546	5.75	7.38	0.4	0.22	-3.1	3.73E-09	1.30E-06	DIAPH3	diaphanous-related formin 3
16714504	3.66	5.31	0.3	0.26	-3.13	3.37E-09	1.27E-06	ZWINT	ZW10 interacting kinetochore protein
17110401	3.32	4.97	0.69	0.3	-3.13	1.66E-05	0.0005	SLC9A7	solute carrier family 9, subfamily A (NHE7, cation proton antiporter 7), member 7
16707695	4.39	6.04	0.3	0.21	-3.13	9.84E-11	1.90E-07	HELLS	helicase, lymphoid-specific
16760621	2.75	4.4	0.36	0.51	-3.13	3.31E-07	2.72E-05	CDCA3	cell division cycle associated 3
16698023	6.01	7.67	0.3	0.19	-3.15	1.80E-10	2.59E-07	UBE2T	ubiquitin conjugating enzyme E2T
16999167	1.6	3.26	0.6	0.66	-3.15	4.37E-05	0.001		
16705159	4.97	6.62	0.67	0.37	-3.16	5.35E-07	3.72E-05	CDK1	cyclin-dependent kinase 1
17125788	7.66	9.32	0.51	0.36	-3.16	4.05E-07	3.13E-05		
16829764	4.51	6.17	0.34	0.41	-3.17	7.72E-08	9.98E-06	GSG2	germ cell associated 2 (haspin)
16965346	7.43	9.1	0.35	0.16	-3.18	6.29E-11	1.72E-07	NCAPG	non-SMC condensin I complex subunit G
16669437	7.35	9.02	0.58	0.35	-3.19	8.39E-07	5.22E-05	HIST2H2BA	histone cluster 2, H2ba (pseudogene)
16667037	4.97	6.65	0.47	0.41	-3.19	1.20E-07	1.35E-05	CDC7	cell division cycle 7
16991741	1.95	3.63	0.63	0.49	-3.2	1.05E-05	0.0003	PTTG1	pituitary tumor-transforming 1
16840902	5.51	7.19	0.39	0.24	-3.2	4.02E-09	1.39E-06	AURKB	aurora kinase B
16850517	5.01	6.69	0.44	0.32	-3.2	1.27E-08	3.15E-06	NDC80	NDC80 kinetochore complex component
16677201	6.04	7.72	0.32	0.32	-3.2	8.16E-10	5.70E-07	DTL	denticleless E3 ubiquitin protein ligase homolog (Drosophila)
16745236	4.12	5.8	0.18	0.3	-3.22	8.15E-11	1.83E-07	H2AFX	H2A histone family, member X
16815090	3.8	5.5	0.37	0.77	-3.23	1.38E-05	0.0004	CCNF	cyclin F
16882975	5.02	6.72	0.25	0.27	-3.26	1.08E-10	1.93E-07	NCAPH	non-SMC condensin I complex subunit H
16702571	5.13	6.84	0.52	0.28	-3.26	1.65E-08	3.61E-06	MCM10	minichromosome maintenance 10 replication initiation factor
16693082	1.83	3.55	0.38	0.91	-3.3	0.0002	0.0037	SELENBP1	selenium binding protein 1
16989636	5.84	7.56	0.49	0.88	-3.3	3.75E-05	0.0009	KIF20A	kinesin family member 20A
17016499	6.91	8.63	0.54	0.38	-3.31	7.55E-08	9.95E-06	HIST1H1B	histone cluster 1, H1b
17024980	1.57	3.3	0.21	0.24	-3.32	5.60E-12	2.70E-08	FBXO5	F-box protein 5
17096904	4.36	6.08	0.25	0.61	-3.32	1.52E-07	1.59E-05	CTNNAL1	catenin (cadherin-associated protein), alpha-like 1
17005797	2.34	4.07	0.42	0.69	-3.32	2.45E-06	0.0001	MIR3143	microRNA 3143
16826160	5.79	7.52	0.31	0.26	-3.32	4.37E-10	3.88E-07	SHCBP1	SHC SH2-domain binding protein 1
16981502	2.7	4.44	0.79	1.08	-3.33	0.0036	0.0315	LOC105377538	uncharacterized LOC105377538
17018408	2.89	4.63	0.84	0.86	-3.35	0.0002	0.0029	SPDEF	SAM pointed domain containing ETS transcription factor
16692636	7.28	9.03	0.49	0.43	-3.36	1.42E-07	1.53E-05	HIST2H2AB	histone cluster 2, H2ab
17067332	3.53	5.28	0.29	0.3	-3.37	5.05E-10	4.06E-07	ESCO2	establishment of sister chromatid cohesion N-acetyltransferase 2
16684144	3.63	5.39	0.77	0.51	-3.38	3.18E-05	0.0008	PTAFR	platelet-activating factor receptor
16913957	5.04	6.8	0.28	0.22	-3.4	4.93E-11	1.48E-07	MYBL2	v-myb avian myeloblastosis viral oncogene homolog-like 2
16793225	5.65	7.41	0.55	0.61	-3.4	3.55E-06	0.0002	DLGAP5	discs, large (Drosophila) homolog-associated protein 5
16675398	4.79	6.55	0.96	0.3	-3.41	1.68E-05	0.0005	CFH	complement factor H
16829369	3.76	5.53	0.28	0.24	-3.41	1.09E-10	1.93E-07	FANCA	Fanconi anemia complementation group A
16804631	5.17	6.96	0.38	0.42	-3.46	1.28E-07	1.42E-05	TICRR	TOPBP1-interacting checkpoint and replication regulator
16847432	5.12	6.91	0.48	0.29	-3.46	6.57E-08	9.10E-06	BRIP1	BRCA1 interacting protein C-terminal helicase 1
16975954	6.09	7.88	0.25	0.27	-3.46	6.42E-11	1.72E-07	SCFD2	sec1 family domain containing 2
16670387	5.29	7.08	0.36	0.43	-3.47	1.10E-08	2.83E-06	HIST2H3A; HIST2H	histone cluster 2, H3a; histone cluster 2, H3c
16692616	5.29	7.08	0.36	0.43	-3.47	1.10E-08	2.83E-06	HIST2H3A; HIST2H	histone cluster 2, H3a; histone cluster 2, H3c

16679650	3.43	5.23	0.45	0.28	-3.48	1.72E-08	3.70E-06	SCCPDH	saccharopine dehydrogenase (putative)
16708728	2.57	4.37	0.45	0.23	-3.48	9.68E-09	2.62E-06	SFXN2	sideroflexin 2
16957170	4.86	6.67	0.33	0.25	-3.51	2.82E-10	3.16E-07	KIAA1524	KIAA1524
17110404	3.93	5.76	0.63	0.39	-3.56	6.20E-06	0.0002	SLC9A7	solute carrier family 9, subfamily A (NHE7, cation proton antiporter 7), member 7
16711413	5.44	7.29	0.64	0.71	-3.6	8.44E-06	0.0003	LOC105376382	uncharacterized LOC105376382
16877019	5.41	7.27	0.16	0.17	-3.62	5.09E-14	2.46E-09	RRM2	ribonucleotide reductase M2
16679411	4.44	6.31	0.39	0.28	-3.66	2.58E-10	2.97E-07	EXO1	exonuclease 1
16664388	1.9	3.78	0.65	0.73	-3.67	1.45E-05	0.0004	CYP4X1	cytochrome P450, family 4, subfamily X, polypeptide 1
16854801	1.98	3.86	0.97	1.07	-3.68	0.0024	0.0228	MIR924	microRNA 924
16798130	2.64	4.54	0.94	0.36	-3.73	4.43E-05	0.001	SNRPN	small nuclear ribonucleoprotein polypeptide N
17016490	1.38	3.28	0.55	0.58	-3.75	3.97E-07	3.10E-05	HIST1H2AJ	histone cluster 1, H2aj
17075776	6.15	8.06	0.55	0.34	-3.75	2.16E-08	4.30E-06	PBK	PDZ binding kinase
16711422	4.2	6.11	0.77	0.73	-3.76	0.0001	0.002	LOC102723629	uncharacterized LOC102723629
17105401	4.98	6.9	0.48	0.16	-3.78	1.81E-09	8.76E-07	CENPI	centromere protein I
17087413	3.29	5.24	0.63	0.36	-3.85	6.79E-07	4.46E-05	GALNT12	polypeptide N-acetylgalactosaminyltransferase 12
16976827	2.28	4.22	0.97	1.24	-3.86	0.0009	0.0103	CXCL5	chemokine (C-X-C motif) ligand 5
17084352	2.24	4.2	1.05	0.36	-3.88	3.33E-05	0.0008		
16909700	5.31	7.29	0.4	0.52	-3.94	3.78E-08	6.33E-06	HJURP	Holliday junction recognition protein
17005858	6.11	8.1	0.61	0.52	-3.97	4.12E-07	3.16E-05	HIST1H2AI	histone cluster 1, H2ai
17005535	1.87	3.86	0.67	0.65	-3.99	6.64E-07	4.39E-05	HIST1H4A	histone cluster 1, H4a
16748496	0.38	2.4	0.27	1.33	-4.06	0.0003	0.0041	APOLD1	apolipoprotein L domain containing 1
17016369	1.48	3.51	0.25	0.78	-4.09	6.49E-06	0.0002	HIST1H2BB	histone cluster 1, H2bb
17059323	6.25	8.29	0.59	0.53	-4.11	4.25E-07	3.24E-05	SEMA3A	sema domain, immunoglobulin domain (Ig), short basic domain, secreted, (semaphorin
17005603	1.69	3.75	0.45	0.6	-4.15	7.55E-08	9.95E-06	HIST1H2BI	histone cluster 1, H2bi
16748346	3.91	5.98	1.09	0.41	-4.2	1.30E-05	0.0004	LOC101928100	uncharacterized LOC101928100
16977868	1.23	3.31	0.41	0.45	-4.22	8.26E-09	2.33E-06	ABCG2	ATP binding cassette subfamily G member 2 (Junior blood group)
16800355	4.93	7.01	0.23	0.36	-4.22	7.13E-11	1.72E-07	WDR76	WD repeat domain 76
16988703	5.58	7.68	0.24	0.2	-4.31	3.28E-13	3.95E-09	LMNB1	lamin B1
16828886	3.32	5.44	0.47	0.36	-4.35	1.57E-09	8.59E-07	GINS2	GINS complex subunit 2 (Psf2 homolog)
16829570	5.33	7.46	1.24	0.83	-4.35	0.0012	0.013	SERPINF1	serpin peptidase inhibitor, clade F (alpha-2 antiplasmin, pigment epithelium derived fa
16903090	5.37	7.51	0.44	0.48	-4.41	1.87E-07	1.83E-05	MCM6	minichromosome maintenance complex component 6
17016496	3.23	5.38	0.84	0.28	-4.41	7.55E-08	9.95E-06	HIST1H2AK	histone cluster 1, H2ak
17016400	6.04	8.21	0.65	0.3	-4.51	3.02E-08	5.32E-06	HIST1H3F	histone cluster 1, H3f
16685165	4.27	6.45	0.43	0.52	-4.52	2.40E-08	4.56E-06	CLSPN	claspin
17016512	1.86	4.08	0.47	0.45	-4.66	6.21E-09	1.93E-06	HIST1H2AM; HIST	histone cluster 1, H2am; histone cluster 1, H3j
16868838	3.43	5.69	0.46	0.51	-4.79	6.93E-09	2.07E-06	SPC24	SPC24, NDC80 kinetochore complex component
16975234	2.6	4.86	0.6	0.82	-4.79	2.89E-07	2.50E-05	TMEM156	transmembrane protein 156
16753663	3.2	5.48	0.9	0.9	-4.85	0.0002	0.0034		
16843480	2.91	5.2	0.8	0.52	-4.89	7.16E-06	0.0003	MMP28	matrix metallopeptidase 28
16813790	0.85	3.14	0.45	0.51	-4.91	1.71E-08	3.70E-06		
17076726	3.14	5.44	0.63	0.65	-4.92	1.58E-07	1.63E-05	PLAT	plasminogen activator, tissue
16971272	3.58	5.91	0.4	0.43	-5	1.18E-09	7.24E-07	EDNRA	endothelin receptor type A
16725041	4.93	7.3	0.56	0.74	-5.17	1.18E-06	6.66E-05	FAM111B	family with sequence similarity 111, member B
17048563	4.36	6.8	0.42	0.37	-5.45	1.87E-10	2.59E-07	PEG10	paternally expressed 10
17016403	5.54	8	0.74	0.48	-5.49	5.69E-08	8.31E-06	HIST1H3G	histone cluster 1, H3g
16890058	3.7	6.3	0.67	0.51	-6.06	2.01E-07	1.93E-05	LOC105373853	uncharacterized LOC105373853
16684080	2.68	5.32	1.66	1.22	-6.24	0.0027	0.0254	IFI6	interferon, alpha-inducible protein 6
17016356	2.45	5.15	0.45	0.51	-6.49	3.59E-10	3.52E-07	HIST1H1A	histone cluster 1, H1a
17104519	1.2	3.92	0.72	1.01	-6.58	5.85E-06	0.0002		
17016509	2.56	5.31	0.59	0.53	-6.73	2.48E-08	4.66E-06	HIST1H3J	histone cluster 1, H3j
17005532	2.67	5.44	1.12	0.57	-6.84	7.91E-05	0.0016	HIST1H3A	histone cluster 1, H3a
17097661	5.01	7.79	1.27	0.42	-6.87	9.01E-06	0.0003	TNC	tenascin C
17016366	3.14	6.01	0.38	0.59	-7.28	1.49E-09	8.57E-07	HIST1H2AB	histone cluster 1, H2ab
16913341	3.06	5.98	1.17	1.06	-7.57	4.30E-05	0.001	MYL9	myosin light chain 9
17005865	6.76	9.71	0.74	0.6	-7.7	1.72E-09	8.59E-07	HIST1H2BM	histone cluster 1, H2bm
16782122	1.65	4.68	1.02	1.71	-8.15	0.0007	0.0085	TRAV20; TRAJ17; T	T cell receptor alpha variable 20; T cell receptor alpha joining 17; T cell receptor delta v
16896561	2.73	5.98	1.26	1.36	-9.54	0.0006	0.008	CYP1B1	cytochrome P450, family 1, subfamily B, polypeptide 1
16966443	1.48	5.36	0.56	0.9	-14.68	2.10E-09	9.82E-07	LOC105374433	uncharacterized LOC105374433

Supplemental Figure 1

A

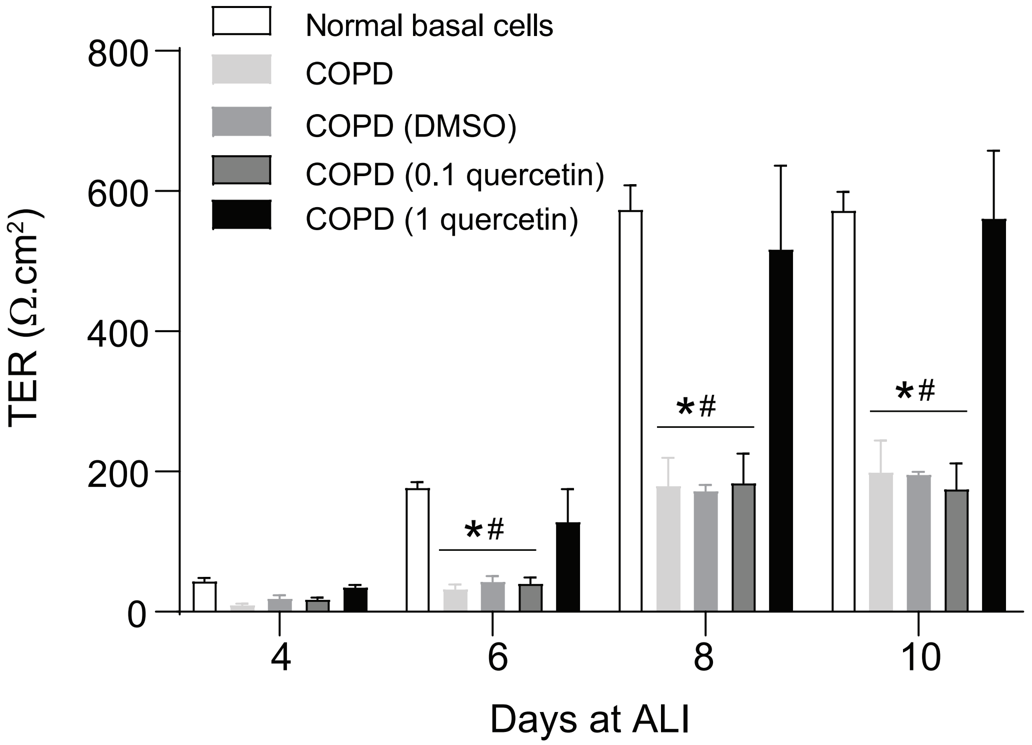

B

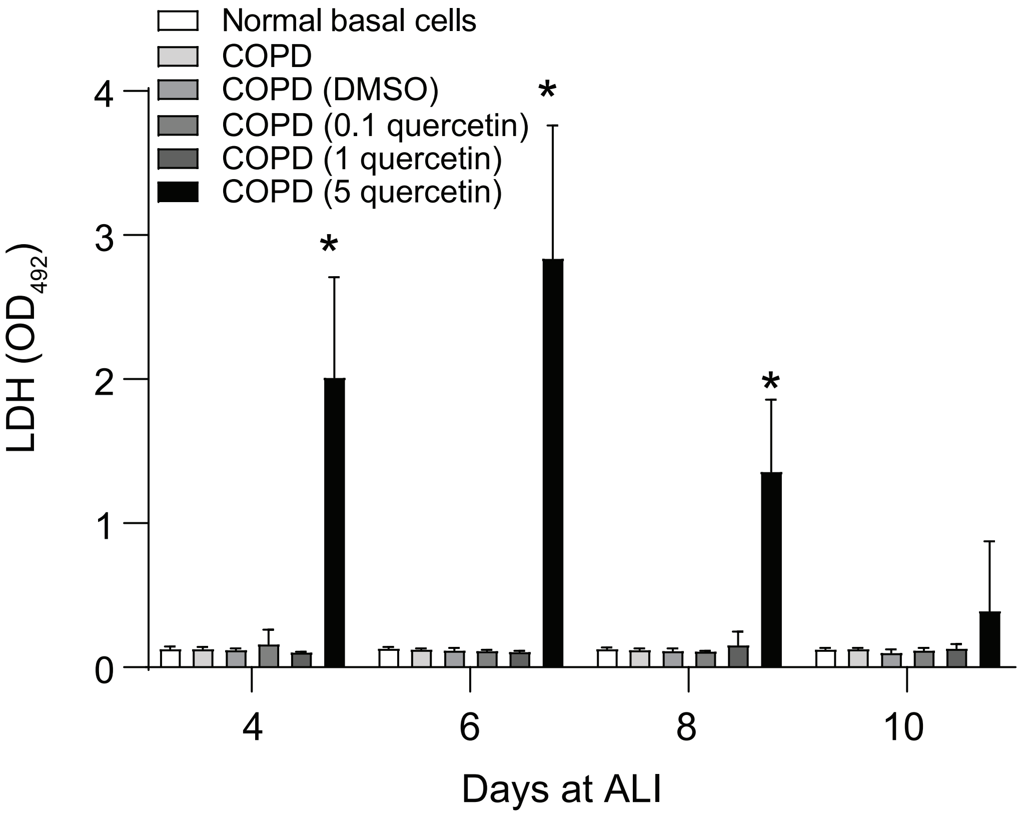

Supplemental Figure 2

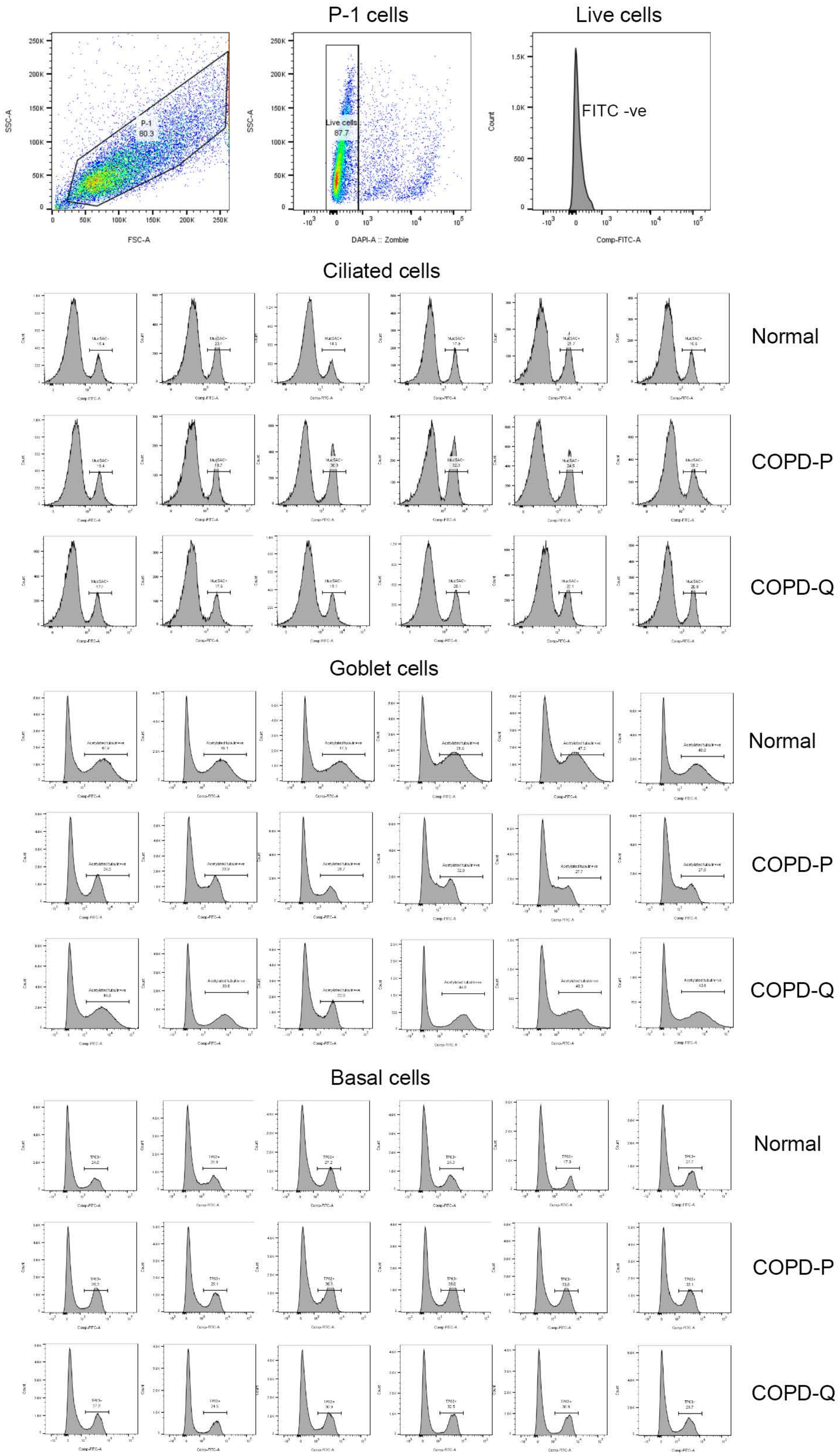

Supplemental Figure 3

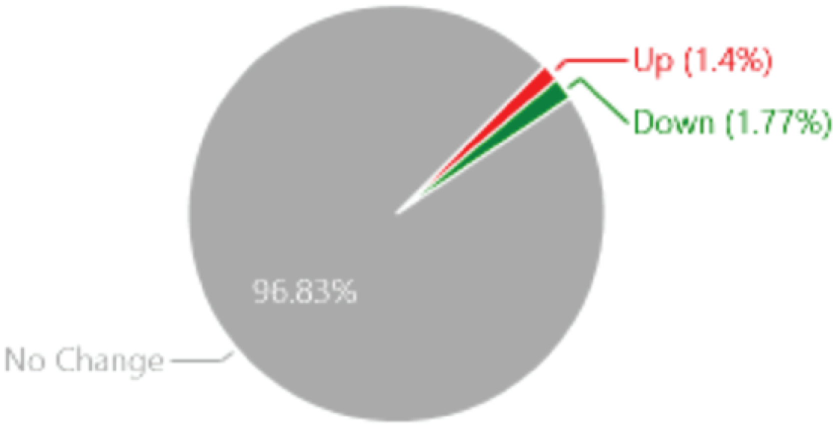

Gene Expression Summary

Total transcripts: 48,226
Filter Criteria:
FDR P-value: <0.05
Fold Change: >2 OR <-2
Transcripts passed filter criteria: 1,527 (3.17%)
Up-regulated: 673 (44.07%)
Down-regulated: 854 (55.93%)
